# De novo macrolide–glycolipid macrolactone hybrids: Synthesis, structure and antibiotic activity of carbohydrate-fused macrocycles

**DOI:** 10.3762/bjoc.10.229

**Published:** 2014-09-17

**Authors:** Richard T Desmond, Anniefer N Magpusao, Chris Lorenc, Jeremy B Alverson, Nigel Priestley, Mark W Peczuh

**Affiliations:** 1 Department of Chemistry, University of Connecticut, 55 N. Eagleville Road, U3060, Storrs, CT 06269, USA, +1-860-486-1605 FAX: +1-860-486-2981; 2 Department of Chemistry and Biochemistry, University of Montana, Missoula, MT 59812, USA

**Keywords:** antibiotic, carbohydrate, exo-anomeric effect, macrolide, structure, synthesis

## Abstract

Natural product-like macrocycles were designed as potential antibacterial compounds. The macrocycles featured a D-glucose unit fused into a 12- or 13-member macrolactone. The rings are connected via the C6’ and anomeric (C1’) positions of the monosaccharide. The new macrocycles/macrolides were characterized by X-ray crystallography. Their structures showed that, in addition to the ester and alkene units, the dihedral angle about the glycosidic linkage (exo-anomeric effect) influenced the overall shape of the molecules. Glycosylation of an available hydroxy group on the macrocycle gave a hybrid macrolide with features common to erythromycin and sophorlipid macrolactone. Weak antibiotic activity (MICs <100 μg/mL) was observed for several of the compounds.

## Introduction

**Figure 1:**
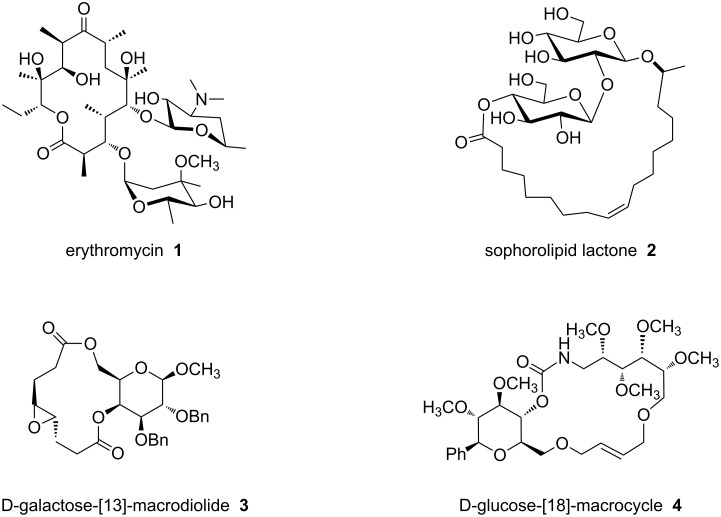
Erythromycin (**1**), the archetypal macrolide; sophorlipid lactone **2**, a glycolipid macrolactone; β-D-galactose fused-[13]-macrodiolide **3** and β-D-glucose-fused [18]-macrocycle **4**.

In contemporary usage, “macrolide” describes any large ring lactone [1]. It was originally coined, however, with reference to a narrower set of compounds: antimicrobial natural products containing a macrolactone ring adorned with deoxygenated carbohydrate residues [2]. Erythromycin (**1**, Figure 1), for example, is an archetypal macrolide due to its molecular structure as well as its antibiotic activity; it is used clinically to treat Gram positive bacterial infections. The mechanism of action of erythromycin is via inhibition of bacterial protein synthesis [3–4]. Sophorolipid lactone **2** [5], on the other hand, represents one example of glycolipid macrolactone natural products. These novel compounds have many potential applications (e.g., food, cosmetics) based on their physical properties; some glycolipid lactones have also been shown to be cytotoxins [6] and **2** also has antibacterial activity [7–8]. Over time, antibiotic use has created a selection pressure that has led to bacterial resistance and a subsequent need for continuous development of new antibiotics. Despite cumbersome syntheses, erythromycin analogs continue to be used as front line antibiotics while the clinical potential of glycolipid macrolactones has yet to be evaluated. The novel structures and biological activities of these natural products provide inspiration for the design and synthesis of new, related compounds that bear a resemblance to them.

**Scheme 1:**
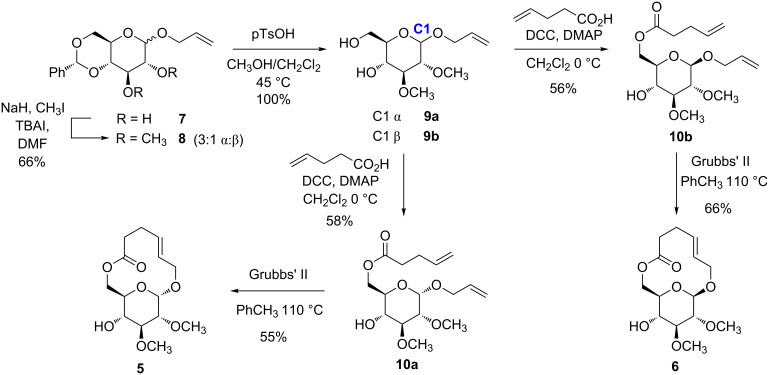
Synthesis of macrolides **5** and **6** by a ring closing metathesis strategy.

We [9–12], among others [13–19], have investigated compounds that blend features of macrolides and glycolipid lactones. These natural product-like compounds fuse the carbohydrate ring to the macrocycle rather than connecting them through a glycosidic linkage. Compounds **3** and **4** in Figure 1 illustrate one approach that has been reported. Here oxygens at the C6 and C4 positions of a pyranose provide two linkage points between the macrocycle and the carbohydrate. Atoms of the carbohydrate are integral to the structure of the macrocycle to form a 13-membered ring in **3** and an 18-membered ring in **4**. In addition to the number of atoms in the ring, the presence of rigidifying planar units and stereocenters collectively govern the shape of a given macrocycle. In fact, we observed that the absolute configuration of C4 of the pyranose ring strongly influenced the shape and reactivity of macrocycle **3** [9]. In **3**, the oxygens at C4 and C6 are both part of ester linkages; in **4** they are a carbamate and ether, respectively. Modest antifungal activity against *C. neoformans* and *A. fumigatis* were also noted for **4** [13]. Here we report on two new natural product-like 12-membered ring macrolides **5** and **6** (Scheme 1) where the pyranose is fused to the macrocycle through the C1’ (glycosidic) and C6’ oxygens. The new macrolides bear a resemblance to sophorolipid lactone **2** and to polyketide macrocycles that contain a tetrahydropyran moiety [20–22]. We report on the synthesis, X-ray crystal structures and antibiotic activities of the new compounds.

## Results and Discussion

The syntheses of **5** and **6** (Scheme 1) generally followed a ring closing metathesis (RCM) strategy that had been established previously [9]. C4,C6-*O*-Benzylidene-protected allyl glucoside **7**, as a mixture of α- and β-anomers, was the starting material for the synthesis. In the first step, the C2 and C3 hydroxy groups were converted to methyl ethers via alkylation with iodomethane in the presence of sodium hydride to give compounds **8a** and **8b** (3:1, 66% combined yield). At this point the α- and β-anomers could be separated by column chromatography. Each anomer was then carried through the remainder of the synthesis separately. Transacetalization of the C4,C6-*O*-benzylidene protecting group in methanol provided diols **9a** and **9b**, respectively in nearly quantitative yields. Chemoselective, DCC-mediated acylation of the primary alcohol group of **9a** and **9b** at 0 °C with pentenoic acid gave **10a** (58%) and **10b** (56%). Compounds **10a** and **10b** were poised for RCM by virtue of the two alkenes present in them. RCM of each one, using the second generation Grubbs catalyst, provided *E*-configured macrolides **5** and **6** in 55 and 66% yield. Both compounds were isolated as crystalline solids after purification by column chromatography. Recrystallization of each, from a mixture of hexanes and ethyl acetate, provided crystals of sufficient quality to determine their structures by X-ray crystallography.

**Figure 2:**
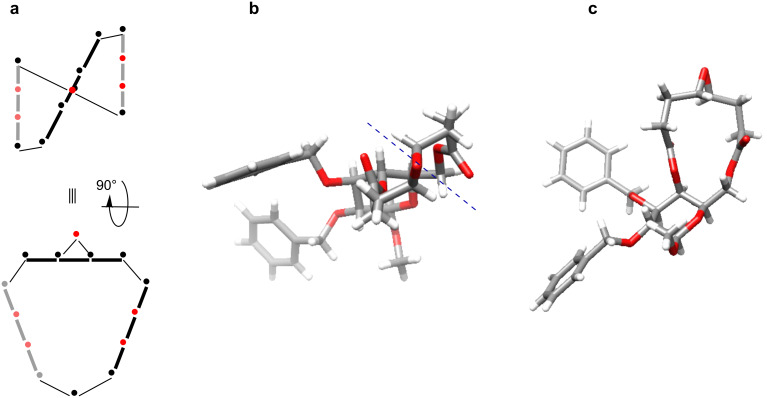
Structure of macrolide **3**; a) schematic representation of **3** emphasizing four-atom planar units of the [13]-macrodiolide motif; b) and c) the structure of **3** from X-ray data that corresponds to the schematic representation. An axis of chirality associated with the topology of the macrocycle is indicated with a dashed line in b).

We are broadly interested in understanding how molecular/conformational features such as planar multi-atom units, stereogenic centers and stereoelectronic effects combine to dictate the “topology” or overall fold of a macrocycle. The structure of β-D-galactose-[13]-macrodiolide **3** [9], derived from X-ray data, originated this line of investigation. It showed that both esters and the epoxide unit are each composed of four coplanar atoms that significantly reduce the number of freely rotatable bonds in the molecule [9–10,12,23–25] and rigidify its structure. The consequence is that the esters, alkene and epoxide units are not coplanar, but are set at angles to each other (Figure 2 ). The result is a twist in the backbone of the macrocycle whose chirality is dictated by the absolute configuration of the C4 stereogenic center. The topology is a defining feature of this family of [13]-macrodiolides. By virtue of the planar chirality, [13]-macrodiolides such as **3** have an axis of chirality associated with them. We were therefore interested to see how the ester and allyl units of **5** and **6** would affect their overall structures. We were equally interested in the role that the glycosidic linkage has in governing each macrocycle’s shape.

**Figure 3:**
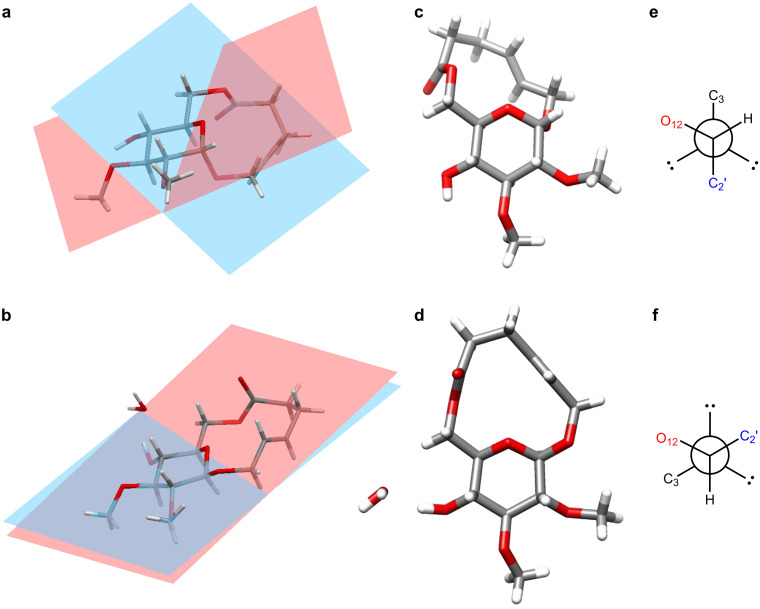
Structures of **5** and **6** from X-ray crystallographic data; a) and b) side views of **5** (a) and **6** (b) with mean plane of macrocycle in blue and D-glucose in red; c) and d) views of **5** and **6** from an angle perpendicular to the D-glucose plane; e) and f) Newman projections, sighting down the glycosidic bond, of **5** and **6**.

The structures of **5** and **6**, derived from X-ray crystallographic data, share several characteristics (Figure 3) [26]. The main structural features for these compounds are the D-glucose ring and the macrocyclic ring. The α-D-glucosyl unit in **5** and the β-D-glucosyl unit in **6** both adopt the common ^4^C_1_ chair conformation of D-pyranosides. Further, the hydroxymethyl group of the D-glucosyl unit (defined as the torsional angle about the C5’–C6’ bond) is in the gt conformation for each structure [27]. An intramolecular hydrogen bond between the C4’ hydroxy group and the C3’ oxygen is also apparent in **5** whereas in **6** the same hydroxy group is hydrogen-bonded to a bound water molecule. Observations made regarding the macrolactone moiety of **5** and **6** references the atom numbering shown in Table 1. Dihedral angles close to 180° for the C4–C5 alkene and the C8 ester indicated that these groups are nearly planar; these units are akin to those that rigidified the [13]-macrodiolide ring in **3** (Figure 2). An additional coplanar, four-atom sequence spanned from C10–C11–O12–C1; it was unanticipated that this unit, which includes the glucosyl ring oxygen, would be planar. Atom C10 of the macrocycle (C6’ using D-glucose numbering) is consequently common to two of the three planar units in the macrocycle. The C9–C10–C11 bond angle of 108–110° indicates that C10 can accommodate both planes and does not distort from the normal sp^3^ hybridization. A schematic rendition of the macrocycle that depicts the planar units and their orientations is also included in Table 1.

**Table 1:** Selected bond angles for **5** and **6** from X-ray crystallographic data.

		**5** (α)	**6** (β)

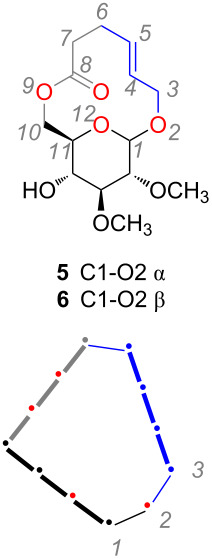	**Planar units:**		
	C3–C4–C5–C6 (alkene)	176.81	179.07
	C7–C8–C9–C10 (ester)	170.19	170.66
	C10–C11–O12–C1	179.24	168.54
	**Dihedrals:**		
	C5–C6–C7–C8	−58.78	−63.23
	C11–O12–C1–O2	60.65	−175.85
	O12–C1–O2–C3 (exo-anomeric)	66.11	75.33
	D-glucose C5’–C6’(C10) rotamer	65.46 (gt)	−66.90 (gt)
	**Angles:**		
	C9–C10–C11	109.92	107.97

The three rigidifying units create a roughly triangular shape to the backbone of the macrocycle (e.g., **6** in Figure 3d) and the relative orientation of these units creates a macrocyclic plane. When comparing **5** and **6**, the relationship between the mean planes defined by the glucose and the macrocycle is clearly different. Specifically, the two planes are set at an angle to each other in **5** whereas in **6** the two planes are essentially coincident. The relationship of the two planes tends to warp macrocycle **5** but not **6**, which is essentially planar. It is the configuration of the anomeric carbon that governs the difference in the structures. First and foremost, the axial (**5**) versus equatorial (**6**) positioning of the anomeric oxygen, and subsequently the aglycone, dictates the orientation of the D-glucose- and macrocyclic planes. Relatedly the exo-anomeric effect [28–29] plays a role in determining the orientation of the backbone of the macrocycle relative to the pyranose. The exo-anomeric effect is a stereoelectronic effect concerned with the donation of electron density from a lone pair on the aglycone oxygen (O2) into the σ* antibonding orbital of the O12–C1 bond. Figure 3e and Figure 3f show the Newman projections for the glycosidic bond showing the antiperiplanar relationship between the ring oxygen (O12) and the lone pair of the aglycone oxygen (O2). This configuration is stabilizing because it enables delocalization of electron density and it must be balanced with the need to accommodate the steric bulk of the aglycone – in this case the macrocyclic ring. In total it is the balancing of a number of small factors such as rigidification by multi-atom planar units, absolute configuration of stereogenic centers and stereoelectronic effects that dictate the observed structures.

Minimum inhibitory concentrations (MICs) against a variety of organisms were determined for macrocycles **5** and **6** to assess their antibiotic activity (Table 2). Notably, the MIC for α-macrolide **5** was <100 μg/mL against *B. subtilis* and *B. anthracis*. Based on this initial activity, we prepared a series of analogs of **5** by derivatizing the C4’ hydroxy group. This was possible by virtue of the original C4,C6 diol **9a**; chemoselective acylation of the primary alcohol (C6’) unit left the C4’ alcohol available for additional reactions. Analogs were prepared under precedented conditions to give **11**–**16** in good yields. Analog **17**, which contains a saturated macrocyle, was also prepared. Among them, only analogs **11** and **16** had MICs that were similar to **5** while the other analogs showed no antibiotic activity. Glycosylated analog **16** combines the features of erythromycin and sophorlipid macrolactone. That is, a glycosylated macrocycle feature that defines the macrolides is added to the cyclic glycolipid macrolactone feature of the sophorlipids. De novo macrolide **16** was active against *B. anthracis*, with a MIC of 115 μg/mL. A 13-membered ring analog of **5** was prepared by acylating **10c** with 5-hexenoyl chloride followed by RCM to give **19**. This compound also had some activity against *S. pyogenes* and *B. subtilis*. Although the mechanism of action of the family of antibiotic macrocycles in Table 2 is not defined, the compounds were designed as protein synthesis inhibitors [3,30]. Compounds **11** and **18** have the lowest MIC values reported here. They are the α- and β-12-membered ring macrocycles with a C4’-*O*-*tert*-butyldimethylsilyl group. MICs as low as 52 μg/mL against *S. aureus, E. faecalis*, and *B. subtillis* were observed. The small data set and low activity of the compounds prevent a QSAR analysis but the influence of a log P effect seems most likely [31–33].

**Table 2:** MIC (μg/mL) values for compounds **5**, **6** and **11**–**19**.

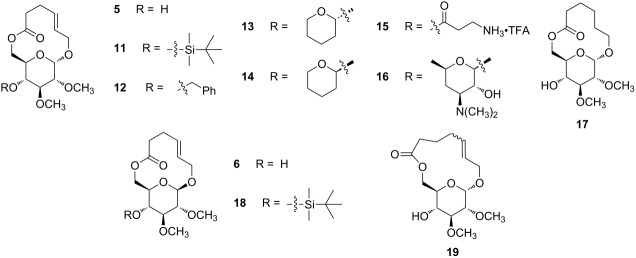											

	**5**	**11**	**12**	**13**	**14**	**15**	**16**	**17**	**6**	**18**	**19**

*S. aureus*	151	**52**	>392	>302	>302	>244	>460	>304	>302	**52**	158
*E. faecalis*	151	>416	>392	>302	>302	>244	>460	>304	302	**52**	158
*S. pyogenes*	151	**104**	>392	>302	>302	–	–	–	151	>208	**80**
*B. subtilis*	**76**	**52**	>392	>302	>302	>244	460	304	302	**52**	**80**
*B. anthracis*	**76**	208	>392	>302	>302	>244	**115**	>304	>302	>208	158
*K. pneumoniae*	>600	–	>392	>302	>302	–	–	–	–	>208	–
*P. aeruginosa*	>600	>416	>392	>302	>302	>244	>460	>304	>302	>208	>316
*E. coli*	>600	–	>392	>302	>302	–	–	–	–	>208	ND
*C. glabrata*	>600	>416	>392	>302	>302	–	–	–	>302	>208	>316
*C. albicans*	151	>416	>392	>302	>302	>244	>460	>304	>302	>208	>316

## Conclusion

We have described the synthesis and characterization of de novo macrolide **16** as a member of a family of related macrocyles that fuse a pyranose monosaccharide to the macrocyclic ring. The new compounds showed modest antibacterial activity against Gram positive organisms. The main conclusion of the work, however, relates the role of the exo-anomeric effect on the low-energy conformation of macrocycles linked through an anomeric center. This weak stereoelectronic effect should be listed with other factors such as ring size, multi-atom planar units, and stereogenic centers as determinants of macrocylic topology. These features will likely play a role in the future design of new macrocycles with specific structures and functions. It also provides a useful basis for developing SAR of macrocyclic natural products.

Crystallographic data for **5** and **6** are in the Cambridge Crystallographic Data Centre (CCDC), No. 1006597 and 1006598. Copies of this information may be obtained free of charge from CCDC, 12 Union Road, Cambridge CB2 1EZ, UK (Fax: +44-1223-336033; web: http://www.ccdc.cam.ac.uk; email: deposit@ccdc.cam.ac.uk).

## Supporting Information

File 1 Characterization data including ^1^H and ^13^C NMR spectra of all new compounds and ORTEP figures for **5** and **6**.

File 2 Experimental procedures and characterization of all new compounds.

## References

[1] Nicolaou K C (1977). Tetrahedron.

[2] Woodward R B (1957). Angew Chem.

[3] Yonath A (2005). Annu Rev Biochem.

[4] Dunkle J A, Xiong L, Mankin A S, Cate J H D (2010). Proc Natl Acad Sci U S A.

[5] Fürstner A, Radkowski K, Grabowski J, Wirtz C, Mynott R (2000). J Org Chem.

[6] Chen J, Song X, Zhang H, Qu Y-b, Miao J-y (2006). Appl Microbiol Biotechnol.

[7] Gupta R, Kumar U S, Prabhune A (2012). Res J Biotechnol.

[8] Kim K, Yoo D, Kim Y, Lee B, Shin D, Kim E-K (2002). J Microbiol Biotechnol.

[9] Fyvie W S, Peczuh M W (2008). J Org Chem.

[10] Fyvie W S, Peczuh M W (2008). Chem Commun.

[11] Ma J, Peczuh M W (2013). J Org Chem.

[12] Ma J, Vannam R, Terwilliger D, Peczuh M W (2014). Tetrahedron Lett.

[13] Ruttens B, Blom P, Van Hoof S, Hubrecht I, Van der Eycken J (2007). J Org Chem.

[14] Blom P, Ruttens B, Van Hoof S, Hubrecht I, Van der Eycken J (2005). J Org Chem.

[15] Horvat Š, Roščić M, Varga-Defterdarović L, Horvat J (1998). J Chem Soc, Perkin Trans 1.

[16] Potopnyk M A, Cmoch P, Jarosz S (2012). Org Lett.

[17] Ajay A, Sharma S, Gupt M P, Bajpai V, Kumar B, Kaushik M P, Konwar R, Ampapathi R S, Tripathi R P (2012). Org Lett.

[18] Allam A, Dupont L, Behr J-B, Plantier-Royon R (2012). Eur J Org Chem.

[19] Billing J F, Nilsson U J (2005). J Org Chem.

[20] Nasir N M, Ermanis K, Clarke P A (2014). Org Biomol Chem.

[21] Crane E A, Scheidt K A (2010). Angew Chem, Int Ed.

[22] Larrosa I, Romea P, Urpi F (2008). Tetrahedron.

[23] Schreiber S L (2000). Science.

[24] Lee D, Sello J K, Schreiber S L (1999). J Am Chem Soc.

[25] Kim Y-k, Arai M A, Arai T, Lamenzo J O, Dean E F, Patterson N, Clemons P A, Schreiber S L (2004). J Am Chem Soc.

[R26] 26Absolute configuration has not been established by anomalous-dispersion effects in diffraction measurements on the crystal. Rather, the enantiomer has been assigned by reference to several chiral centers, present in the starting material (D-glucose), that remain unchanged throughout the synthetic procedure.

[27] Stenutz R, Carmichael I, Widmalm G, Serianni A S (2002). J Org Chem.

[28] Tvaroŝka I, Bleha T (1989). Adv Carbohydr Chem Biochem.

[29] Lemieux R U, Koto S, Voisin D, Szarek W A, Horton D (1979). The Exo-Anomeric Effect. Anomeric Effect: Origin and Consequences.

[30] Maffioli S I, Fabbretti A, Brandi L, Savelsbergh A, Monciardini P, Abbondi M, Rossi T, Donadio S, Gualerzi C O (2013). ACS Chem Biol.

[31] McFarland J W, Berger C M, Froshauer S A, Hayashi S F, Hecker S J, Jaynes B H, Jefson M R, Kamicker B J, Lipinski C A, Lundy K M (1997). J Med Chem.

[32] Puratchikody A, Nagalakshmi G, Doble M (2008). Chem Pharm Bull.

[33] Li F, Mulyana Y, Feterl M, Warner J M, Collins J G, Keene F R (2011). Dalton Trans.

