# Supplementary material for: De novo macrolide–glycolipid macrolactone hybrids: Synthesis, structure and antibiotic activity of carbohydrate-fused macrocycles
Source: Beilstein J Org Chem. 2014 Sep 17;10:2215–21. doi: 10.3762/bjoc.10.229 (PMC4168945; doi:10.3762/bjoc.10.229)
Supplement: File 1 — Characterization data including 1H and 13C NMR spectra of all new compounds and ORTEP figures for 5 and 6. [file Beilstein_J_Org_Chem-10-2215-s001.pdf]

Supporting Information

for

De novo macrolide – glycolipid macrolactone hybrids: Synthesis, structure and antibiotic activity of carbohydrate-fused macrocycles

Richard T. Desmond[1], Anniefer N. Magpusao[1], Chris Lorenc[1], Jeremy B. Alverson[2], Nigel Priestley[2], and Mark W. Peczuh[1]*

Address: [1]Department of Chemistry, University of Connecticut, 55 N. Eagleville Road, U3060, Storrs, CT 06269 USA, +1-860-486-1605 FAX: +1-860-486-2981 and [2]Department of Chemistry and Biochemistry, University of Montana, Missoula, MT 59812, USA

Email: Mark W. Peczuh - mark.peczuh@uconn.edu

Characterization data including ^{1}H and ^{13}C NMR spectra of all new compounds and ORTEP figures for **5** and **6**.

Table of Contents	Page
^{1}H & ^{13}C NMR spectra of α-[12]-macrolactone **5**	S3
^{1}H & ^{13}C NMR spectra of C4-*O*-(*t*-Butyldimethylsilyl)-α-[12]-macrolactone **11**	S5
^{1}H & ^{13}C NMR spectra of C4-*O*-Benzyl-α-[12]-macrolactone **12**	S7
^{1}H & ^{13}C NMR spectra of C4-*O*-(α-tetrahydropyranyl)-α-[12]-macrolactone **13**	S9
^{1}H & ^{13}C NMR spectra of C4-*O*-(β-tetrahydropyranyl)-α-[12]-macrolactone **14**	S11
^{1}H & ^{13}C NMR spectra of C4-*O*-β-alanyl)-α-[12]-macrolactone **15**	S13
^{1}H & ^{13}C NMR spectra of C4-*O*-(β-D-desosaminyl)-α-[12]-macrolactone **16**	S15
^{1}H & ^{13}C NMR spectra of α-[12]-saturated macrolactone **17**	S17
^{1}H & ^{13}C NMR spectra of β-[12]-macrolactone **6**	S19
^{1}H & ^{13}C NMR spectra of C4-*O*-(*tert*-Butyldimethylsilyl)-β-[12]-macrolactone **18**	S21
^{1}H & ^{13}C NMR spectra of α-[13]-macrolactone **19**	S23
^{1}H & ^{13}C NMR spectra of Allyl 4,6-*O*-benzylidene-2,3-di-*O*-methyl-α-D-glucopyranoside **8a**	S25
^{1}H & ^{13}C NMR spectra of Allyl 4,6-*O*-benzylidene-2,3-di-*O*-methyl-β-D-glucopyranoside **8b**	S27
^{1}H & ^{13}C NMR spectra of Allyl 2,3-di-*O*-methyl-α-D-glucopyranoside **9a**	S29
^{1}H & ^{13}C NMR spectra of Allyl 2,3-di-*O*-methyl-β-D-glucopyranoside **9b**	S31
^{1}H & ^{13}C NMR spectra of Allyl 2,3-di-*O*-benzyl-6-*O*-(4-pentenoyl)-α-D-glucopyranoside **10a**	S33
^{1}H & ^{13}C NMR spectra o Allyl 2,3-di-*O*-benzyl-6-*O*-(4-pentenoyl)-β-D-glucopyranoside **10b**	S35
^{1}H & ^{13}C NMR spectra of Allyl 2,3-di-*O*-benzyl-6-*O*-(5-hexenoyl)-α-D-glucopyranoside **10c**	S37
**Figure S1.** ORTEP of compound **5**.	S39
**Figure S2.** ORTEP of compound **6**.	S40

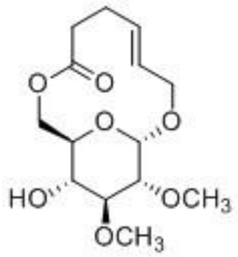

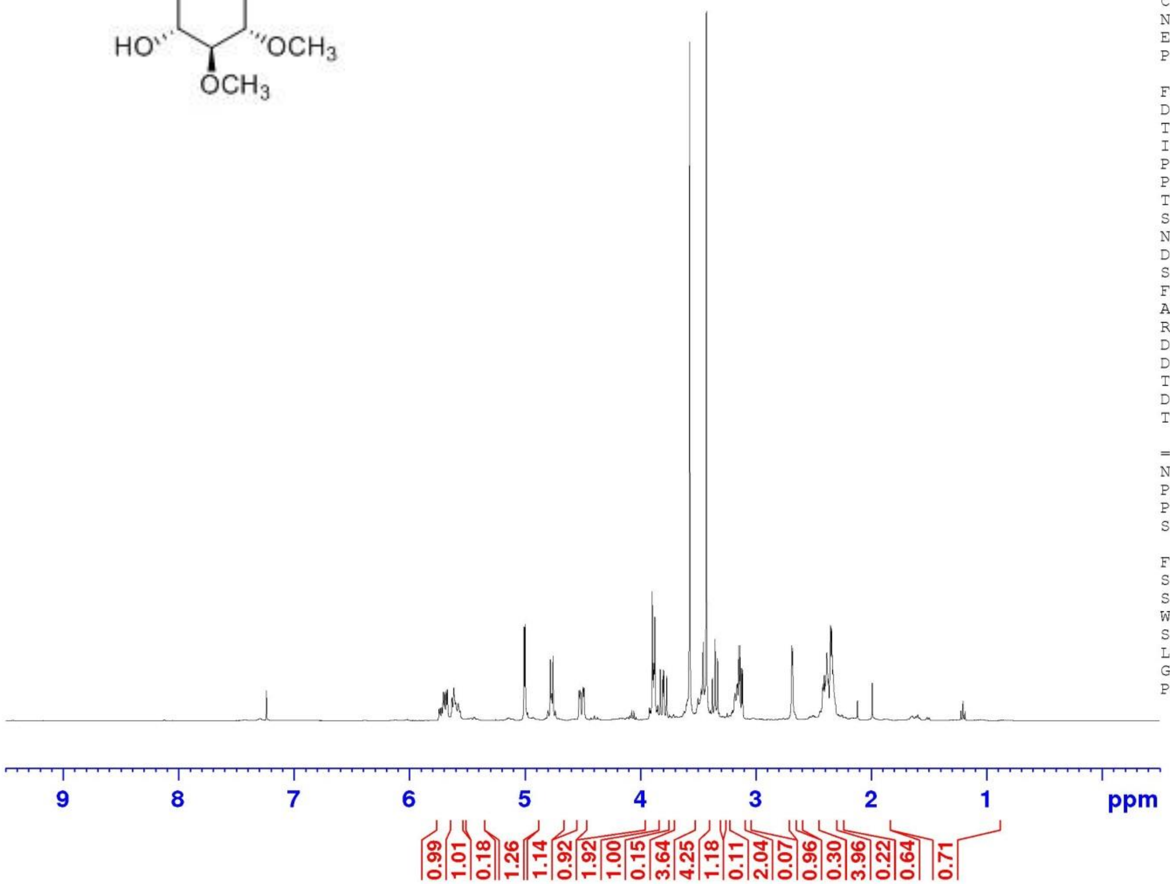


```
Current Data Parameters
NAME        rtd-iii-92-p2
EXPNO                   1
PROCNO                  1

F2 - Acquisition Parameters
Date_            20130127
Time                17.36
INSTRUM             spect
PROBHD    5 mm PABBO BB/
PULPROG              zg30
TD                  48076
SOLVENT             CDCl3
NS                     32
DS                      2
SWH              8012.820 Hz
FIDRES           0.166670 Hz
AQ              2.9999423 sec
RG                     36
DW                 62.400 usec
DE                   6.50 usec
TE                  298.0 K
D1             2.00000000 sec
TD0                     1

======== CHANNEL f1 ========
NUC1                   1H
P1                  15.22 usec
PLW1          12.00000000 W
SFO1          400.1464009 MHz

F2 - Processing parameters
SI                 131072
SF            400.1440180 MHz
WDW                    EM
SSB      0
LB                   0.50 Hz
GB       0
PC                   1.00
```

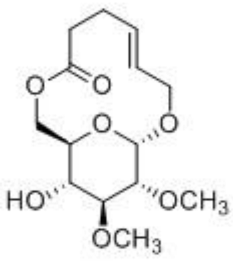

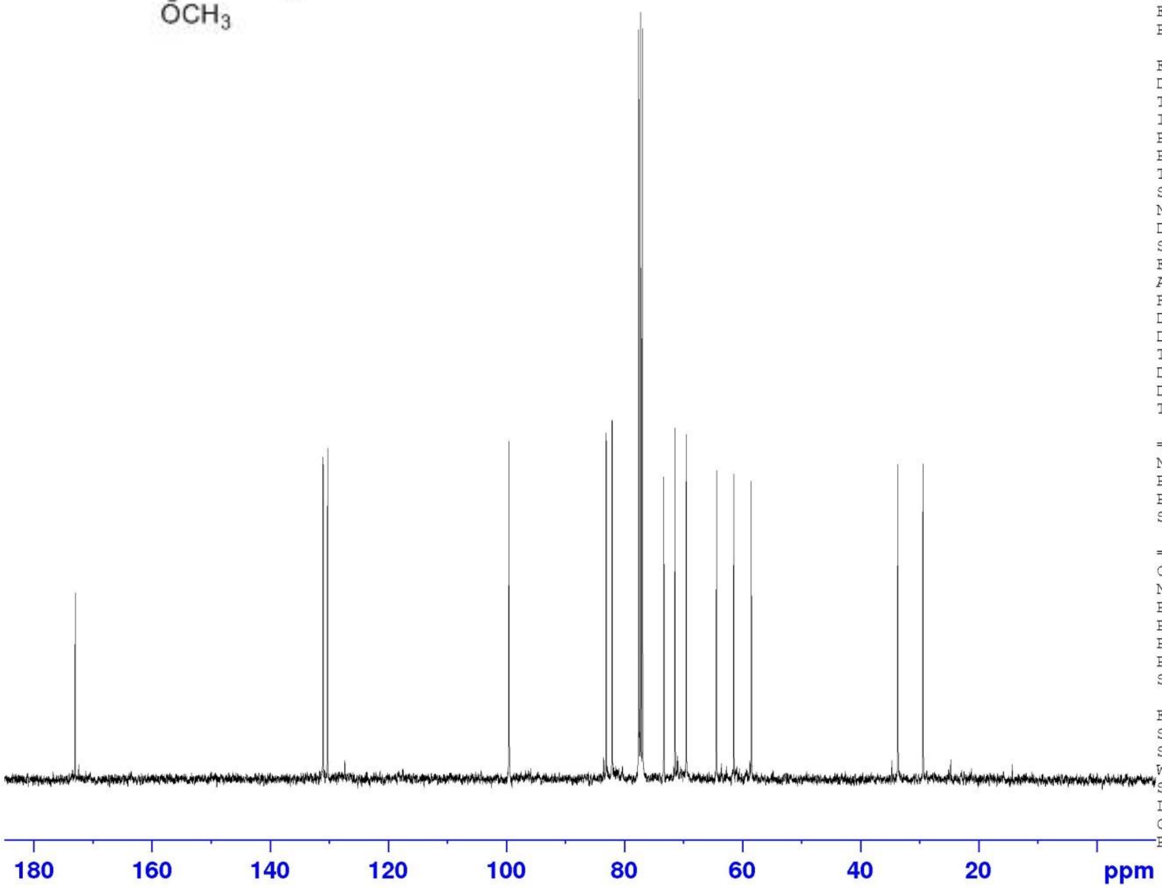


```
Current Data Parameters
NAME        rtd-iii-92-p2
EXPNO                   2
PROCNO                  1

F2 - Acquisition Parameters
Date_           20130127
Time               17.40
INSTRUM            spect
PROBHD    5 mm PABBO BB/
PULPROG          zgpg30
TD                43698
SOLVENT           CDCl3
NS                  256
DS                    4
SWH          24038.461 Hz
FIDRES        0.550104 Hz
AQ           0.9089184 sec
RG                  203
DW               20.800 usec
DE                 6.50 usec
TE                298.0 K
D1           1.50000000 sec
D11          0.03000000 sec
TD0                   1

======== CHANNEL f1 ========
NUC1                 13C
P1                 10.00 usec
PLW1         50.00000000 W
SFO1         100.6263500 MHz

======== CHANNEL f2 ========
CPDPRG[2         waltz16
NUC2                  1H
PCPD2              90.00 usec
PLW2         12.00000000 W
PLW12         0.33333001 W
PLW13         0.27000001 W
SFO2         400.1456006 MHz

F2 - Processing parameters
SI                131072
SF           100.6162754 MHz
WDW                   EM
SSB      0
LB                  2.50 Hz
GB       0
PC                  1.40
```

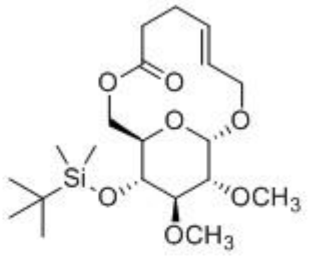
Si
O
O
O
O
O
OCH3
OCH3

11

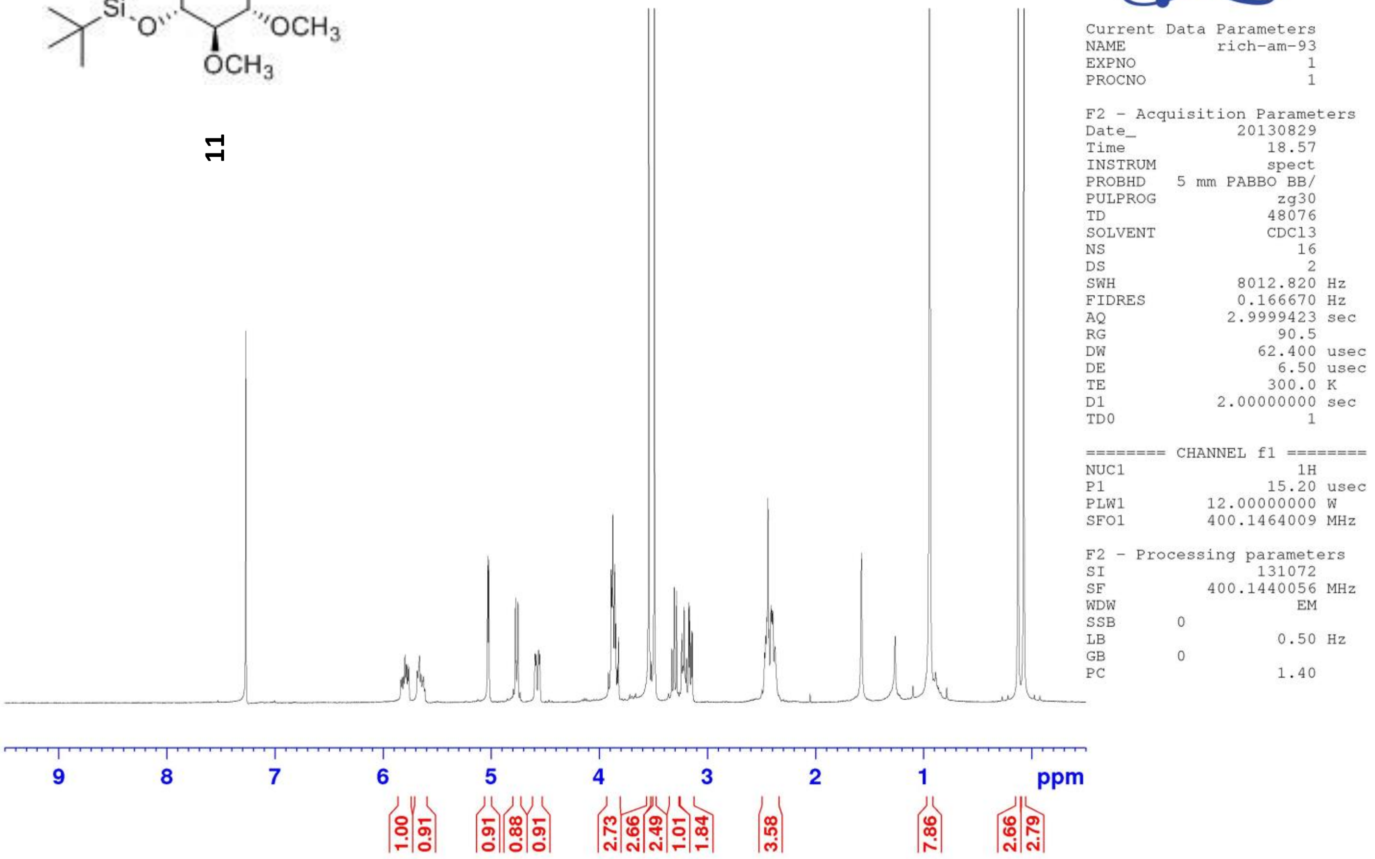
9
8
7
6
5
4
3
2
1
ppm
1.00
0.91
0.91
0.88
0.91
2.73
2.66
2.49
1.01
1.84
3.58
7.86
2.66
2.79
Current Data Parameters
NAME rich-am-93
EXPNO 1
PROCNO 1
F2 - Acquisition Parameters
Date_ 20130829
Time 18.57
INSTRUM spect
PROBHD 5 mm PABBO BB/
PULPROG zg30
TD 48076
SOLVENT CDCl3
NS 16
DS 2
SWH 8012.820 Hz
FIDRES 0.166670 Hz
AQ 2.9999423 sec
RG 90.5
DW 62.400 usec
DE 6.50 usec
TE 300.0 K
D1 2.00000000 sec
TD0 1
======== CHANNEL f1 ========
NUC1 1H
P1 15.20 usec
PLW1 12.00000000 W
SFO1 400.1464009 MHz
F2 - Processing parameters
SI 131072
SF 400.1440056 MHz
WDW EM
SSB 0
LB 0.50 Hz
GB 0
PC 1.40

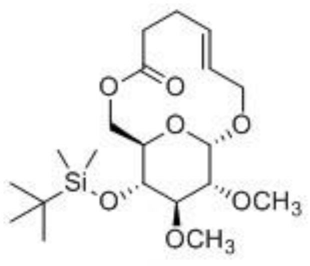

11

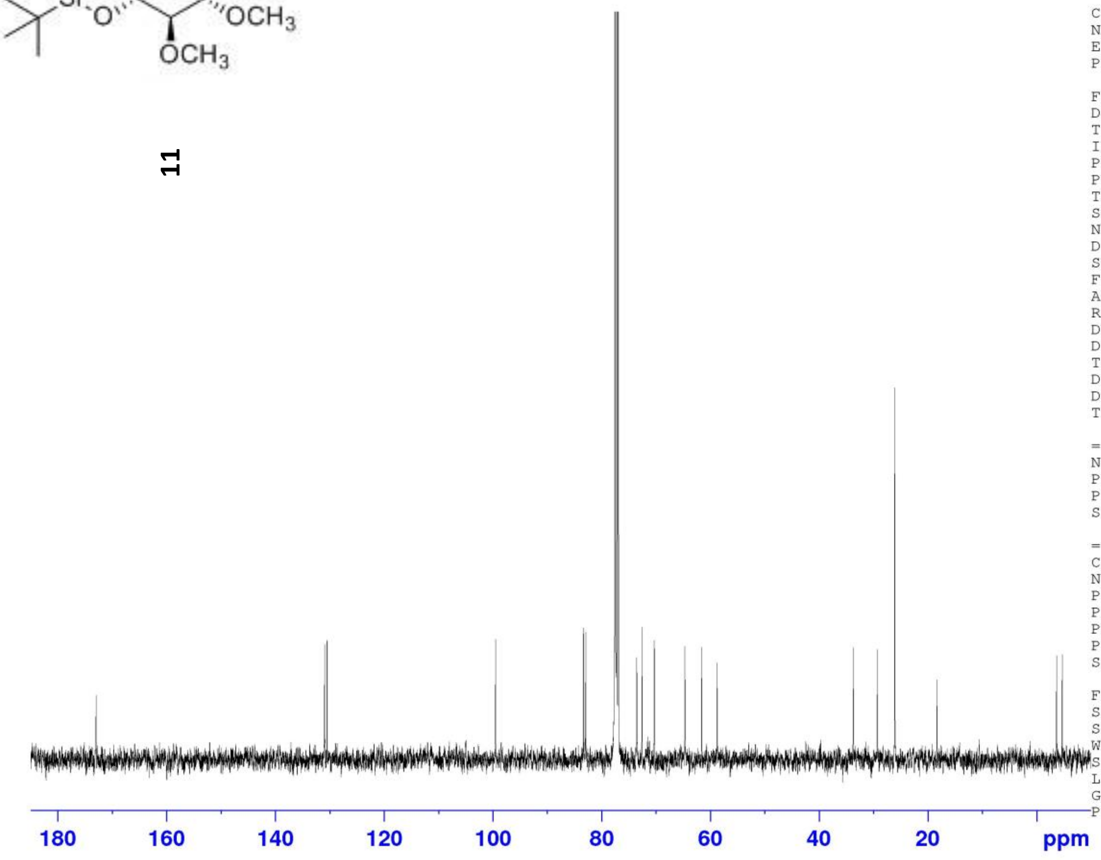


```
Current Data Parameters
NAME          rich-am-93
EXPNO                  2
PROCNO                 1

F2 - Acquisition Parameters
Date_           20130829
Time               19.00
INSTRUM            spect
PROBHD   5 mm PABBO BB/
PULPROG          zgpg30
TD                 43698
SOLVENT            CDCl3
NS                   444
DS                     4
SWH            24038.461 Hz
FIDRES          0.550104 Hz
AQ             0.9089184 sec
RG                   203
DW                20.800 usec
DE                  6.50 usec
TE                 300.1 K
D1            1.50000000 sec
D11           0.03000000 sec
TD0                    1

======== CHANNEL f1 ========
NUC1                 13C
P1                 10.00 usec
PLW1         50.00299835 W
SFO1         100.6263500 MHz

======== CHANNEL f2 ========
CPDPRG[2         waltz16
NUC2                  1H
PCPD2              90.00 usec
PLW2         12.00000000 W
PLW12         0.33333001 W
PLW13         0.27000001 W
SFO2         400.1456006 MHz

F2 - Processing parameters
SI                131072
SF           100.6162677 MHz
WDW                   EM
SSB      0
LB                  2.50 Hz
GB       0
PC                  1.40
```

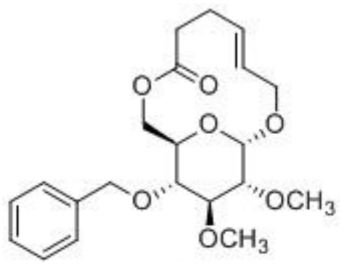

12

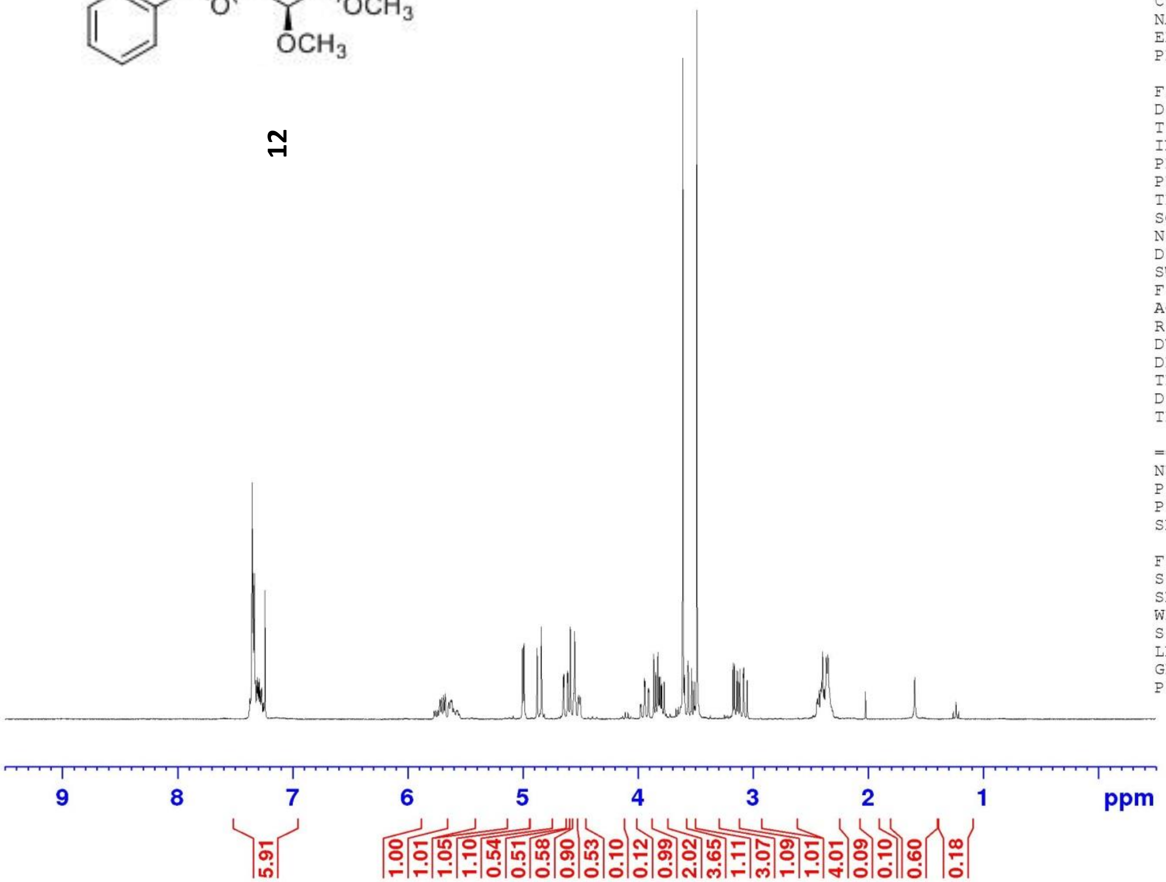

BRUKER

```
Current Data Parameters
NAME          rtd-iii-19-p3
EXPNO                     1
PROCNO                    1

F2 - Acquisition Parameters
Date_              20110811
Time                  16.22
INSTRUM               spect
PROBHD   5 mm Multinucl
PULPROG                zg30
TD                    32768
SOLVENT               CDCl3
NS                       16
DS                        2
SWH                4789.272 Hz
FIDRES             0.146157 Hz
AQ                3.4209793 sec
RG                        4
DW                  104.400 usec
DE                     6.50 usec
TE                    271.8 K
D1               1.50000000 sec
TD0                       1

======== CHANNEL f1 ========
NUC1                     1H
P1                     8.20 usec
PL1           0 dB
SFO1            300.1315032 MHz

F2 - Processing parameters
SI                    32768
SF              300.1310155 MHz
WDW                      EM
SSB           0
LB                     0.30 Hz
GB            0
PC                     1.00
```

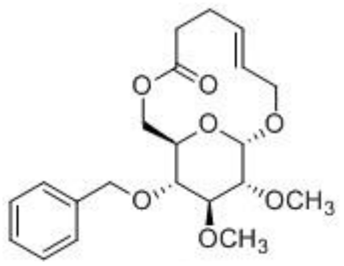

12

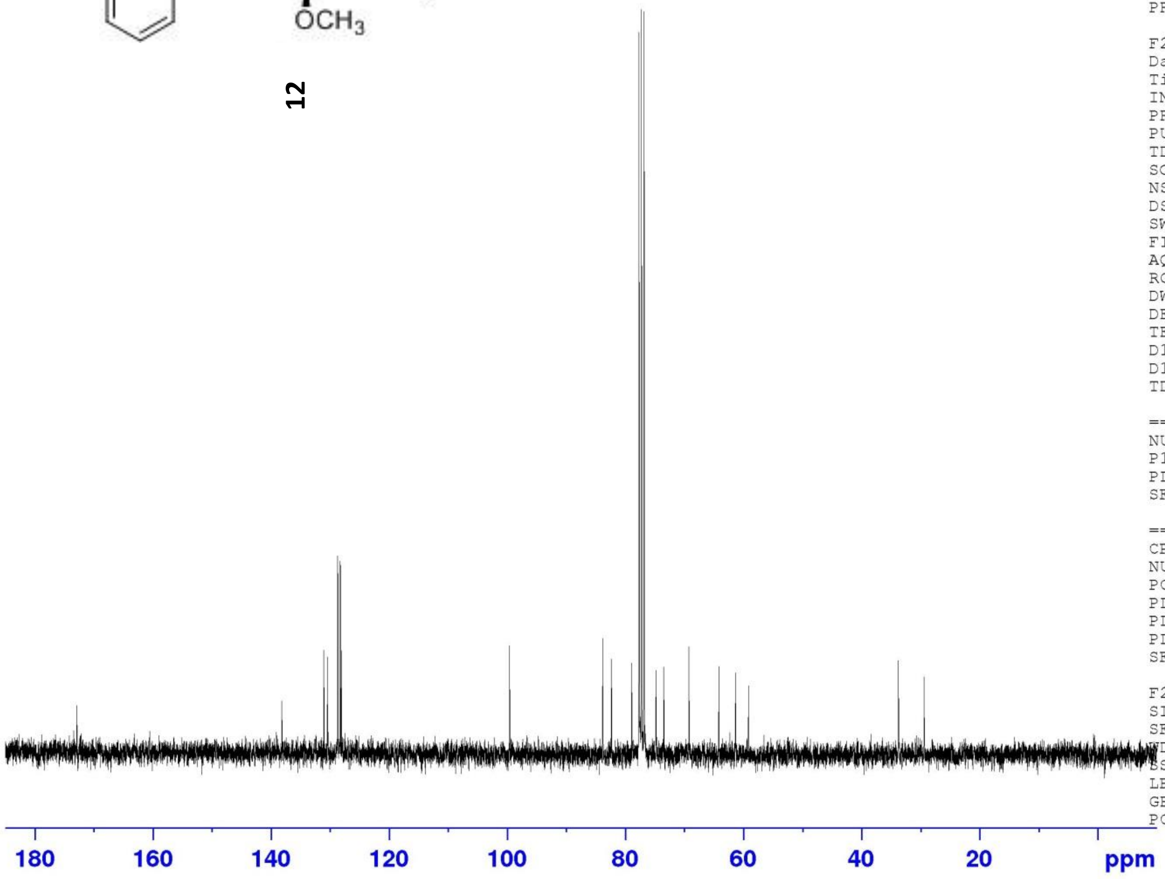


```
Current Data Parameters
NAME       rtd-iii-19-p3
EXPNO                  2
PROCNO                 1

F2 - Acquisition Parameters
Date_           20110811
Time               16.27
INSTRUM            spect
PROBHD   5 mm Multinucl
PULPROG          zgpg30
TD                 65536
SOLVENT            CDCl3
NS                   191
DS                     4
SWH           17985.611 Hz
FIDRES         0.274439 Hz
AQ            1.8219008 sec
RG                 32768
DW                27.800 usec
DE                  6.50 usec
TE                 274.4 K
D1            2.00000000 sec
D11           0.03000000 sec
TD0                    1

======== CHANNEL f1 ========
NUC1                 13C
P1                  7.50 usec
PL1       0 dB
SFO1          75.4752953 MHz

======== CHANNEL f2 ========
CPDPRG[2         waltz16
NUC2                  1H
PCPD2             100.00 usec
PL2                -2.00 dB
PL12               15.00 dB
PL13               15.00 dB
SFO2         300.1312005 MHz

F2 - Processing parameters
SI                 32768
SF            75.4679856 MHz
WDW                   EM
SSB       0
LB                  1.00 Hz
GB        0
PC                  1.40
```

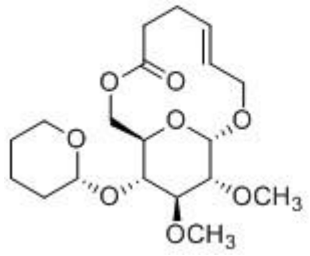

13

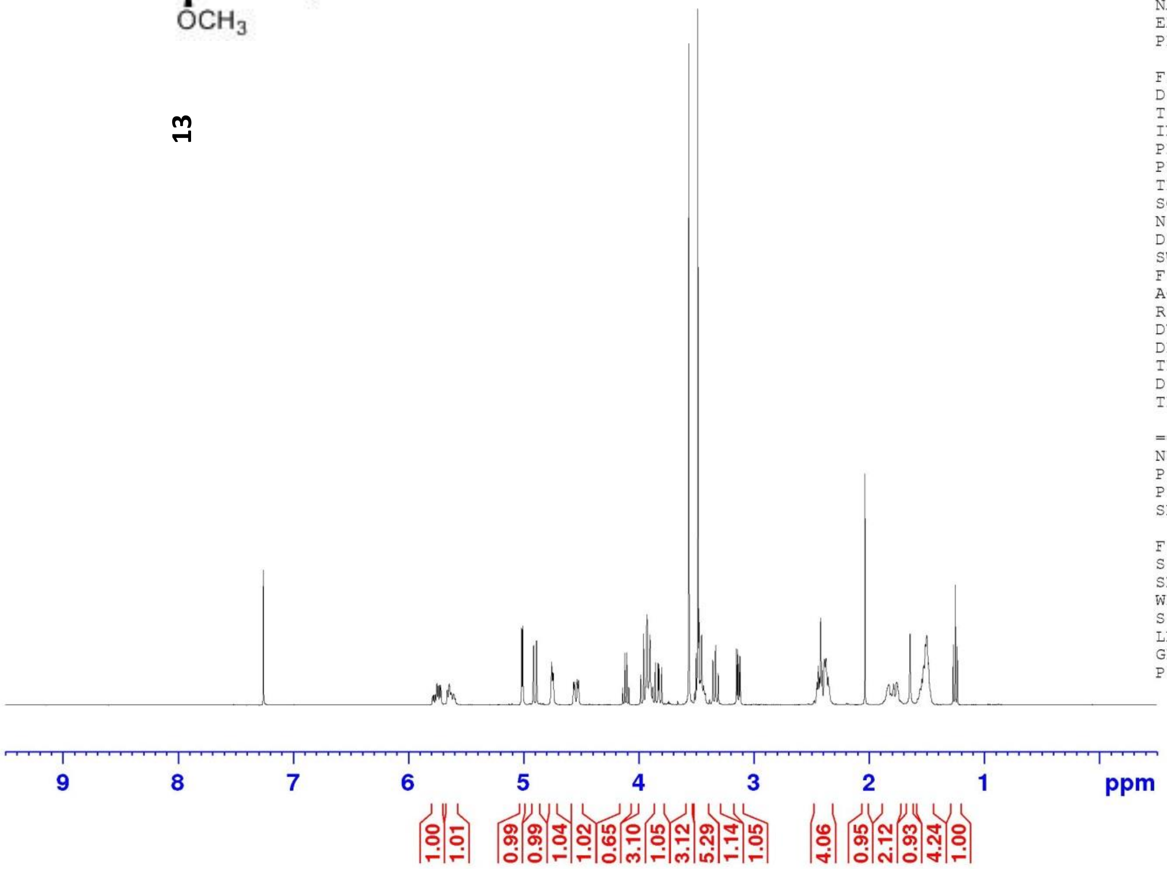

BRUKER

```
Current Data Parameters
NAME      am-115-col1-f8
EXPNO                  1
PROCNO                 1

F2 - Acquisition Parameters
Date_           20130917
Time               15.12
INSTRUM            spect
PROBHD   5 mm PABBO BB/
PULPROG             zg30
TD                 48076
SOLVENT            CDCl3
NS                    16
DS                     2
SWH             8012.820 Hz
FIDRES          0.166670 Hz
AQ             2.9999423 sec
RG                  80.6
DW                62.400 usec
DE                  6.50 usec
TE                 300.0 K
D1            2.00000000 sec
TD0                    1

======== CHANNEL f1 ========
NUC1                  1H
P1                 15.20 usec
PLW1         12.00000000 W
SFO1         400.1464009 MHz

F2 - Processing parameters
SI                131072
SF           400.1440094 MHz
WDW                   EM
SSB      0
LB                  0.50 Hz
GB       0
PC                  1.00
```

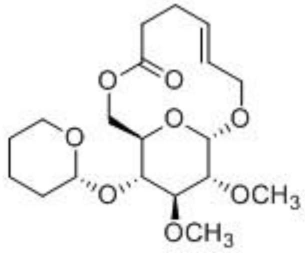

13

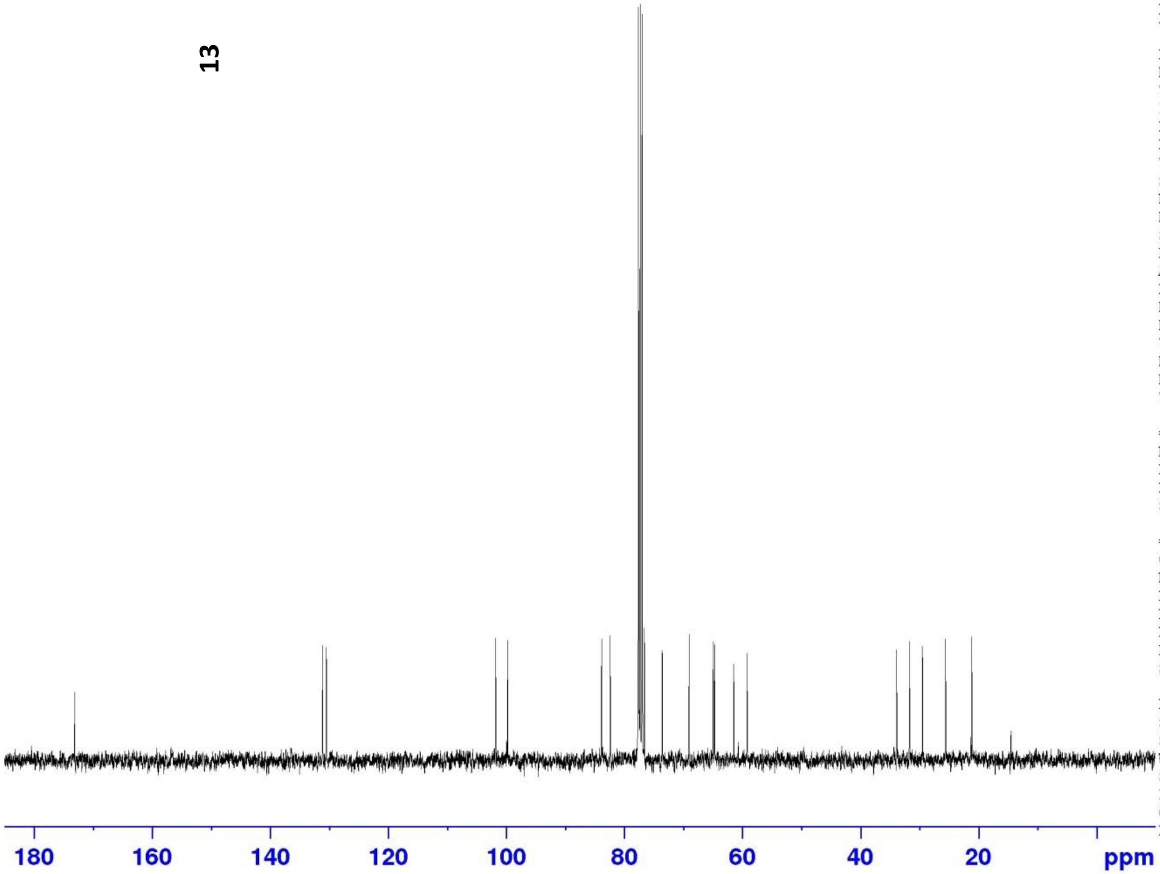

BRUKER

```
Current Data Parameters
NAME        am-115-col1-f8
EXPNO                    2
PROCNO                   1

F2 - Acquisition Parameters
Date_             20130917
Time                 15.15
INSTRUM              spect
PROBHD   5 mm PABBO BB/
PULPROG            zgpg30
TD                  43698
SOLVENT             CDCl3
NS                    157
DS                      4
SWH             24038.461 Hz
FIDRES           0.550104 Hz
AQ              0.9089184 sec
RG                    203
DW                 20.800 usec
DE                   6.50 usec
TE                  300.1 K
D1             1.50000000 sec
D11            0.03000000 sec
TD0                     1

======== CHANNEL f1 ========
NUC1                  13C
P1                  10.00 usec
PLW1          50.00299835 W
SFO1          100.6263500 MHz

======== CHANNEL f2 ========
CPDPRG[2          waltz16
NUC2                   1H
PCPD2               90.00 usec
PLW2          12.00000000 W
PLW12          0.33333001 W
PLW13          0.27000001 W
SFO2          400.1456006 MHz

F2 - Processing parameters
SI                 131072
SF            100.6162656 MHz
WDW                    EM
SSB      0
LB                   2.50 Hz
GB       0
PC                   1.40
```

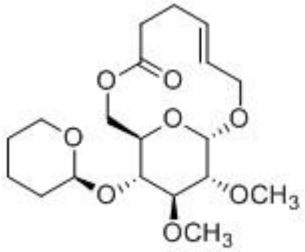

14

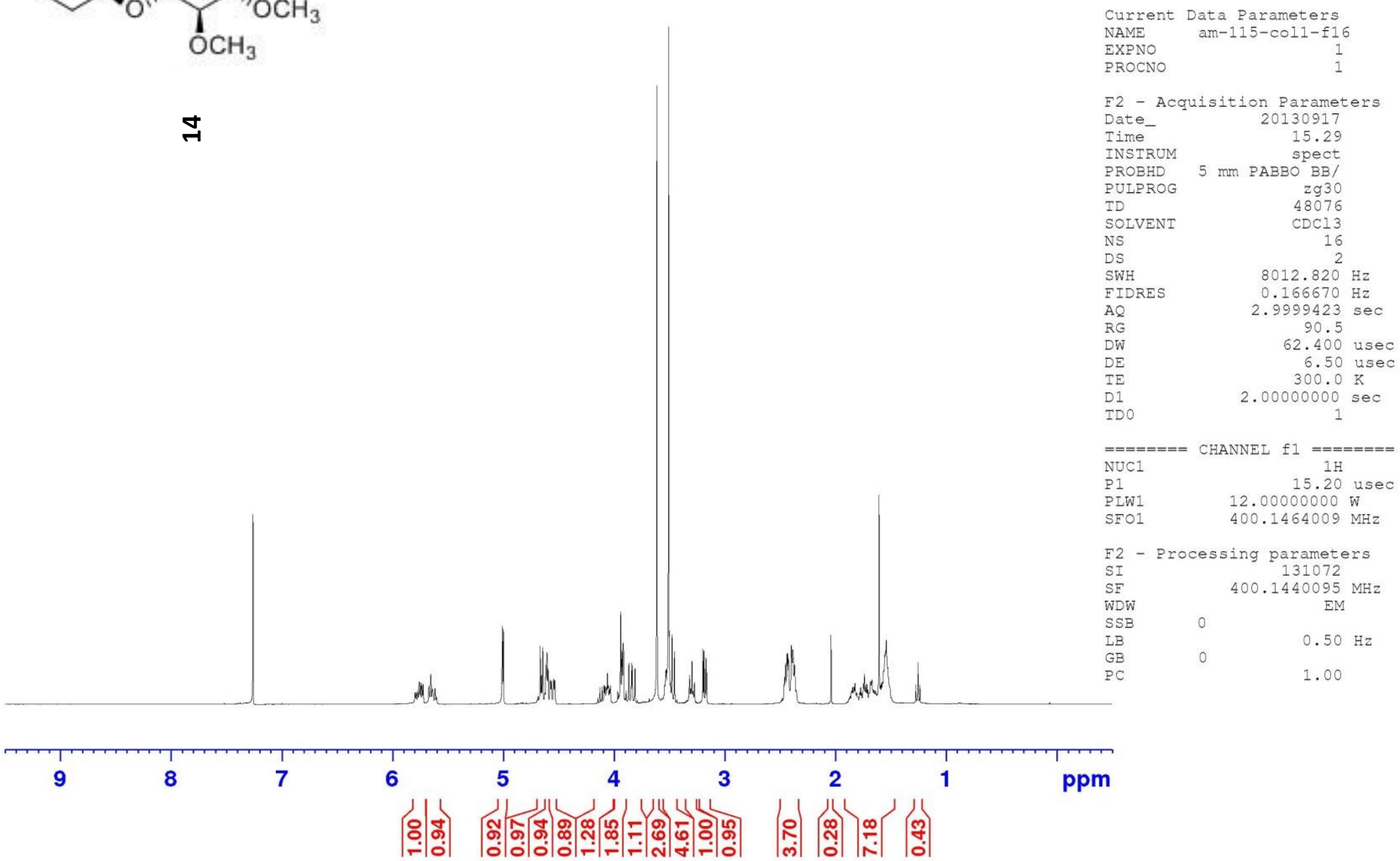
9 8 7 6 5 4 3 2 1 ppm
1.00 0.94 0.92 0.97 0.94 0.89 1.28 1.85 1.11 2.69 4.61 1.00 0.95 3.70 0.28 7.18 0.43
BRUKER
Current Data Parameters
NAME am-115-col1-f16
EXPNO 1
PROCNO 1
F2 - Acquisition Parameters
Date_ 20130917
Time 15.29
INSTRUM spect
PROBHD 5 mm PABBO BB/
PULPROG zg30
TD 48076
SOLVENT CDCl3
NS 16
DS 2
SWH 8012.820 Hz
FIDRES 0.166670 Hz
AQ 2.9999423 sec
RG 90.5
DW 62.400 usec
DE 6.50 usec
TE 300.0 K
D1 2.00000000 sec
TD0 1
======== CHANNEL f1 ========
NUC1 1H
P1 15.20 usec
PLW1 12.00000000 W
SFO1 400.1464009 MHz
F2 - Processing parameters
SI 131072
SF 400.1440095 MHz
WDW EM
SSB 0
LB 0.50 Hz
GB 0
PC 1.00

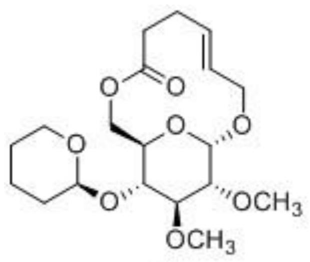

14

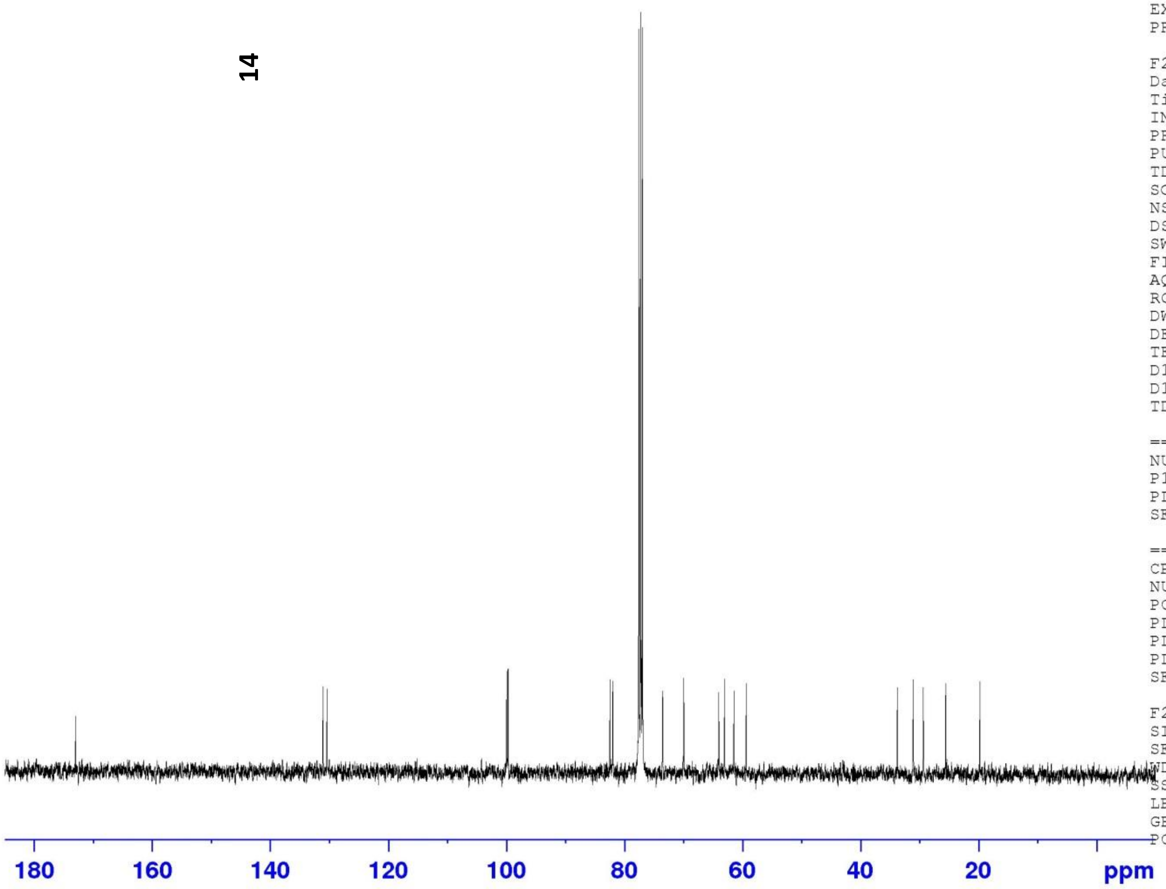


```
Current Data Parameters
NAME       am-115-col1-f16
EXPNO                    2
PROCNO                   1

F2 - Acquisition Parameters
Date_            20130917
Time                15.35
INSTRUM             spect
PROBHD    5 mm PABBO BB/
PULPROG           zgpg30
TD                 43698
SOLVENT            CDCl3
NS                   235
DS                     4
SWH            24038.461 Hz
FIDRES          0.550104 Hz
AQ             0.9089184 sec
RG                   203
DW                20.800 usec
DE                  6.50 usec
TE                 300.0 K
D1            1.50000000 sec
D11           0.03000000 sec
TD0                    1

======== CHANNEL f1 ========
NUC1                 13C
P1                 10.00 usec
PLW1         50.00299835 W
SFO1         100.6263500 MHz

======== CHANNEL f2 ========
CPDPRG[2         waltz16
NUC2                  1H
PCPD2              90.00 usec
PLW2         12.00000000 W
PLW12         0.33333001 W
PLW13         0.27000001 W
SFO2         400.1456006 MHz

F2 - Processing parameters
SI                131072
SF           100.6162656 MHz
WDW                   EM
SSB      0
LB                  2.50 Hz
GB       0
PC                  1.40
```

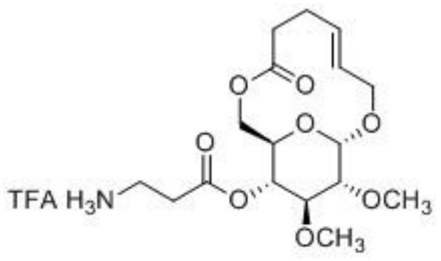

15

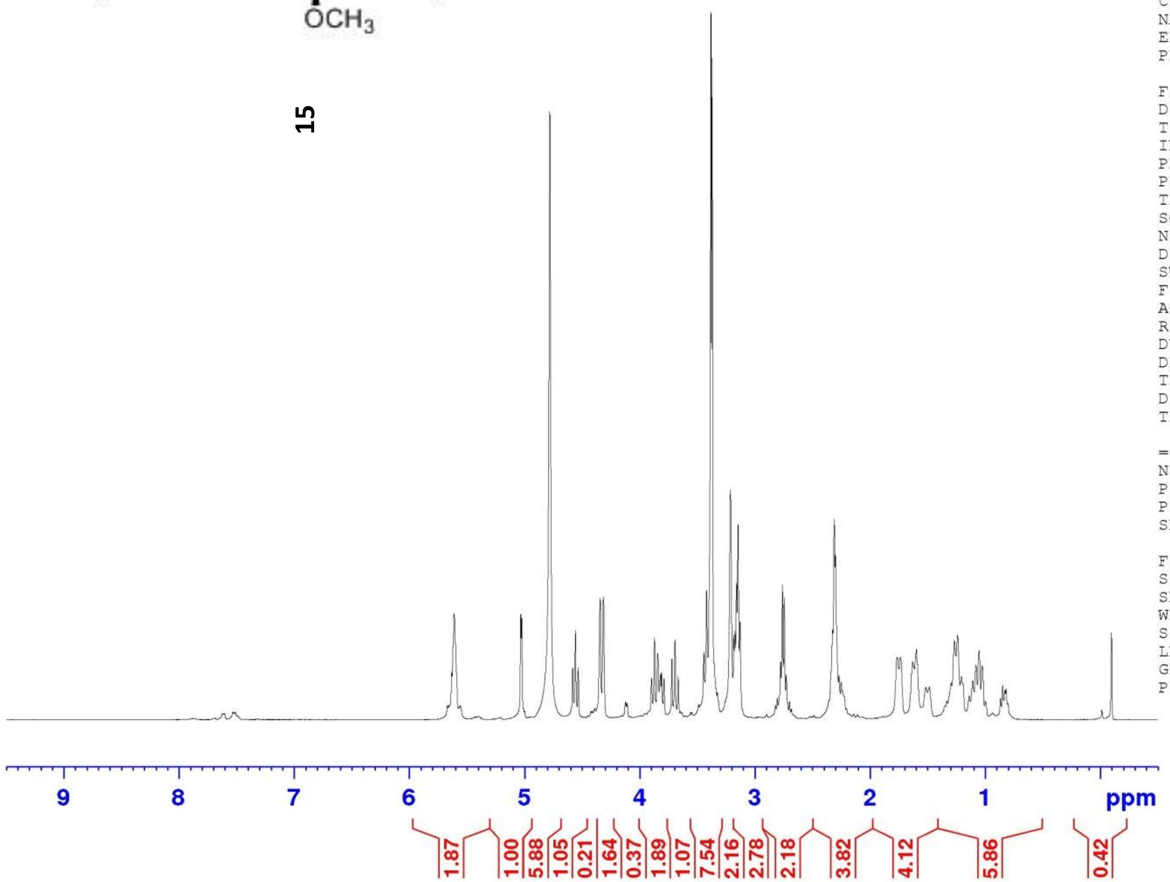


```
Current Data Parameters
NAME        rtd-iii-95-Meod
EXPNO                     1
PROCNO                    1

F2 - Acquisition Parameters
Date_              20130207
Time                  19.30
INSTRUM               spect
PROBHD     5 mm PABBO BB/
PULPROG                zg30
TD                    48076
SOLVENT                MeOD
NS                       32
DS                        2
SWH            8012.820 Hz
FIDRES         0.166670 Hz
AQ            2.9999423 sec
RG                     80.6
DW               62.400 usec
DE                 6.50 usec
TE                298.0 K
D1           2.00000000 sec
TD0                       1

======== CHANNEL f1 ========
NUC1                     1H
P1                15.22 usec
PLW1        12.00000000 W
SFO1        400.1464009 MHz

F2 - Processing parameters
SI                   131072
SF          400.1440472 MHz
WDW                      EM
SSB      0
LB                  0.50 Hz
GB       0
PC                     1.00
```

15

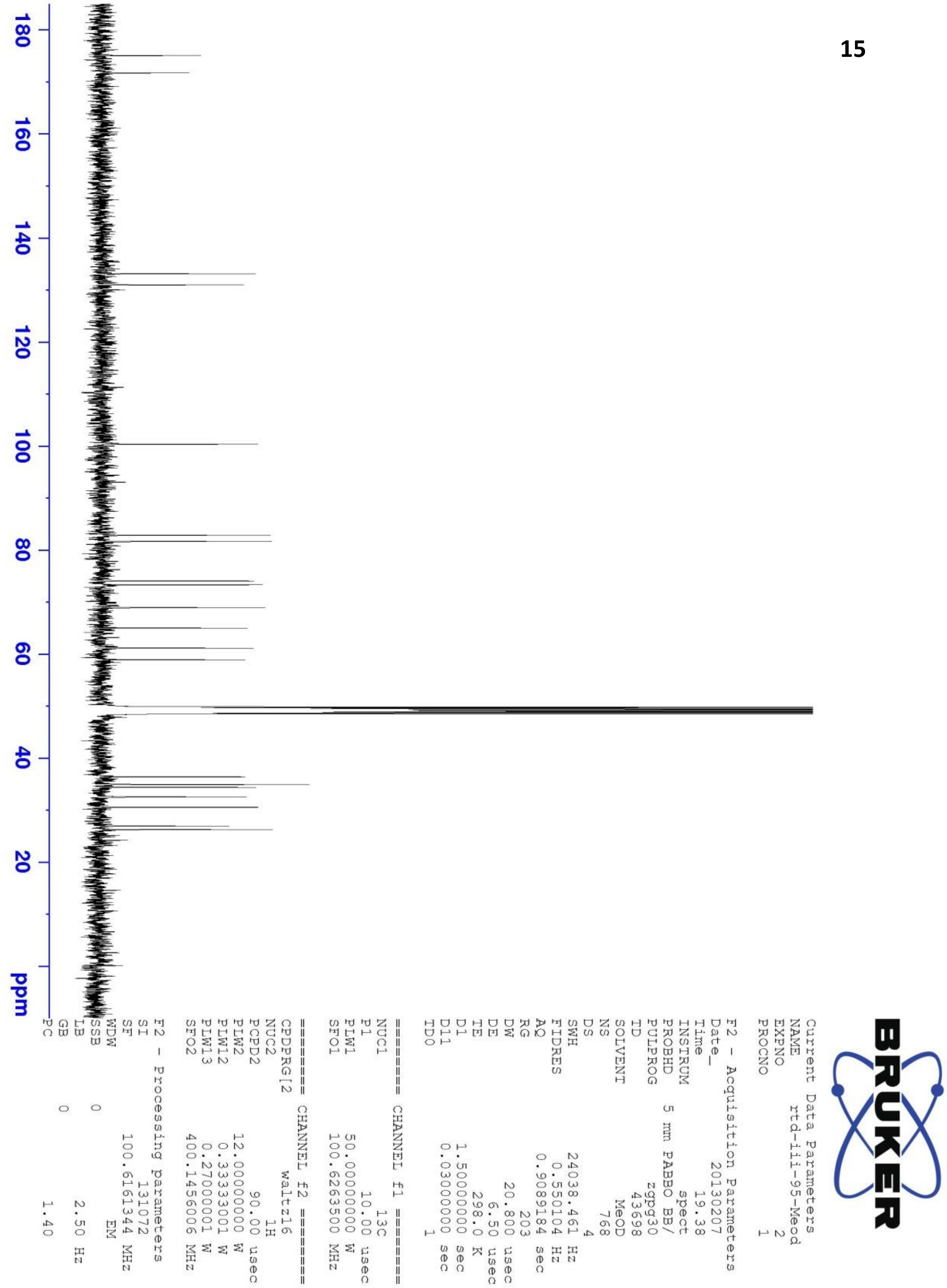

Current Data Parameters
NAME rtd-iii-95-Meod
EXPNO 2
PROCNO 1
F2 - Acquisition Parameters
Date_ 20130207
Time 19.38
INSTRUM spect
PROBHD 5 mm PABBO BB/
PULPROG zgpg30
TD 43698
SOLVENT MeOD
NS 768
DS 4
SWH 24038.461 Hz
FIDRES 0.550104 Hz
AQ 0.9089184 sec
RG 203
DW 20.800 usec
DE 6.50 usec
TE 298.0 K
D1 1.50000000 sec
D11 0.03000000 sec
TD0 1
======== CHANNEL f1 ========
NUC1 13C
P1 10.00 usec
PLW1 50.00000000 W
SFO1 100.6263500 MHz
======== CHANNEL f2 ========
CPDPRG[2 waltz16
NUC2 1H
PCPD2 90.00 usec
PLW2 12.00000000 W
PLW12 0.33333001 W
PLW13 0.27000001 W
SFO2 400.1456006 MHz
F2 - Processing parameters
SI 131072
SF 100.6161344 MHz
WDW EM
SSB 0
LB 2.50 Hz
GB 0
PC 1.40
180
160
140
120
100
80
60
40
20
ppm

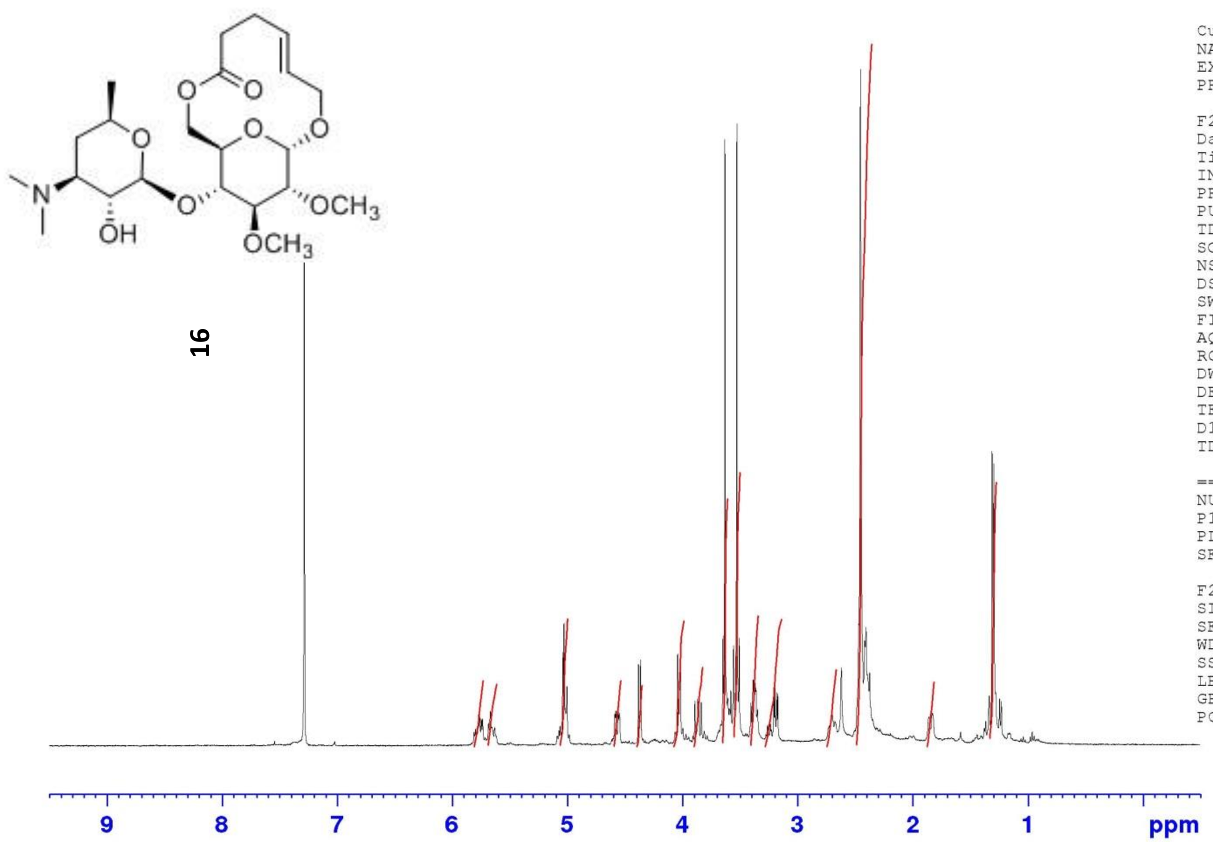

16

```
Current Data Parameters
NAME            cl-2-154a
EXPNO                   1
PROCNO                  1

F2 - Acquisition Parameters
Date_            20130819
Time                12.57
INSTRUM             spect
PROBHD   5 mm PABBO BB/
PULPROG              zg30
TD                  48076
SOLVENT             CDCl3
NS                     16
DS                      2
SWH              8012.820 Hz
FIDRES           0.166670 Hz
AQ              2.9999423 sec
RG                    203
DW                 62.400 usec
DE                   6.50 usec
TE                  300.0 K
D1             2.00000000 sec
TD0                     1

======== CHANNEL f1 ========
NUC1                   1H
P1                  15.20 usec
PLW1          12.00000000 W
SFO1          400.1464009 MHz

F2 - Processing parameters
SI                 131072
SF            400.1440000 MHz
WDW                    EM
SSB      0
LB                   0.50 Hz
GB       0
PC                   1.00
```

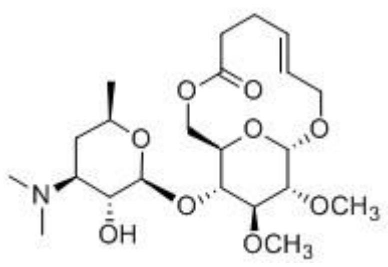

16

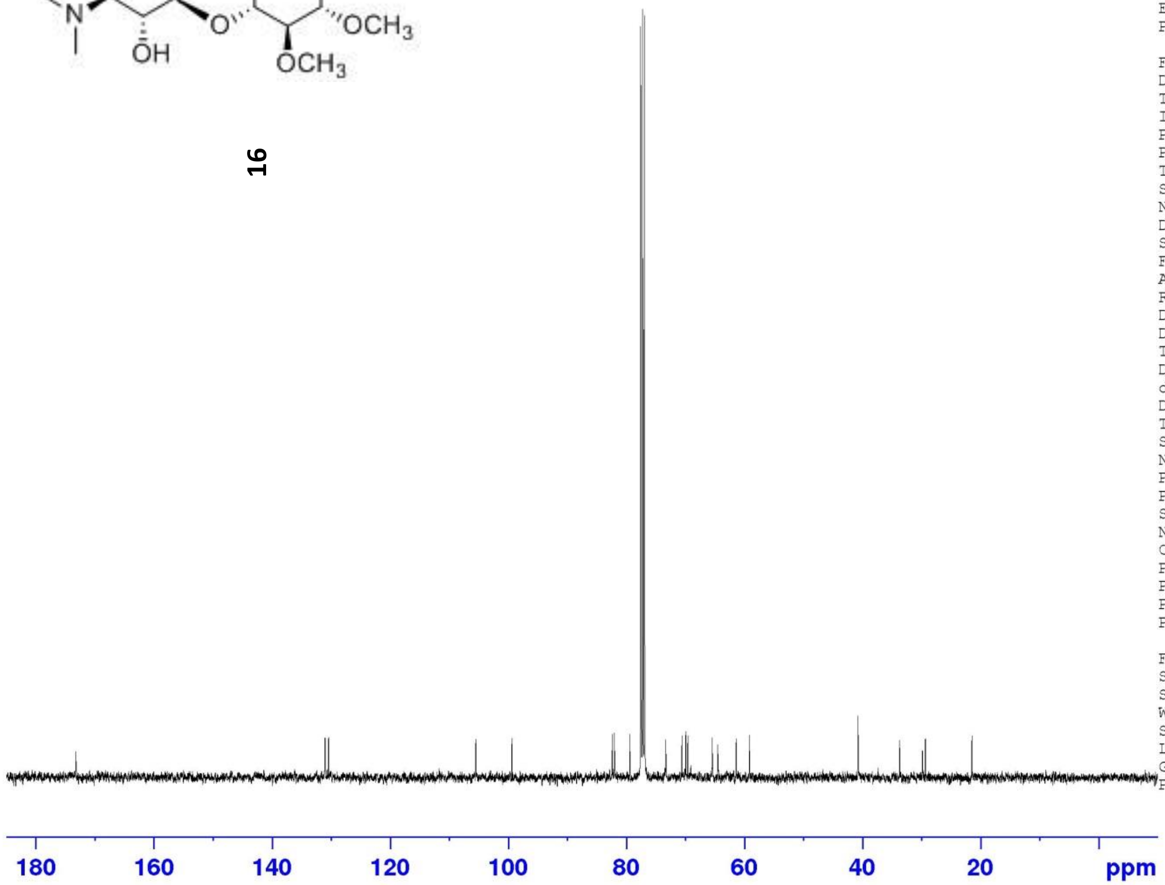


```
Current Data Parameters
NAME          cl-2-154mid
EXPNO                   2
PROCNO                  1

F2 - Acquisition Parameters
Date_            20130429
Time                15.48
INSTRUM             spect
PROBHD   5 mm PABBO BB/
PULPROG           zgpg30
TD                 43698
SOLVENT            CDCl3
NS                   253
DS                     4
SWH            24038.461 Hz
FIDRES          0.550104 Hz
AQ             0.9089184 sec
RG                   114
DW                20.800 usec
DE                  6.50 usec
TE                 300.0 K
D1            1.50000000 sec
d11           0.03000000 sec
DELTA         1.39999998 sec
TD0                    1
SFO1         100.6263500 MHz
NUC1                 13C
P1                 10.00 usec
PLW1         50.00299835 W
SFO2         400.1456006 MHz
NUC2                  1H
CPDPRG[2         waltz16
PCPD2              90.00 usec
PLW2         12.00000000 W
PLW12         0.33333001 W
PLW13         0.27000001 W

F2 - Processing parameters
SI                131072
SF           100.6162684 MHz
WDW                   EM
SSB      0
LB                  2.50 Hz
GB       0
PC                  1.40
```

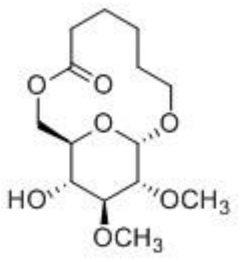

17

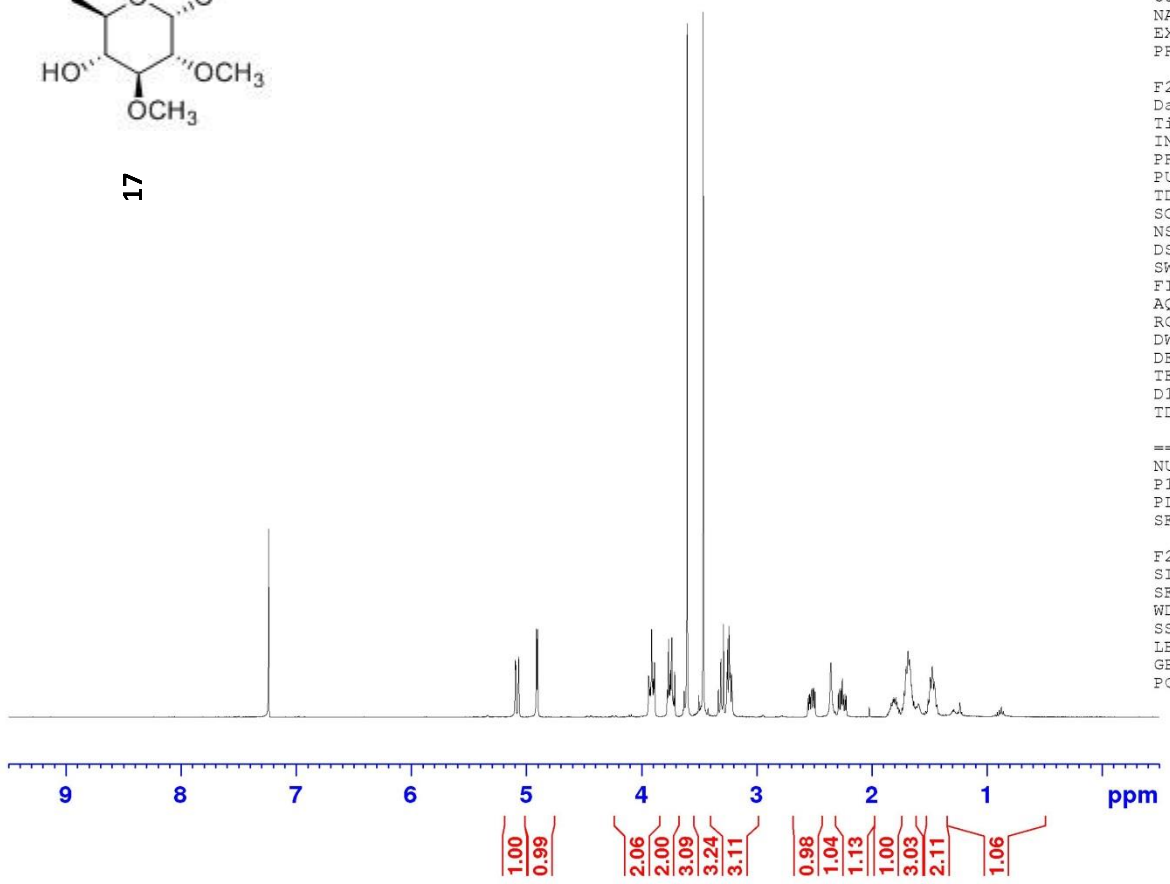


```
Current Data Parameters
NAME          rtd-iii-32=p2
EXPNO                     1
PROCNO                    1

F2 - Acquisition Parameters
Date_              20111114
Time                  19.12
INSTRUM               spect
PROBHD    5 mm PABBO BB/
PULPROG                zg30
TD                    65536
SOLVENT               CDCl3
NS                       16
DS                        2
SWH            8012.820 Hz
FIDRES         0.122266 Hz
AQ            4.0894465 sec
RG                      181
DW               62.400 usec
DE                 6.50 usec
TE                300.0 K
D1           1.00000000 sec
TD0                       1

======== CHANNEL f1 ========
NUC1                     1H
P1                 15.00 usec
PLW1         12.00000000 W
SFO1        400.1464711 MHz

F2 - Processing parameters
SI                    65536
SF          400.1440175 MHz
WDW                      EM
SSB      0
LB                 0.30 Hz
GB       0
PC                     1.00
```

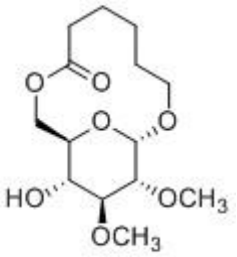

17

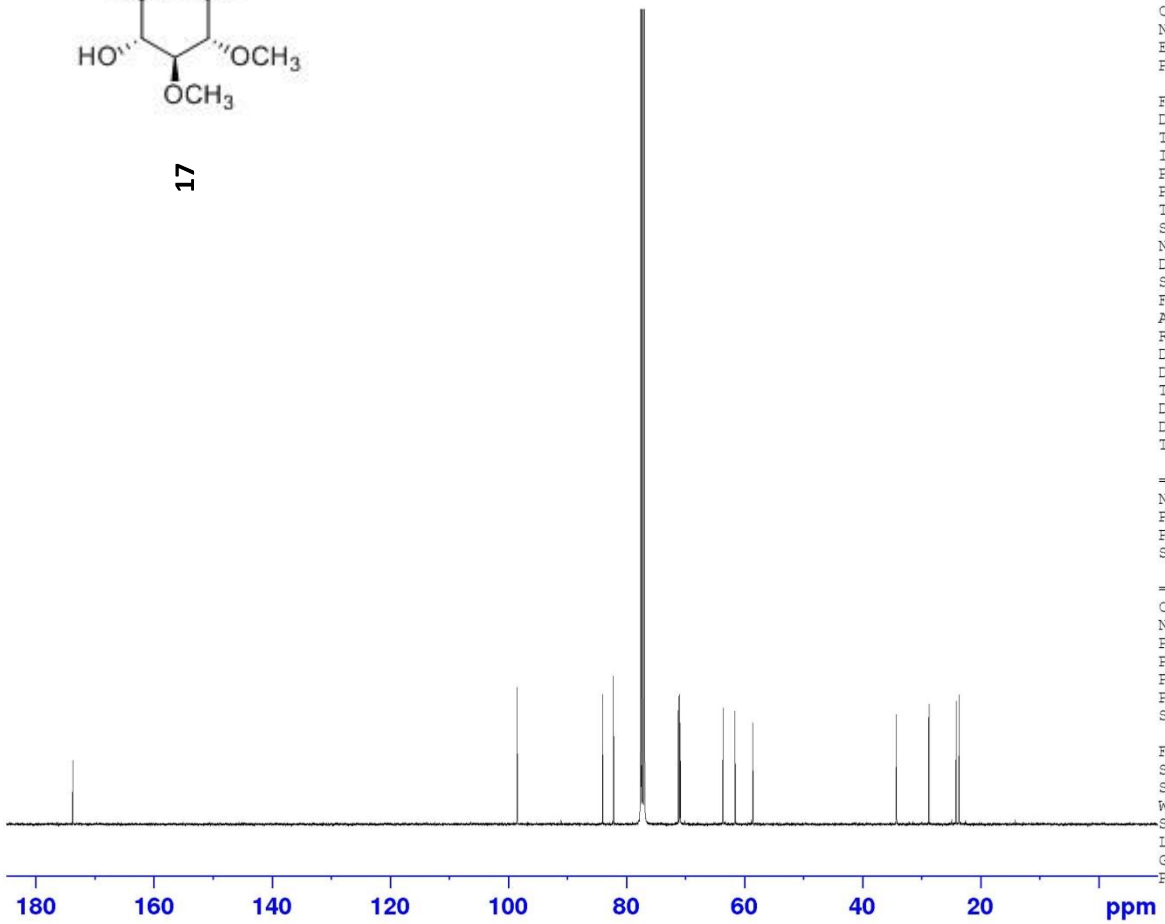


```
Current Data Parameters
NAME        rtd-iii-32=p2
EXPNO                   2
PROCNO                  1

F2 - Acquisition Parameters
Date_            20111114
Time                19.13
INSTRUM             spect
PROBHD    5 mm PABBO BB/
PULPROG           zgpg30
TD                 65536
SOLVENT            CDCl3
NS                 13673
DS                     4
SWH            24038.461 Hz
FIDRES          0.366798 Hz
AQ             1.3631488 sec
RG                   203
DW                20.800 usec
DE                  6.50 usec
TE                 300.1 K
D1            2.00000000 sec
D11           0.03000000 sec
TD0                    1

======== CHANNEL f1 ========
NUC1                 13C
P1                 10.00 usec
PLW1         50.00000000 W
SFO1         100.6263500 MHz

======== CHANNEL f2 ========
CPDPRG[2         waltz16
NUC2                  1H
PCPD2              90.00 usec
PLW2         12.00000000 W
PLW12         0.33333001 W
PLW13         0.27000001 W
SFO2         400.1456006 MHz

F2 - Processing parameters
SI                 32768
SF           100.6162656 MHz
WDW                   EM
SSB      0
LB                  1.00 Hz
GB       0
PC                  1.40
```

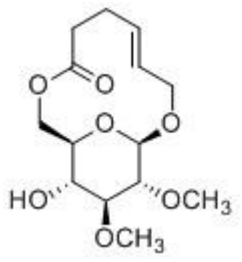

6

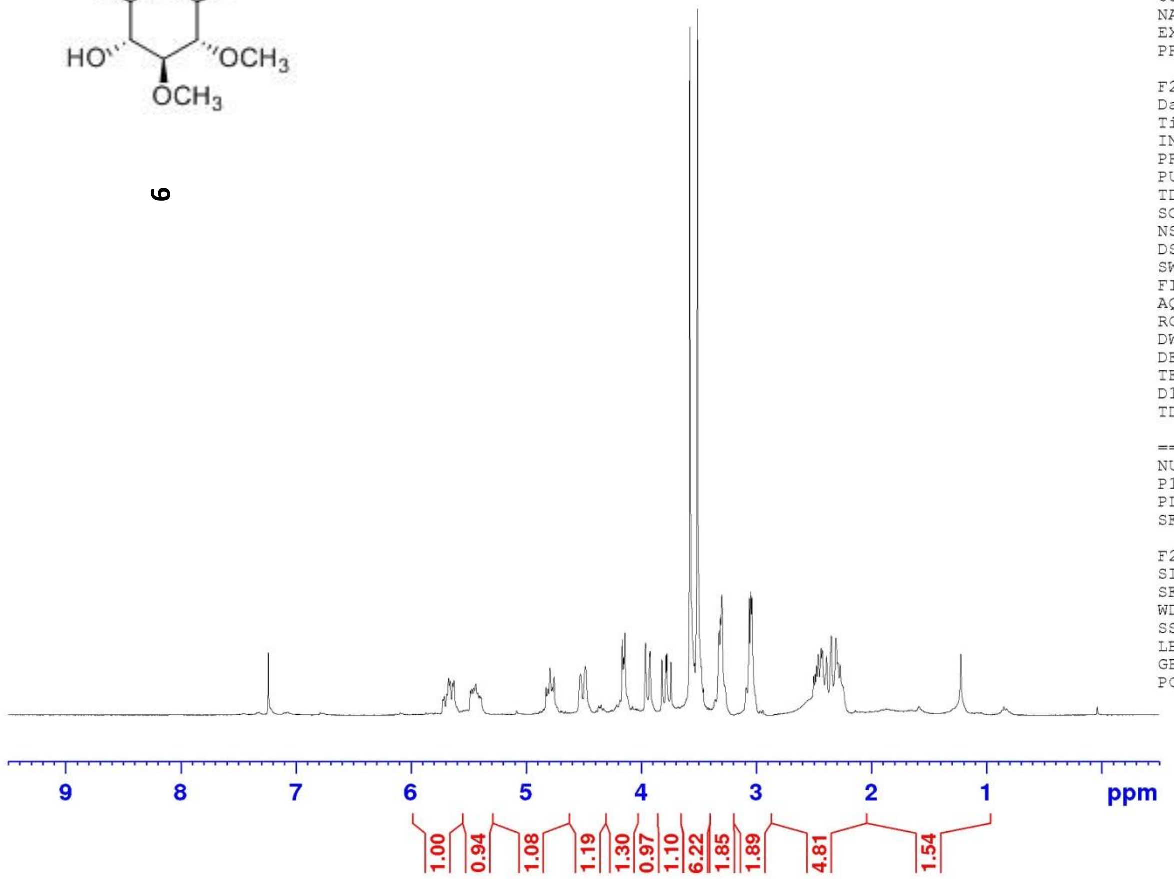


```
Current Data Parameters
NAME        rtd-ii-204-p2a
EXPNO                    1
PROCNO                   1

F2 - Acquisition Parameters
Date_             20110601
Time                 21.10
INSTRUM              spect
PROBHD   5 mm Multinucl
PULPROG               zg30
TD                   32768
SOLVENT              CDCl3
NS                     128
DS                       2
SWH               3906.250 Hz
FIDRES            0.119209 Hz
AQ               4.1943040 sec
RG                     256
DW                 128.000 usec
DE                    6.50 usec
TE                   300.2 K
D1              1.50000000 sec
TD0                      1

======== CHANNEL f1 ========
NUC1                    1H
P1                    8.20 usec
PL1          0 dB
SFO1           300.1326507 MHz

F2 - Processing parameters
SI                   32768
SF             300.1310160 MHz
WDW                     EM
SSB          0
LB                    0.30 Hz
GB           0
PC                    1.00
```

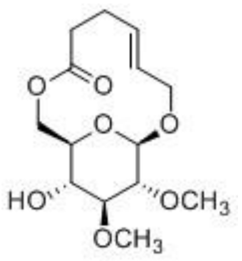

6

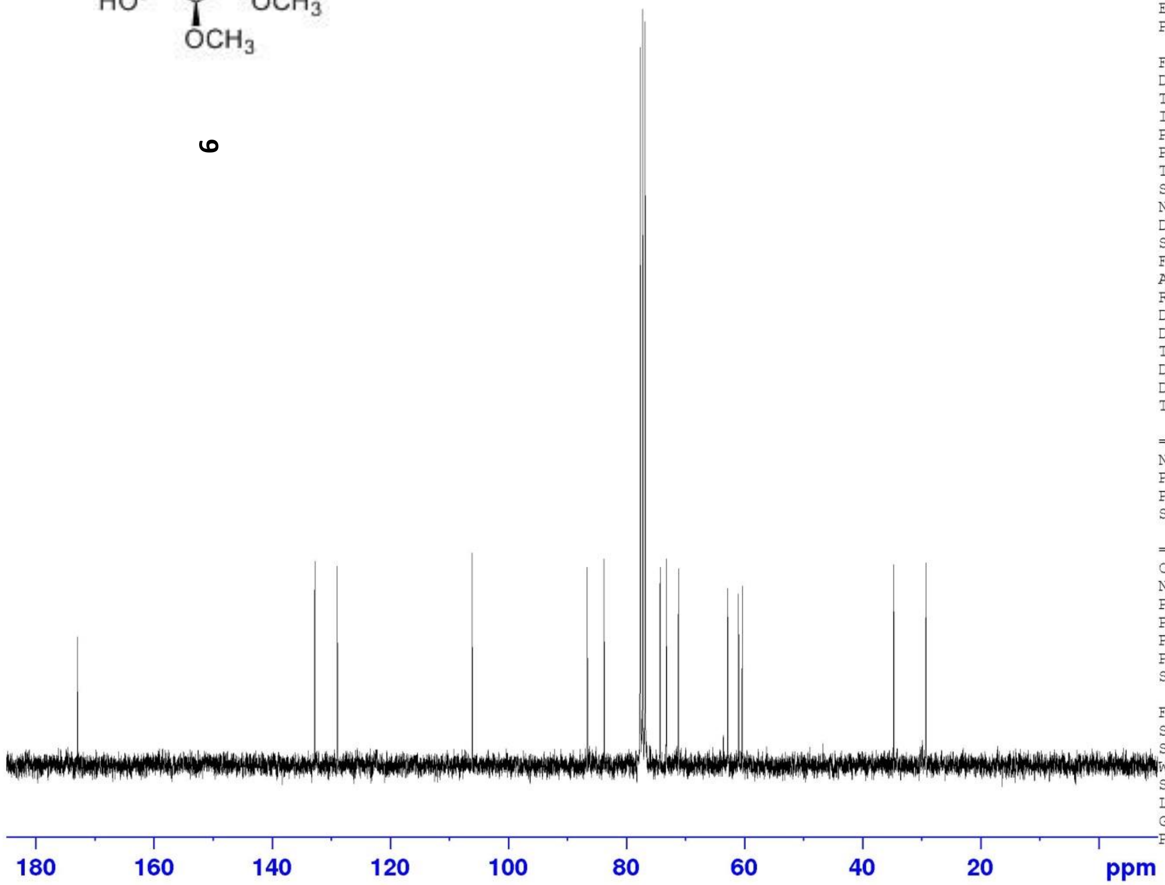


```
Current Data Parameters
NAME      rtd-ii-204-p2a
EXPNO                  2
PROCNO                 1

F2 - Acquisition Parameters
Date_           20110602
Time                2.53
INSTRUM            spect
PROBHD   5 mm Multinucl
PULPROG          zgpg30
TD                65536
SOLVENT           CDCl3
NS                 5120
DS                    4
SWH           17985.611 Hz
FIDRES         0.274439 Hz
AQ            1.8219008 sec
RG                32768
DW               27.800 usec
DE                 6.50 usec
TE                302.0 K
D1           2.00000000 sec
D11          0.03000000 sec
TD0                   1

======== CHANNEL f1 ========
NUC1                13C
P1                 7.50 usec
PL1       0 dB
SFO1        75.4752953 MHz

======== CHANNEL f2 ========
CPDPRG[2        waltz16
NUC2                 1H
PCPD2            100.00 usec
PL2               -2.00 dB
PL12              15.00 dB
PL13              15.00 dB
SFO2       300.1312005 MHz

F2 - Processing parameters
SI                32768
SF          75.4679850 MHz
WDW                  EM
SSB       0
LB                 1.00 Hz
GB        0
PC                 1.40
```

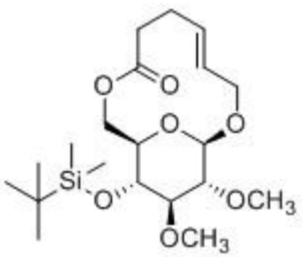

18

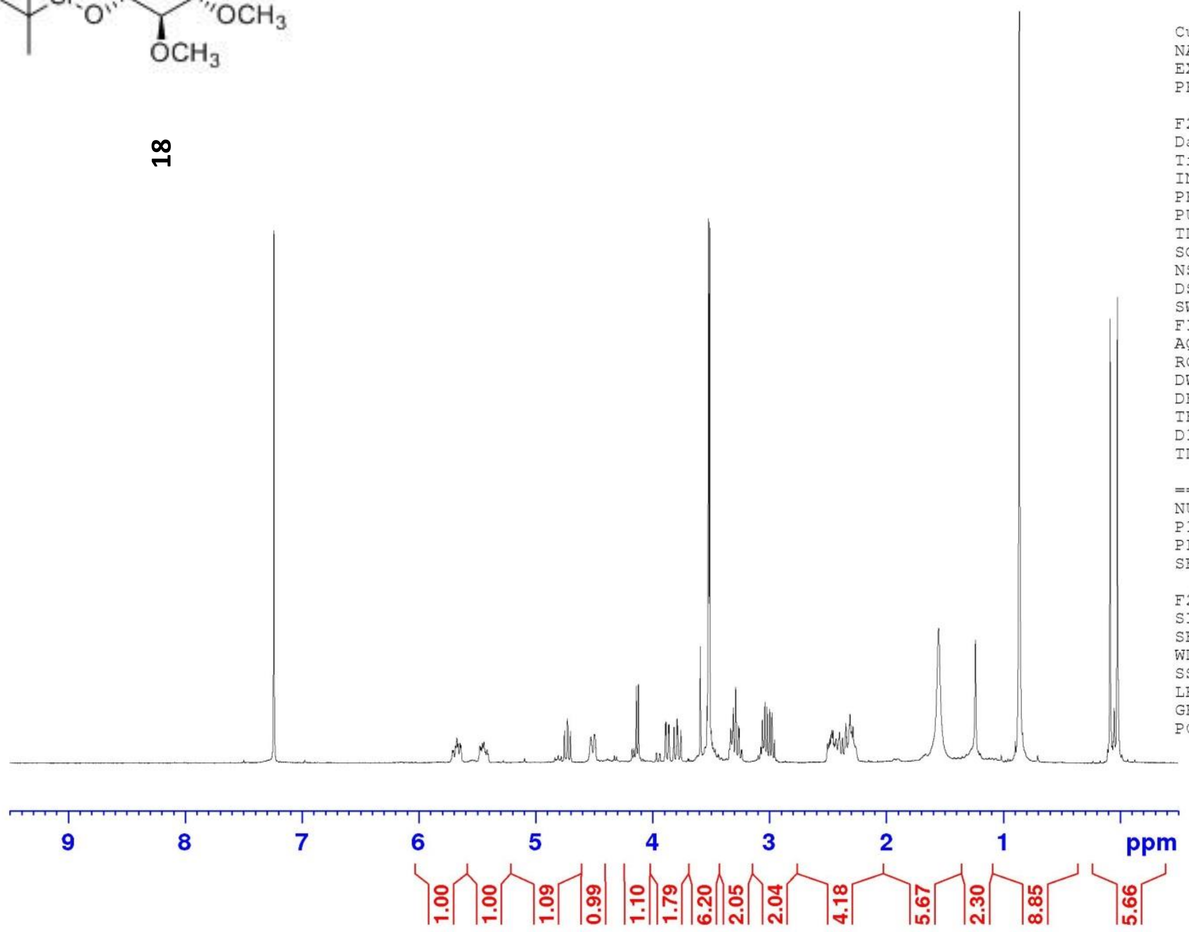


```
Current Data Parameters
NAME        rtd-iii-104-p2
EXPNO                    1
PROCNO                   1

F2 - Acquisition Parameters
Date_             20130807
Time                 12.02
INSTRUM              spect
PROBHD   5 mm PABBO BB/
PULPROG               zg30
TD                   48076
SOLVENT              CDCl3
NS                      32
DS                       2
SWH               8012.820 Hz
FIDRES            0.166670 Hz
AQ               2.9999423 sec
RG                     181
DW                  62.400 usec
DE                    6.50 usec
TE                   300.0 K
D1              2.00000000 sec
TD0                      1

======== CHANNEL f1 ========
NUC1                    1H
P1                   15.20 usec
PLW1           12.00000000 W
SFO1           400.1464009 MHz

F2 - Processing parameters
SI                  131072
SF             400.1440177 MHz
WDW                     EM
SSB      0
LB                    0.50 Hz
GB       0
PC                    1.00
```

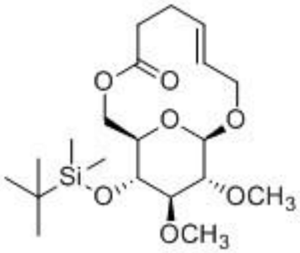

18

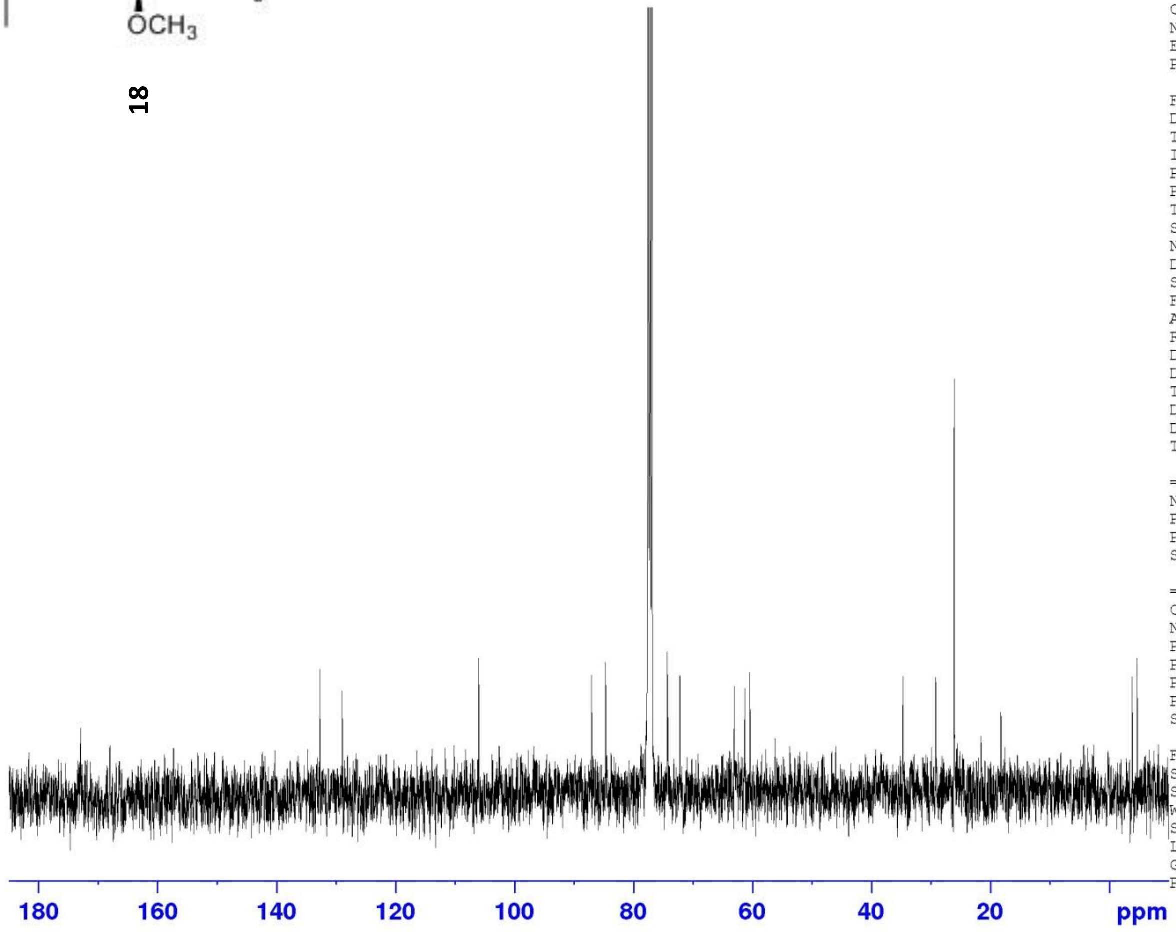


```
Current Data Parameters
NAME      rtd-iii-104-p2
EXPNO                  2
PROCNO                 1

F2 - Acquisition Parameters
Date_           20130807
Time               12.05
INSTRUM            spect
PROBHD   5 mm PABBO BB/
PULPROG          zgpg30
TD                 43698
SOLVENT            CDCl3
NS                   512
DS                     4
SWH            24038.461 Hz
FIDRES          0.550104 Hz
AQ             0.9089184 sec
RG                   114
DW                20.800 usec
DE                  6.50 usec
TE                 300.2 K
D1            1.50000000 sec
D11           0.03000000 sec
TD0                    1

======== CHANNEL f1 ========
NUC1                 13C
P1                 10.00 usec
PLW1         50.00299835 W
SFO1         100.6263500 MHz

======== CHANNEL f2 ========
CPDPRG[2         waltz16
NUC2                  1H
PCPD2              90.00 usec
PLW2         12.00000000 W
PLW12         0.33333001 W
PLW13         0.27000001 W
SFO2         400.1456006 MHz

F2 - Processing parameters
SI                131072
SF           100.6162695 MHz
WDW                   EM
SSB      0
LB                  2.50 Hz
GB       0
PC                  1.40
```

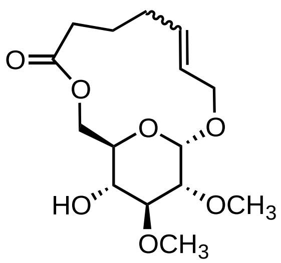
O
O
O
O
HO
OCH3
OCH3

19

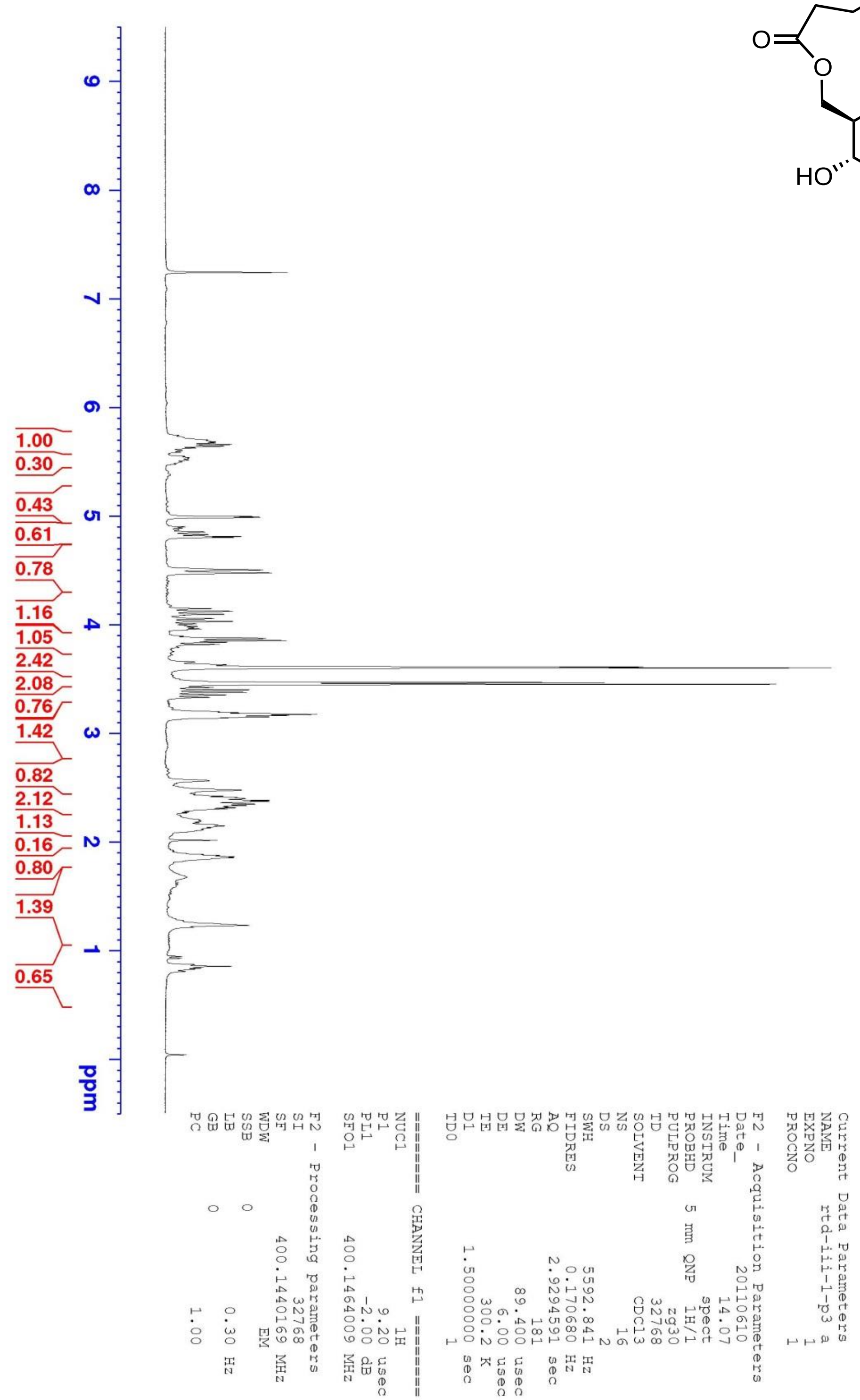
9
8
7
6
5
4
3
2
1
ppm
1.00
0.30
0.43
0.61
0.78
1.16
1.05
2.42
2.08
0.76
1.42
0.82
2.12
1.13
0.16
0.80
1.39
0.65
BRUKER
Current Data Parameters
NAME rtd-iii-1-p3 a
EXPNO 1
PROCNO 1
F2 - Acquisition Parameters
Date_ 20110610
Time 14.07
INSTRUM spect
PROBHD 5 mm QNP 1H/1
PULPROG zg30
TD 32768
SOLVENT CDCl3
NS 16
DS 2
SWH 5592.841 Hz
FIDRES 0.170680 Hz
AQ 2.9294591 sec
RG 181
DW 89.400 usec
DE 6.00 usec
TE 300.2 K
D1 1.50000000 sec
TD0 1
======== CHANNEL f1 ========
NUC1 1H
P1 9.20 usec
PL1 -2.00 dB
SFO1 400.1464009 MHz
F2 - Processing parameters
SI 32768
SF 400.1440169 MHz
WDW EM
SSB 0
LB 0.30 Hz
GB 0
PC 1.00

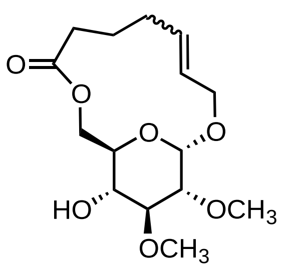
O
O
O
O
HO
OCH3
OCH3

19

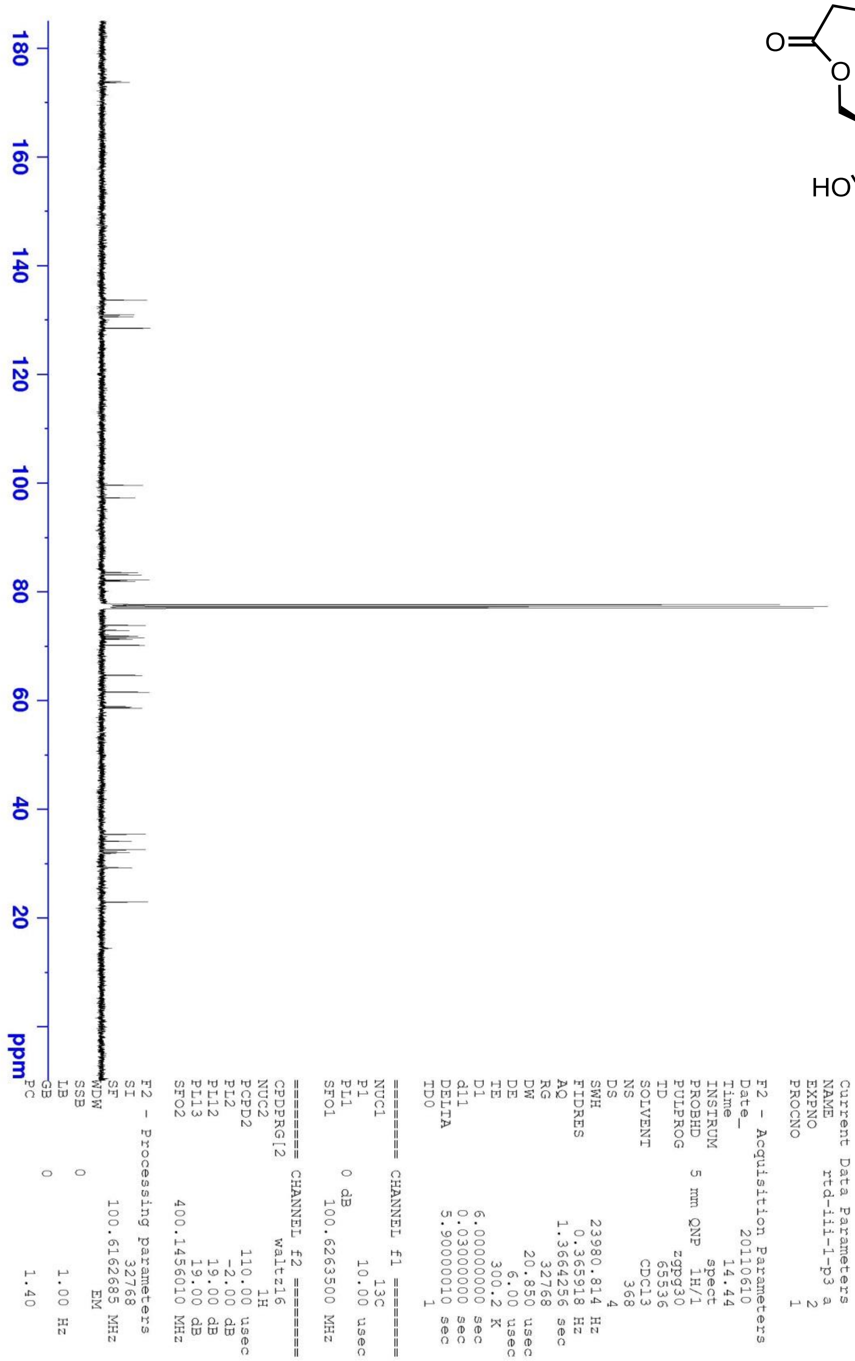
180
160
140
120
100
80
60
40
20
ppm
BRUKER
Current Data Parameters
NAME rtd-iii-1-p3 a
EXPNO 2
PROCNO 1
F2 - Acquisition Parameters
Date_ 20110610
Time 14.44
INSTRUM spect
PROBHD 5 mm QNP 1H/1
PULPROG zgpg30
TD 65536
SOLVENT CDCl3
NS 368
DS 4
SWH 23980.814 Hz
FIDRES 0.365918 Hz
AQ 1.3664256 sec
RG 32768
DW 20.850 usec
DE 6.00 usec
TE 300.2 K
D1 6.00000000 sec
d11 0.03000000 sec
DELTA 5.90000010 sec
TD0 1
======== CHANNEL f1 ========
NUC1 13C
P1 10.00 usec
PL1 0 dB
SFO1 100.6263500 MHz
======== CHANNEL f2 ========
CPDPRG[2 waltz16
NUC2 1H
PCPD2 110.00 usec
PL2 -2.00 dB
PL12 19.00 dB
PL13 19.00 dB
SFO2 400.1456010 MHz
F2 - Processing parameters
SI 32768
SF 100.6162685 MHz
WDW EM
SSB 0
LB 1.00 Hz
GB 0
PC 1.40

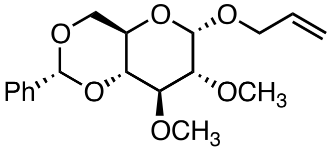
O
O
O
O
Ph
OCH3
OCH3

8a

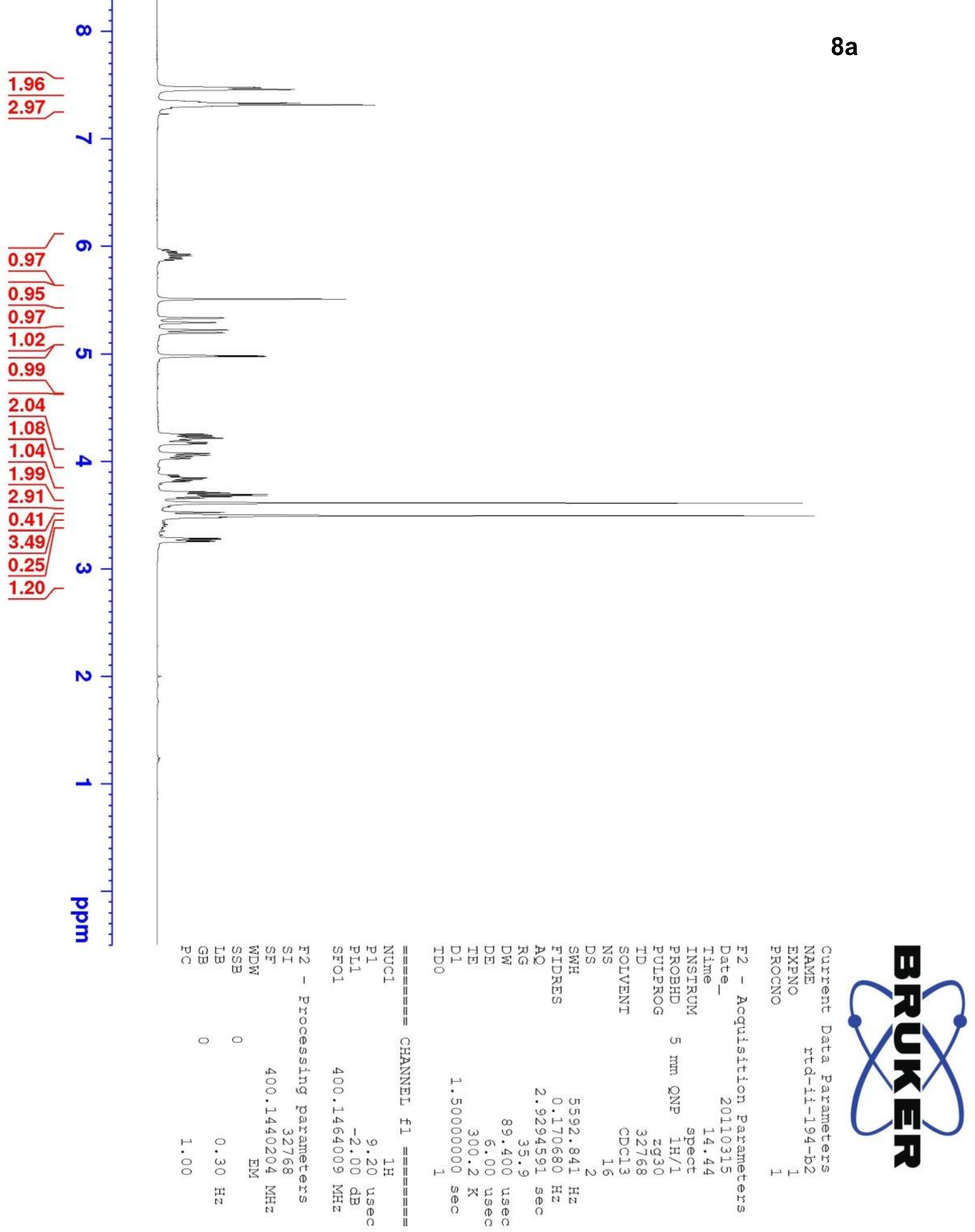
9
8
7
6
5
4
3
2
1
ppm
1.96
2.97
0.97
0.95
0.97
1.02
0.99
2.04
1.08
1.04
1.99
2.91
0.41
3.49
0.25
1.20
Current Data Parameters
NAME rtd-ii-194-b2
EXPNO 1
PROCNO 1
F2 - Acquisition Parameters
Date_ 20110315
Time 14.44
INSTRUM spect
PROBHD 5 mm QNP 1H/1
PULPROG zg30
TD 32768
SOLVENT CDCl3
NS 16
DS 2
SWH 5592.841 Hz
FIDRES 0.170680 Hz
AQ 2.9294591 sec
RG 35.9
DW 89.400 usec
DE 6.00 usec
TE 300.2 K
D1 1.5000000 sec
TD0 1
======== CHANNEL f1 ========
NUC1 1H
P1 9.20 usec
PL1 -2.00 dB
SFO1 400.1464009 MHz
F2 - Processing parameters
SI 32768
SF 400.1440204 MHz
WDW EM
SSB 0
LB 0.30 Hz
GB 0
PC 1.00

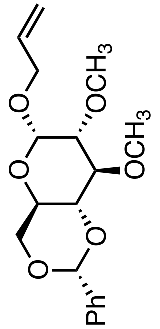

8a

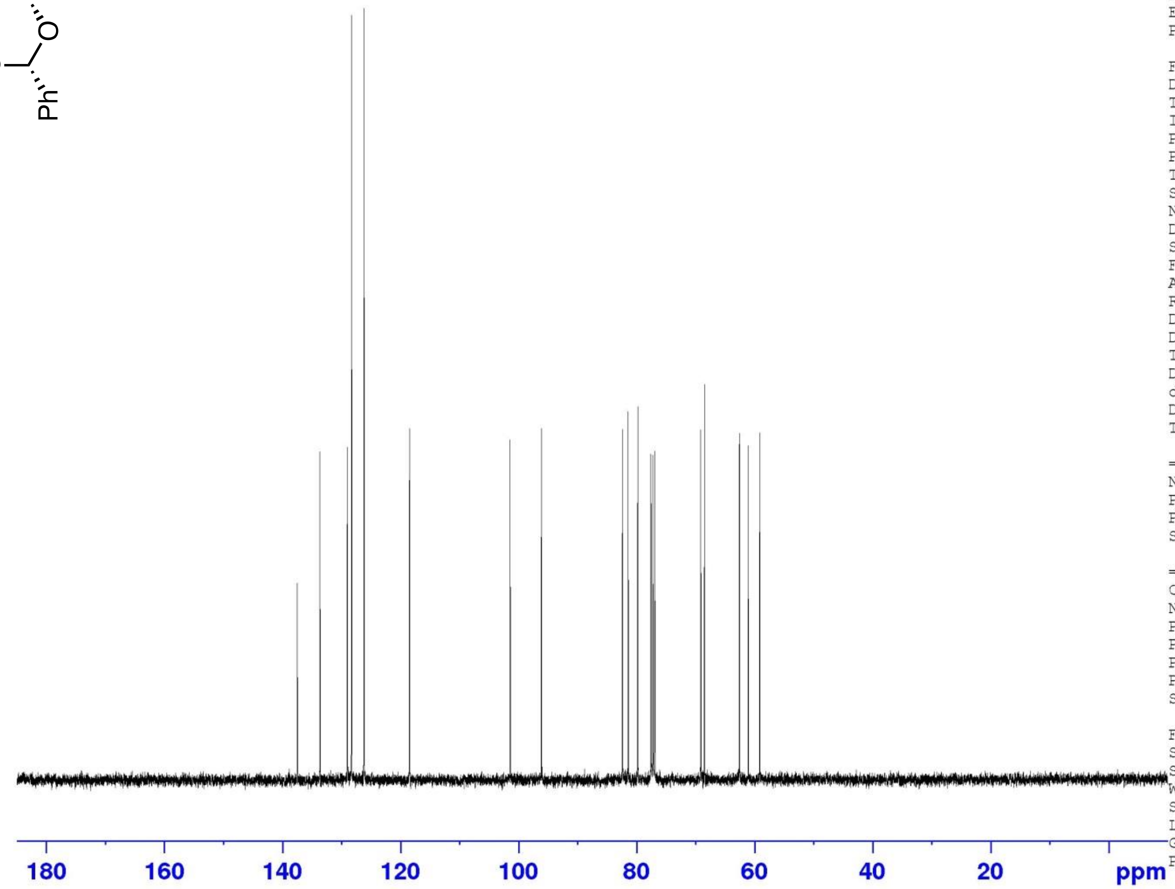

BRUKER

```
Current Data Parameters
NAME          rtd-ii-194-b2
EXPNO                     2
PROCNO                    1

F2 - Acquisition Parameters
Date_              20110315
Time                  14.49
INSTRUM               spect
PROBHD   5 mm QNP  1H/1
PULPROG             zgpg30
TD                   65536
SOLVENT              CDCl3
NS                      77
DS                       4
SWH              23980.814 Hz
FIDRES            0.365918 Hz
AQ               1.3664256 sec
RG                   32768
DW                  20.850 usec
DE                    6.00 usec
TE                   300.2 K
D1              2.00000000 sec
d11             0.03000000 sec
DELTA           1.89999998 sec
TD0                      1

======== CHANNEL f1 ========
NUC1                   13C
P1                   10.00 usec
PL1       0 dB
SFO1           100.6263500 MHz

======== CHANNEL f2 ========
CPDPRG[2           waltz16
NUC2                    1H
PCPD2               110.00 usec
PL2                  -2.00 dB
PL12                 19.00 dB
PL13                 19.00 dB
SFO2           400.1456010 MHz

F2 - Processing parameters
SI                   32768
SF             100.6162806 MHz
WDW                     EM
SSB       0
LB                    1.00 Hz
GB        0
PC                    1.40
```

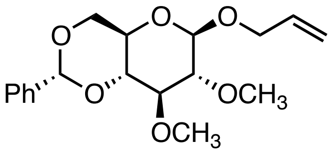
O
O
O
O
Ph
OCH3
OCH3

8b

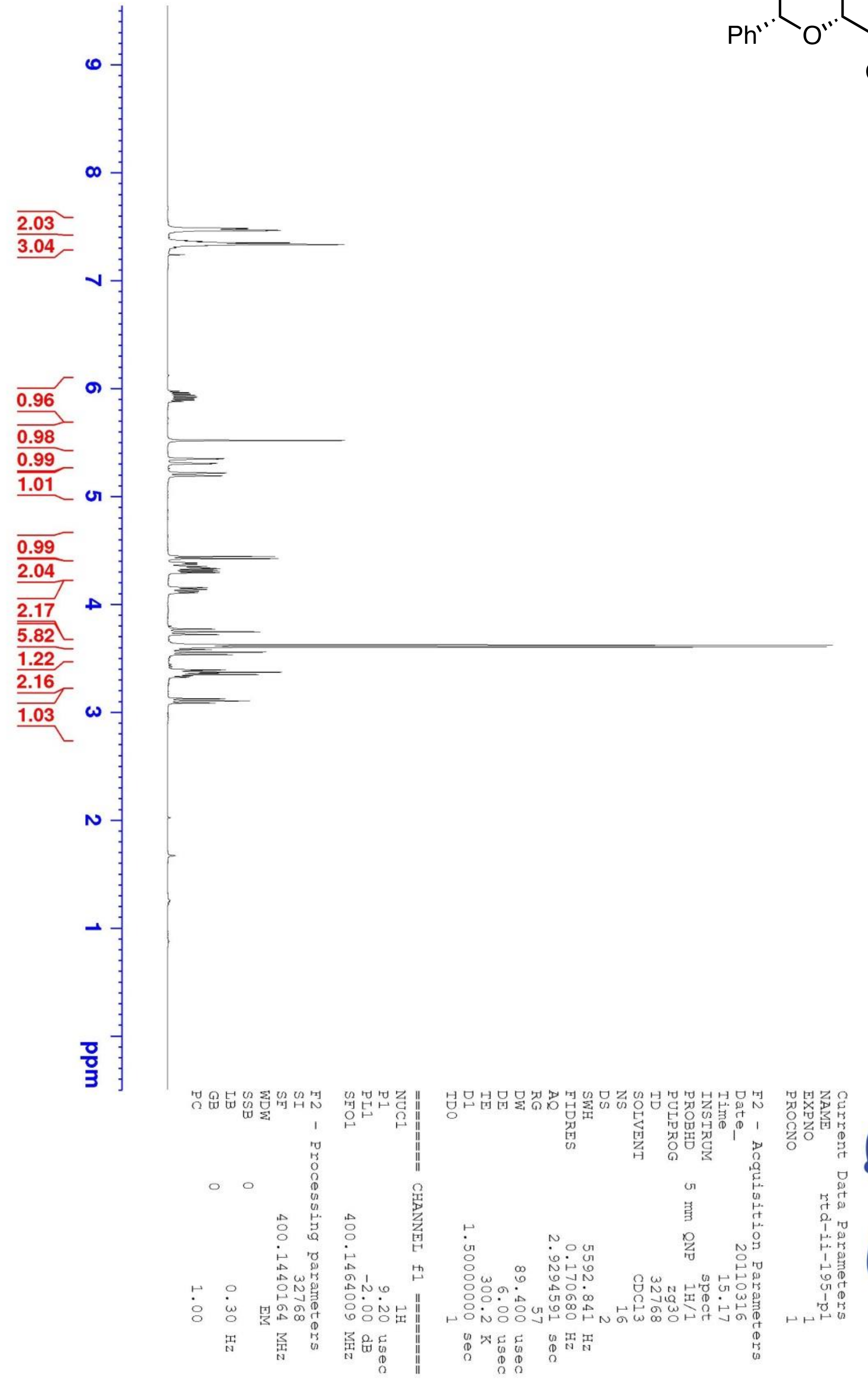
9
8
7
6
5
4
3
2
1
ppm
2.03
3.04
0.96
0.98
0.99
1.01
0.99
2.04
2.17
5.82
1.22
2.16
1.03
Current Data Parameters
NAME rtd-ii-195-p1
EXPNO 1
PROCNO 1
F2 - Acquisition Parameters
Date_ 20110316
Time 15.17
INSTRUM spect
PROBHD 5 mm QNP 1H/1
PULPROG zg30
TD 32768
SOLVENT CDCl3
NS 16
DS 2
SWH 5592.841 Hz
FIDRES 0.170680 Hz
AQ 2.9294591 sec
RG 57
DW 89.400 usec
DE 6.00 usec
TE 300.2 K
D1 1.5000000 sec
TD0 1
======== CHANNEL f1 ========
NUC1 1H
P1 9.20 usec
PL1 -2.00 dB
SFO1 400.1464009 MHz
F2 - Processing parameters
SI 32768
SF 400.1440164 MHz
WDW EM
SSB 0
LB 0.30 Hz
GB 0
PC 1.00

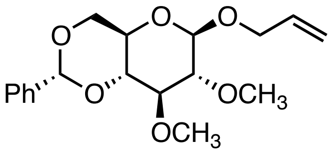
Ph
O
O
O
O
OCH3
OCH3

8b

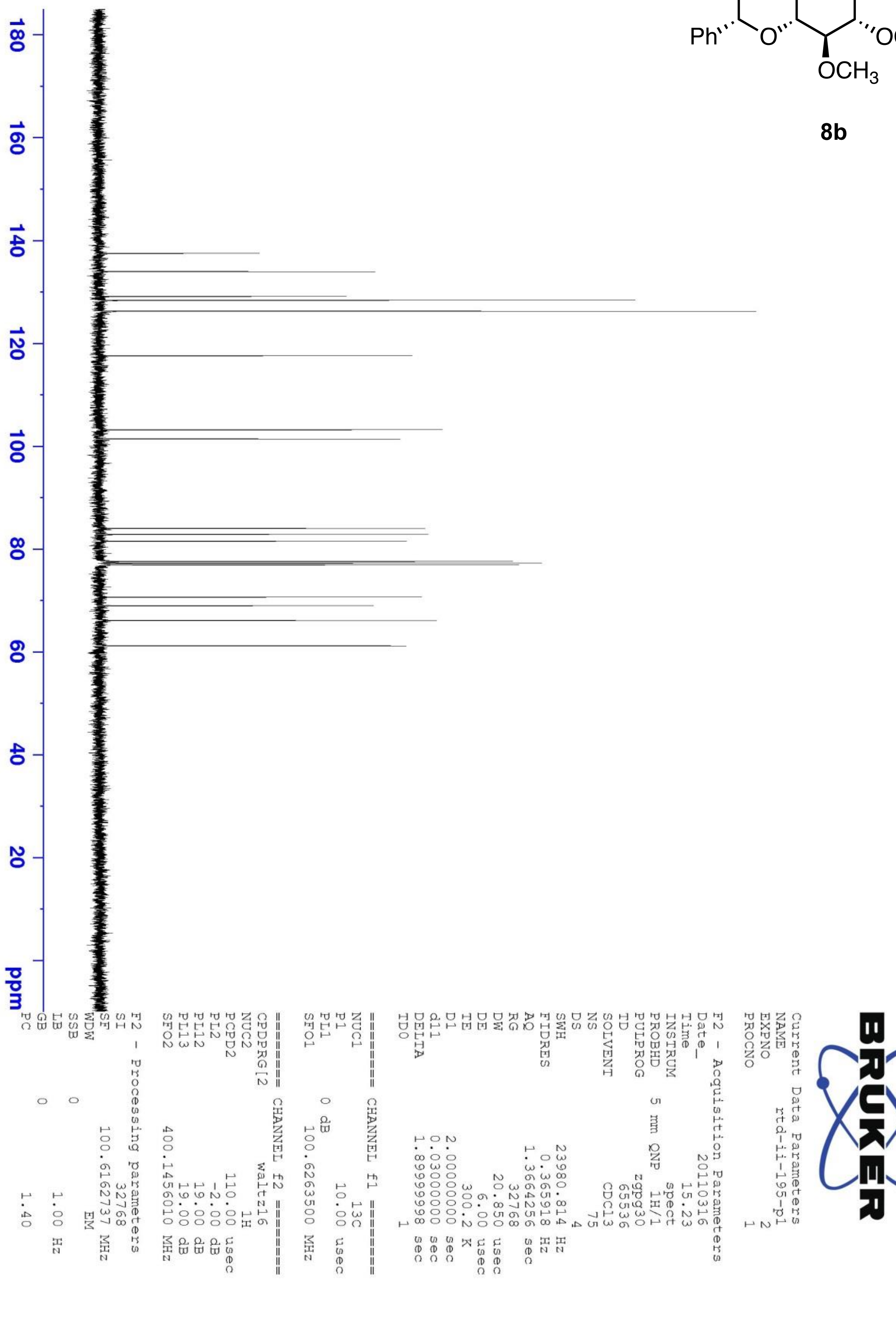
ppm
180
160
140
120
100
80
60
40
20
BRUKER
Current Data Parameters
NAME rtd-ii-195-p1
EXPNO 2
PROCNO 1
F2 - Acquisition Parameters
Date_ 20110316
Time 15.23
INSTRUM spect
PROBHD 5 mm QNP 1H/1
PULPROG zgpg30
TD 65536
SOLVENT CDCl3
NS 75
DS 4
SWH 23980.814 Hz
FIDRES 0.365918 Hz
AQ 1.3664256 sec
RG 32768
DW 20.850 usec
DE 6.00 usec
TE 300.2 K
D1 2.00000000 sec
d11 0.03000000 sec
DELTA 1.89999998 sec
TD0 1
======== CHANNEL f1 ========
NUC1 13C
P1 10.00 usec
PL1 0 dB
SFO1 100.6263500 MHz
======== CHANNEL f2 ========
CPDPRG[2 waltz16
NUC2 1H
PCPD2 110.00 usec
PL2 -2.00 dB
PL12 19.00 dB
PL13 19.00 dB
SFO2 400.1456010 MHz
F2 - Processing parameters
SI 32768
SF 100.6162737 MHz
WDW EM
SSB 0
LB 1.00 Hz
GB 0
PC 1.40

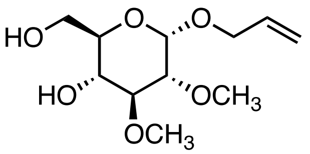
HO
O
O
HO
OCH_3
OCH_3

9a

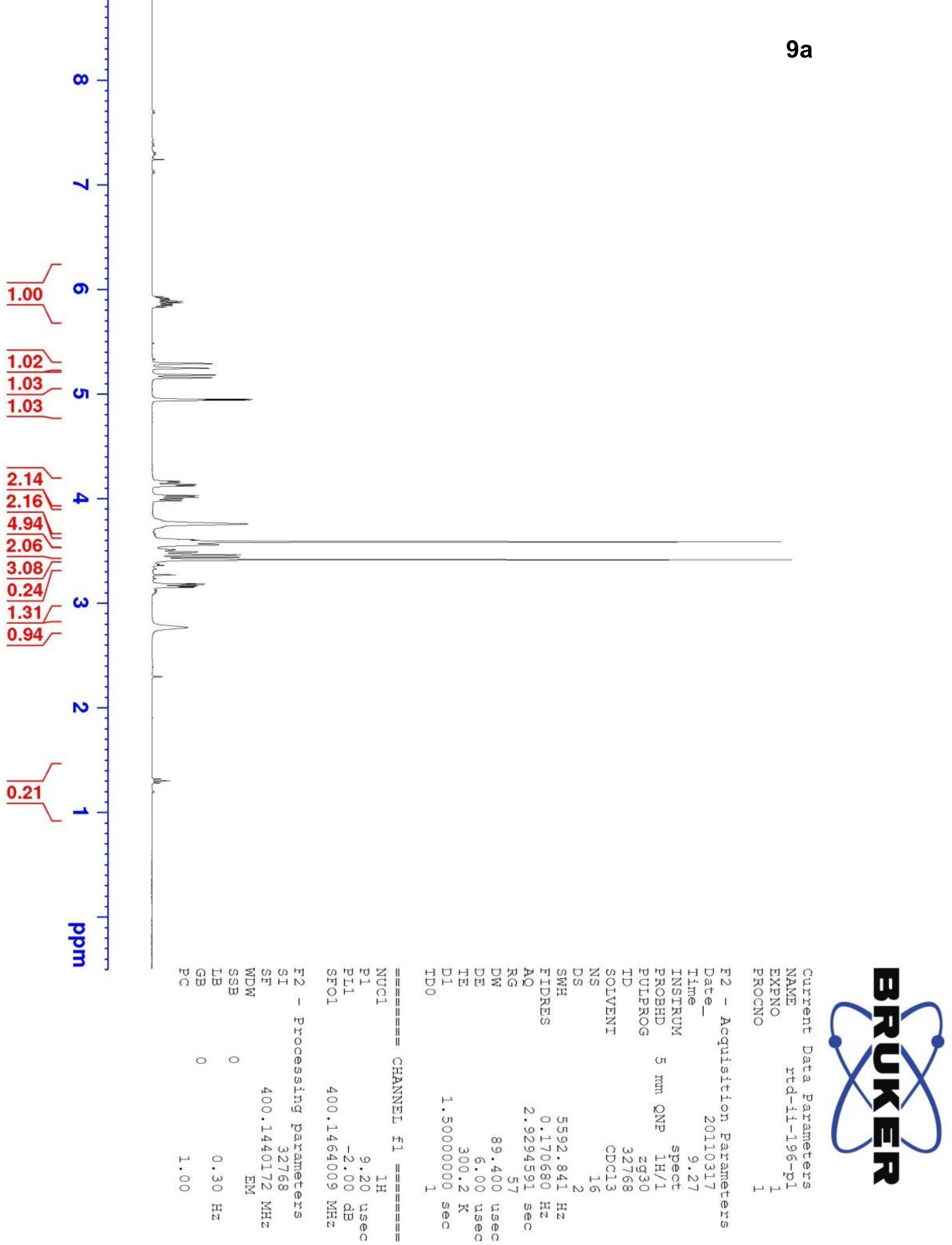
1.00
1.02
1.03
1.03
2.14
2.16
4.94
2.06
3.08
0.24
1.31
0.94
0.21
9
8
7
6
5
4
3
2
1
ppm
Current Data Parameters
NAME rtd-ii-196-p1
EXPNO 1
PROCNO 1
F2 - Acquisition Parameters
Date_ 20110317
Time 9.27
INSTRUM spect
PROBHD 5 mm QNP 1H/1
PULPROG zg30
TD 32768
SOLVENT CDCl3
NS 16
DS 2
SWH 5592.841 Hz
FIDRES 0.170680 Hz
AQ 2.9294591 sec
RG 57
DW 89.400 usec
DE 6.00 usec
TE 300.2 K
D1 1.50000000 sec
TD0 1
======== CHANNEL f1 ========
NUC1 1H
P1 9.20 usec
PL1 -2.00 dB
SFO1 400.1464009 MHz
F2 - Processing parameters
SI 32768
SF 400.1440172 MHz
WDW EM
SSB 0
LB 0.30 Hz
GB 0
PC 1.00

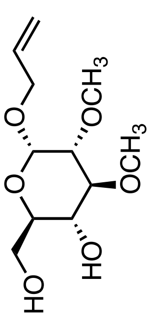
HO
HO
OCH3
OCH3
O
O

9a

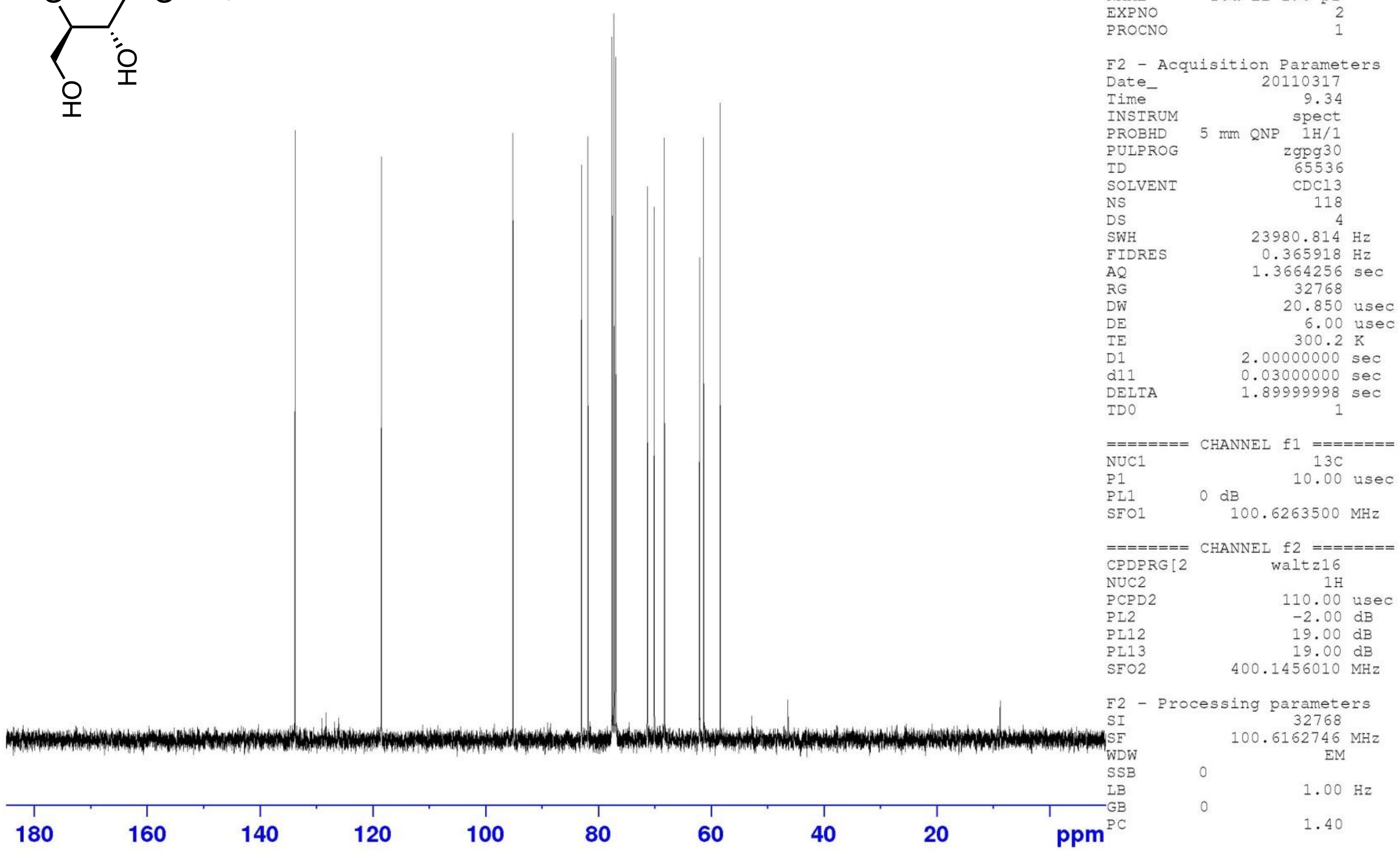
180
160
140
120
100
80
60
40
20
ppm
BRUKER
Current Data Parameters
NAME rtd-ii-196-p1
EXPNO 2
PROCNO 1
F2 - Acquisition Parameters
Date_ 20110317
Time 9.34
INSTRUM spect
PROBHD 5 mm QNP 1H/1
PULPROG zgpg30
TD 65536
SOLVENT CDCl3
NS 118
DS 4
SWH 23980.814 Hz
FIDRES 0.365918 Hz
AQ 1.3664256 sec
RG 32768
DW 20.850 usec
DE 6.00 usec
TE 300.2 K
D1 2.00000000 sec
d11 0.03000000 sec
DELTA 1.89999998 sec
TD0 1
======== CHANNEL f1 ========
NUC1 13C
P1 10.00 usec
PL1 0 dB
SFO1 100.6263500 MHz
======== CHANNEL f2 ========
CPDPRG[2 waltz16
NUC2 1H
PCPD2 110.00 usec
PL2 -2.00 dB
PL12 19.00 dB
PL13 19.00 dB
SFO2 400.1456010 MHz
F2 - Processing parameters
SI 32768
SF 100.6162746 MHz
WDW EM
SSB 0
LB 1.00 Hz
GB 0
PC 1.40

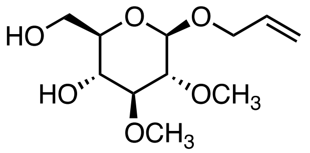
HO
HO
O
O
OCH3
OCH3

9b

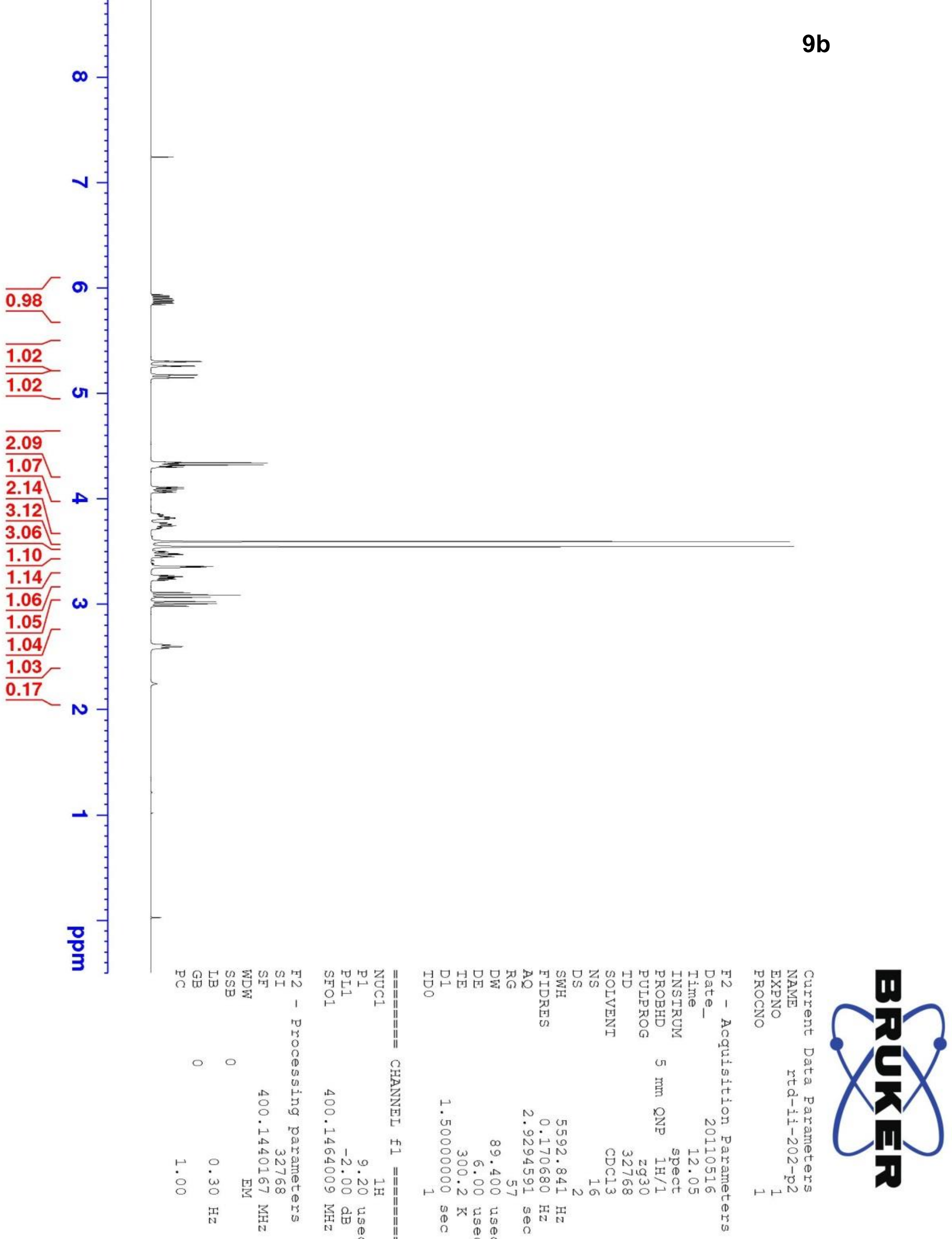
9
8
7
6
5
4
3
2
1
ppm
0.98
1.02
1.02
2.09
1.07
2.14
3.12
3.06
1.10
1.14
1.06
1.05
1.04
1.03
0.17
BRUKER
Current Data Parameters
NAME rtd-ii-202-p2
EXPNO 1
PROCNO 1
F2 - Acquisition Parameters
Date_ 20110516
Time 12.05
INSTRUM spect
PROBHD 5 mm QNP 1H/1
PULPROG zg30
TD 32768
SOLVENT CDCl3
NS 16
DS 2
SWH 5592.841 Hz
FIDRES 0.170680 Hz
AQ 2.9294591 sec
RG 57
DW 89.400 usec
DE 6.00 usec
TE 300.2 K
D1 1.50000000 sec
TD0 1
======== CHANNEL f1 ========
NUC1 1H
P1 9.20 usec
PL1 -2.00 dB
SFO1 400.1464009 MHz
F2 - Processing parameters
SI 32768
SF 400.1440167 MHz
WDW EM
SSB 0
LB 0.30 Hz
GB 0
PC 1.00

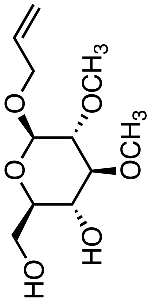
HO
HO
O
O
OCH3
OCH3

9b

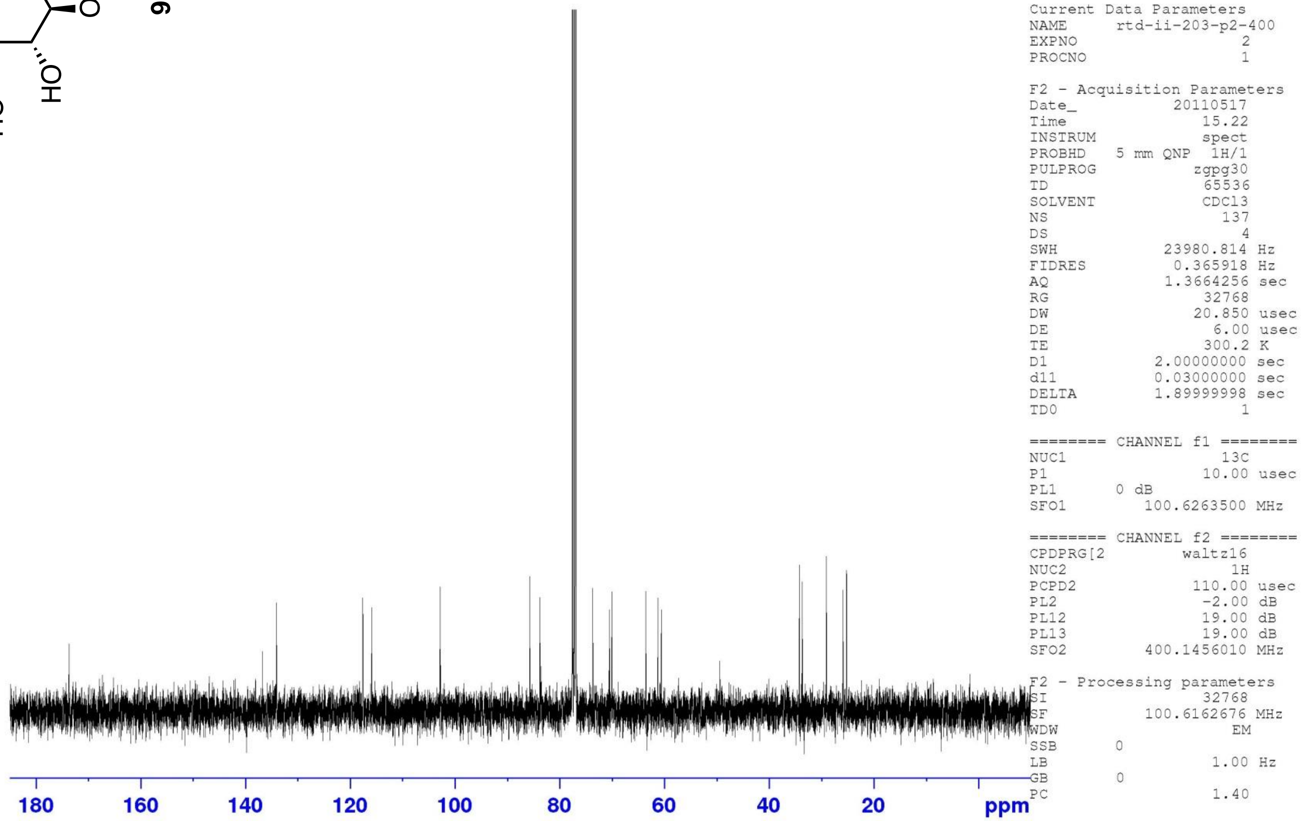
180
160
140
120
100
80
60
40
20
ppm
BRUKER
Current Data Parameters
NAME rtd-ii-203-p2-400
EXPNO 2
PROCNO 1
F2 - Acquisition Parameters
Date_ 20110517
Time 15.22
INSTRUM spect
PROBHD 5 mm QNP 1H/1
PULPROG zgpg30
TD 65536
SOLVENT CDCl3
NS 137
DS 4
SWH 23980.814 Hz
FIDRES 0.365918 Hz
AQ 1.3664256 sec
RG 32768
DW 20.850 usec
DE 6.00 usec
TE 300.2 K
D1 2.00000000 sec
d11 0.03000000 sec
DELTA 1.89999998 sec
TD0 1
======== CHANNEL f1 ========
NUC1 13C
P1 10.00 usec
PL1 0 dB
SFO1 100.6263500 MHz
======== CHANNEL f2 ========
CPDPRG[2 waltz16
NUC2 1H
PCPD2 110.00 usec
PL2 -2.00 dB
PL12 19.00 dB
PL13 19.00 dB
SFO2 400.1456010 MHz
F2 - Processing parameters
SI 32768
SF 100.6162676 MHz
WDW EM
SSB 0
LB 1.00 Hz
GB 0
PC 1.40

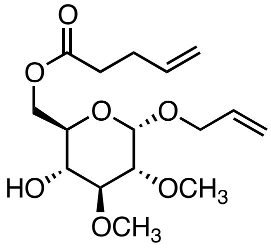
O
O
O
O
HO
OCH3
OCH3

10a

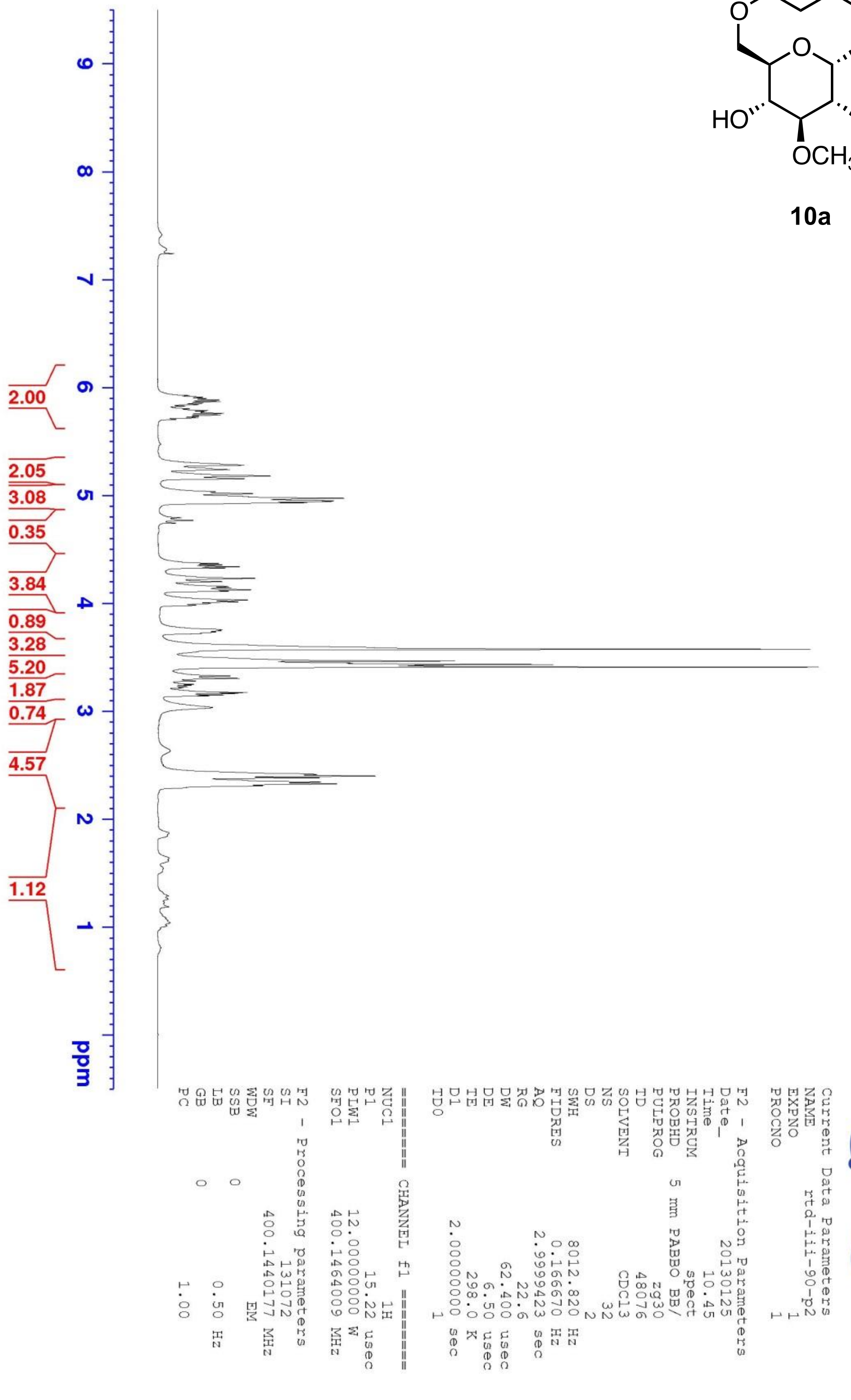
ppm
9
8
7
6
5
4
3
2
1
2.00
2.05
3.08
0.35
3.84
0.89
3.28
5.20
1.87
0.74
4.57
1.12
Current Data Parameters
NAME rtd-iii-90-p2
EXPNO 1
PROCNO 1
F2 - Acquisition Parameters
Date_ 20130125
Time 10.45
INSTRUM spect
PROBHD 5 mm PABBO BB/
PULPROG zg30
TD 48076
SOLVENT CDCl3
NS 32
DS 2
SWH 8012.820 Hz
FIDRES 0.166670 Hz
AQ 2.9999423 sec
RG 22.6
DW 62.400 usec
DE 6.50 usec
TE 298.0 K
D1 2.00000000 sec
TD0 1
======== CHANNEL f1 ========
NUC1 1H
P1 15.22 usec
PLW1 12.00000000 W
SFO1 400.1464009 MHz
F2 - Processing parameters
SI 131072
SF 400.1440177 MHz
WDW EM
SSB 0
LB 0.50 Hz
GB 0
PC 1.00

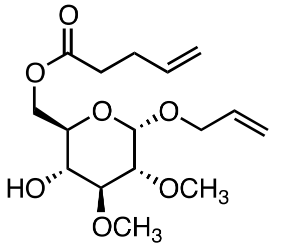

10a

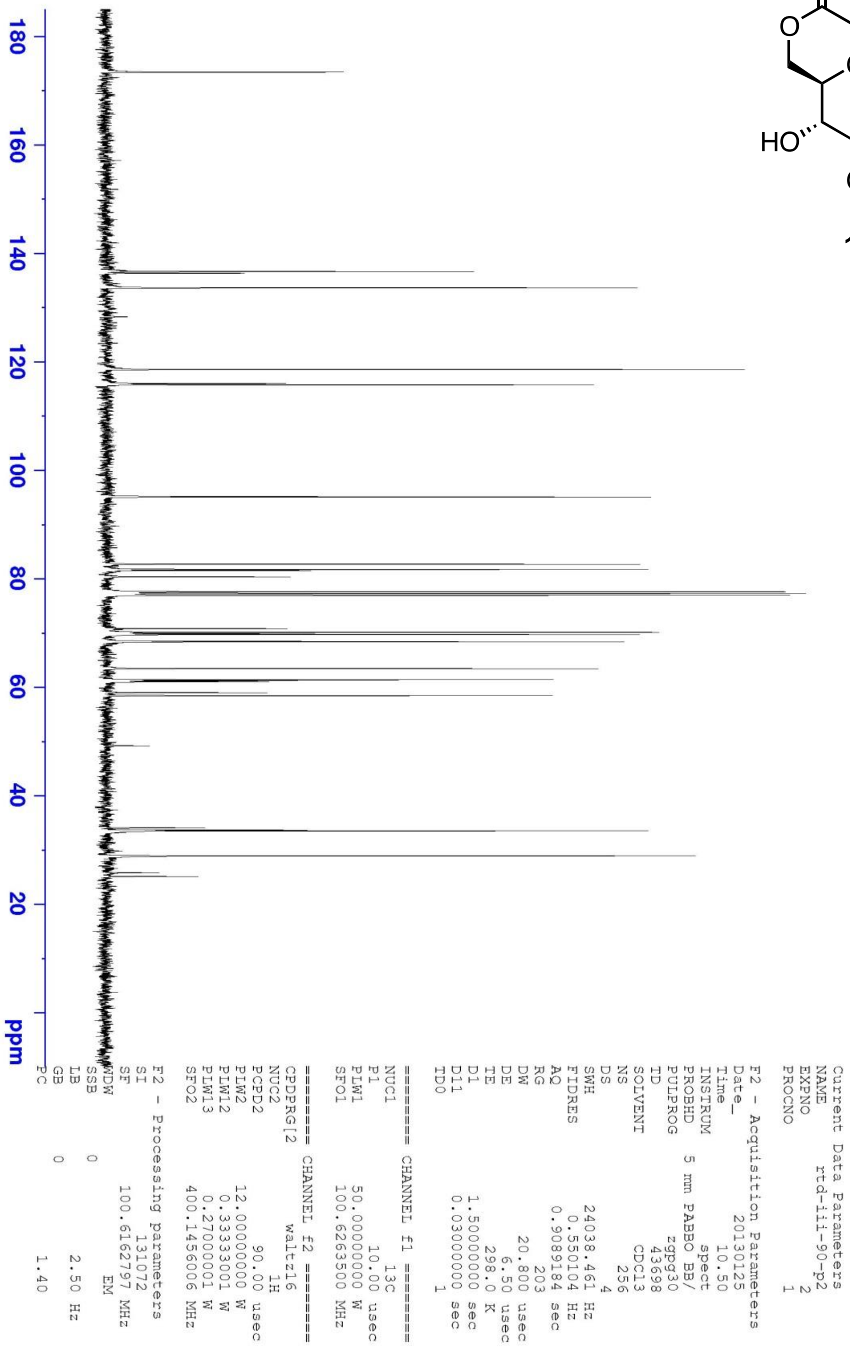
180
160
140
120
100
80
60
40
20
ppm
Current Data Parameters
NAME rtd-iii-90-p2
EXPNO 2
PROCNO 1
F2 - Acquisition Parameters
Date_ 20130125
Time 10.50
INSTRUM spect
PROBHD 5 mm PABBO BB/
PULPROG zgpg30
TD 43698
SOLVENT CDCl3
NS 256
DS 4
SWH 24038.461 Hz
FIDRES 0.550104 Hz
AQ 0.9089184 sec
RG 203
DW 20.800 usec
DE 6.50 usec
TE 298.0 K
D1 1.50000000 sec
D11 0.03000000 sec
TD0 1
======== CHANNEL f1 ========
NUC1 13C
P1 10.00 usec
PLW1 50.00000000 W
SFO1 100.6263500 MHz
======== CHANNEL f2 ========
CPDPRG[2 waltz16
NUC2 1H
PCPD2 90.00 usec
PLW2 12.00000000 W
PLW12 0.33333001 W
PLW13 0.27000001 W
SFO2 400.1456006 MHz
F2 - Processing parameters
SI 131072
SF 100.6162797 MHz
WDW EM
SSB 0
LB 2.50 Hz
GB 0
PC 1.40

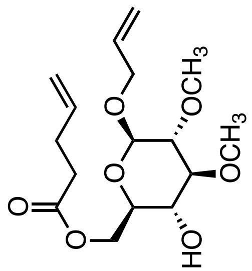

10b

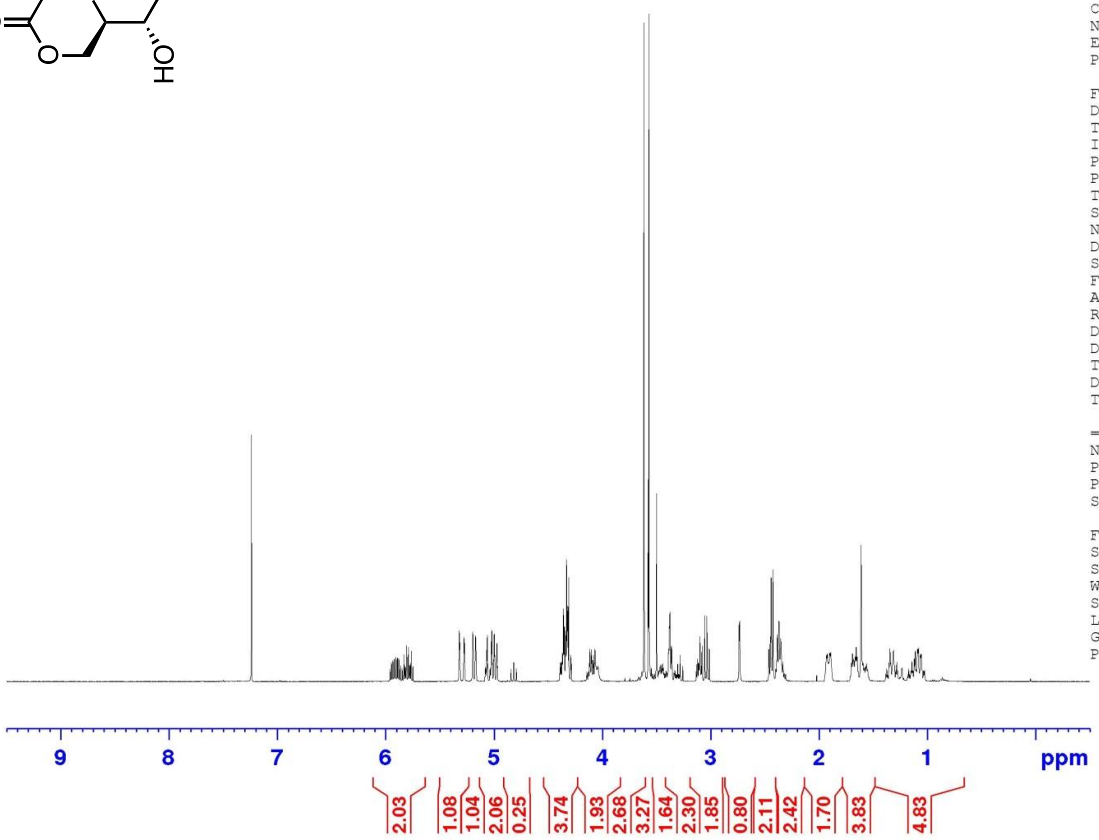


```
Current Data Parameters
NAME      rtd-ii-203-p2-400
EXPNO                     1
PROCNO                    1

F2 - Acquisition Parameters
Date_              20110517
Time                  15.17
INSTRUM               spect
PROBHD    5 mm QNP   1H/1
PULPROG                zg30
TD                    32768
SOLVENT               CDCl3
NS                       16
DS                        2
SWH                5592.841 Hz
FIDRES             0.170680 Hz
AQ                2.9294591 sec
RG                    322.5
DW                   89.400 usec
DE                     6.00 usec
TE                    300.2 K
D1               1.50000000 sec
TD0                       1

======== CHANNEL f1 ========
NUC1                     1H
P1                     9.20 usec
PL1                   -2.00 dB
SFO1            400.1464009 MHz

F2 - Processing parameters
SI                    32768
SF              400.1440168 MHz
WDW                      EM
SSB       0
LB                     0.30 Hz
GB        0
PC                     1.00
```

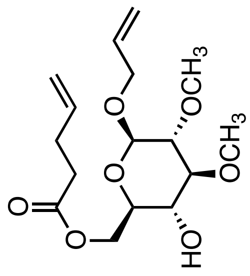

10b

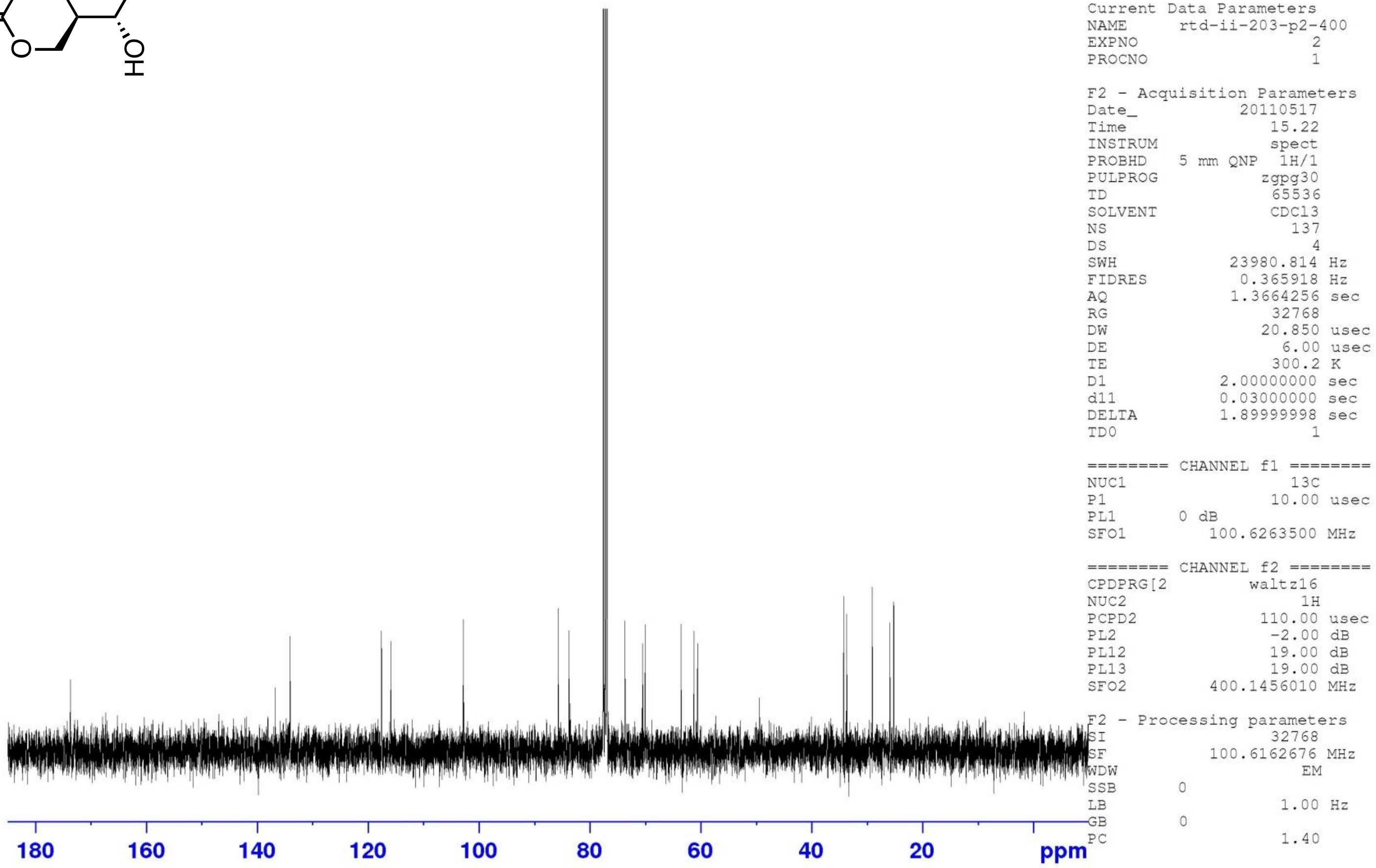
180
160
140
120
100
80
60
40
20
ppm
BRUKER
Current Data Parameters
NAME rtd-ii-203-p2-400
EXPNO 2
PROCNO 1
F2 - Acquisition Parameters
Date_ 20110517
Time 15.22
INSTRUM spect
PROBHD 5 mm QNP 1H/1
PULPROG zgpg30
TD 65536
SOLVENT CDCl3
NS 137
DS 4
SWH 23980.814 Hz
FIDRES 0.365918 Hz
AQ 1.3664256 sec
RG 32768
DW 20.850 usec
DE 6.00 usec
TE 300.2 K
D1 2.00000000 sec
d11 0.03000000 sec
DELTA 1.89999998 sec
TD0 1
======== CHANNEL f1 ========
NUC1 13C
P1 10.00 usec
PL1 0 dB
SFO1 100.6263500 MHz
======== CHANNEL f2 ========
CPDPRG[2 waltz16
NUC2 1H
PCPD2 110.00 usec
PL2 -2.00 dB
PL12 19.00 dB
PL13 19.00 dB
SFO2 400.1456010 MHz
F2 - Processing parameters
SI 32768
SF 100.6162676 MHz
WDW EM
SSB 0
LB 1.00 Hz
GB 0
PC 1.40

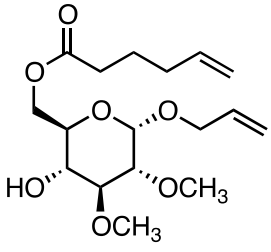
O
O
O
O
HO
OCH3
OCH3

10c

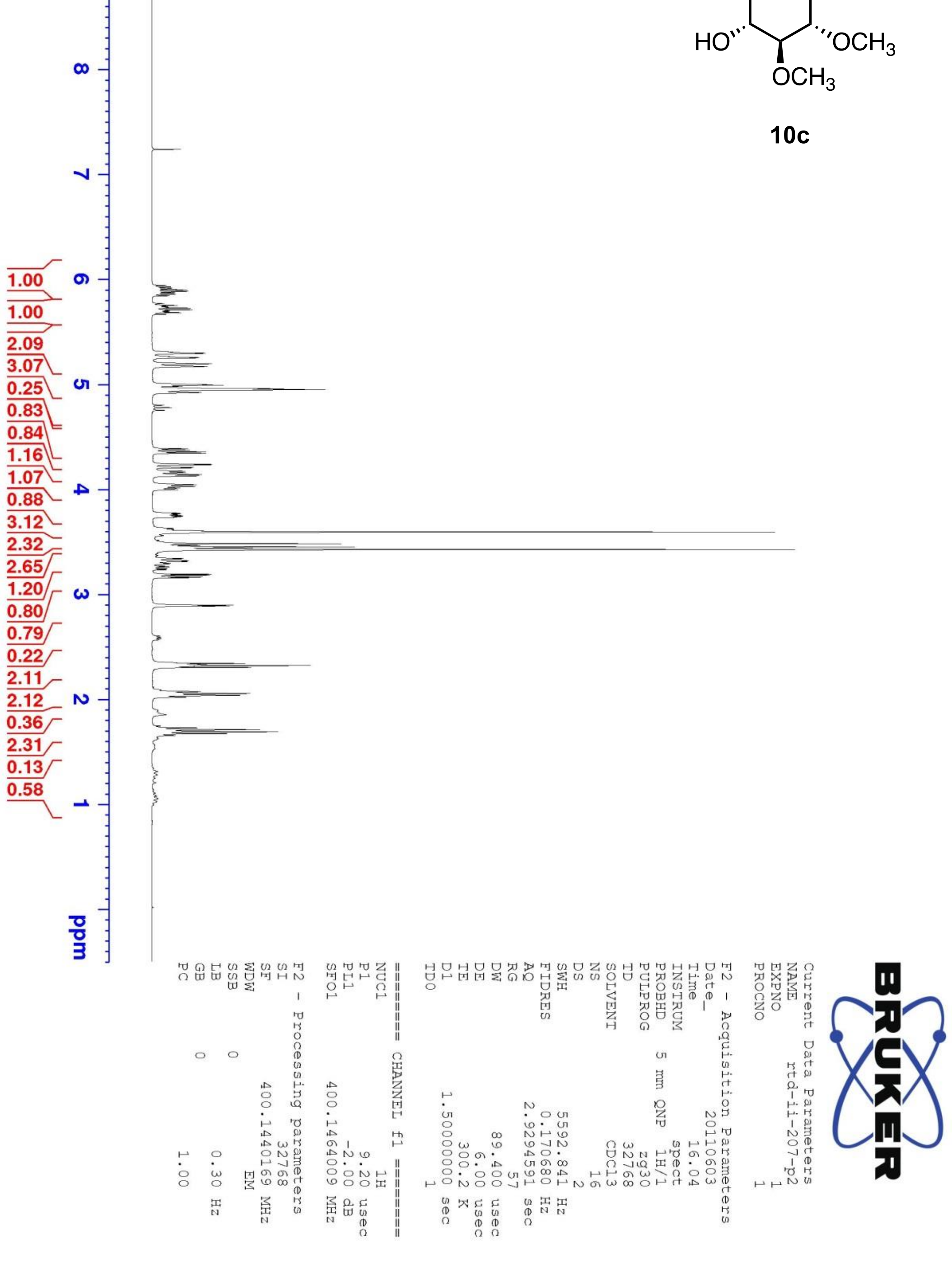
9
8
7
6
5
4
3
2
1
ppm
1.00
1.00
2.09
3.07
0.25
0.83
0.84
1.16
1.07
0.88
3.12
2.32
2.65
1.20
0.80
0.79
0.22
2.11
2.12
0.36
2.31
0.13
0.58
BRUKER
Current Data Parameters
NAME rtd-ii-207-p2
EXPNO 1
PROCNO 1
F2 - Acquisition Parameters
Date_ 20110603
Time 16.04
INSTRUM spect
PROBHD 5 mm QNP 1H/1
PULPROG zg30
TD 32768
SOLVENT CDCl3
NS 16
DS 2
SWH 5592.841 Hz
FIDRES 0.170680 Hz
AQ 2.9294591 sec
RG 57
DW 89.400 usec
DE 6.00 usec
TE 300.2 K
D1 1.50000000 sec
TD0 1
======== CHANNEL f1 ========
NUC1 1H
P1 9.20 usec
PL1 -2.00 dB
SFO1 400.1464009 MHz
F2 - Processing parameters
SI 32768
SF 400.1440169 MHz
WDW EM
SSB 0
LB 0.30 Hz
GB 0
PC 1.00

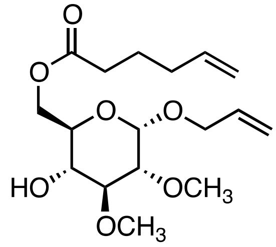
O
O
O
O
HO
OCH3
OCH3

10c

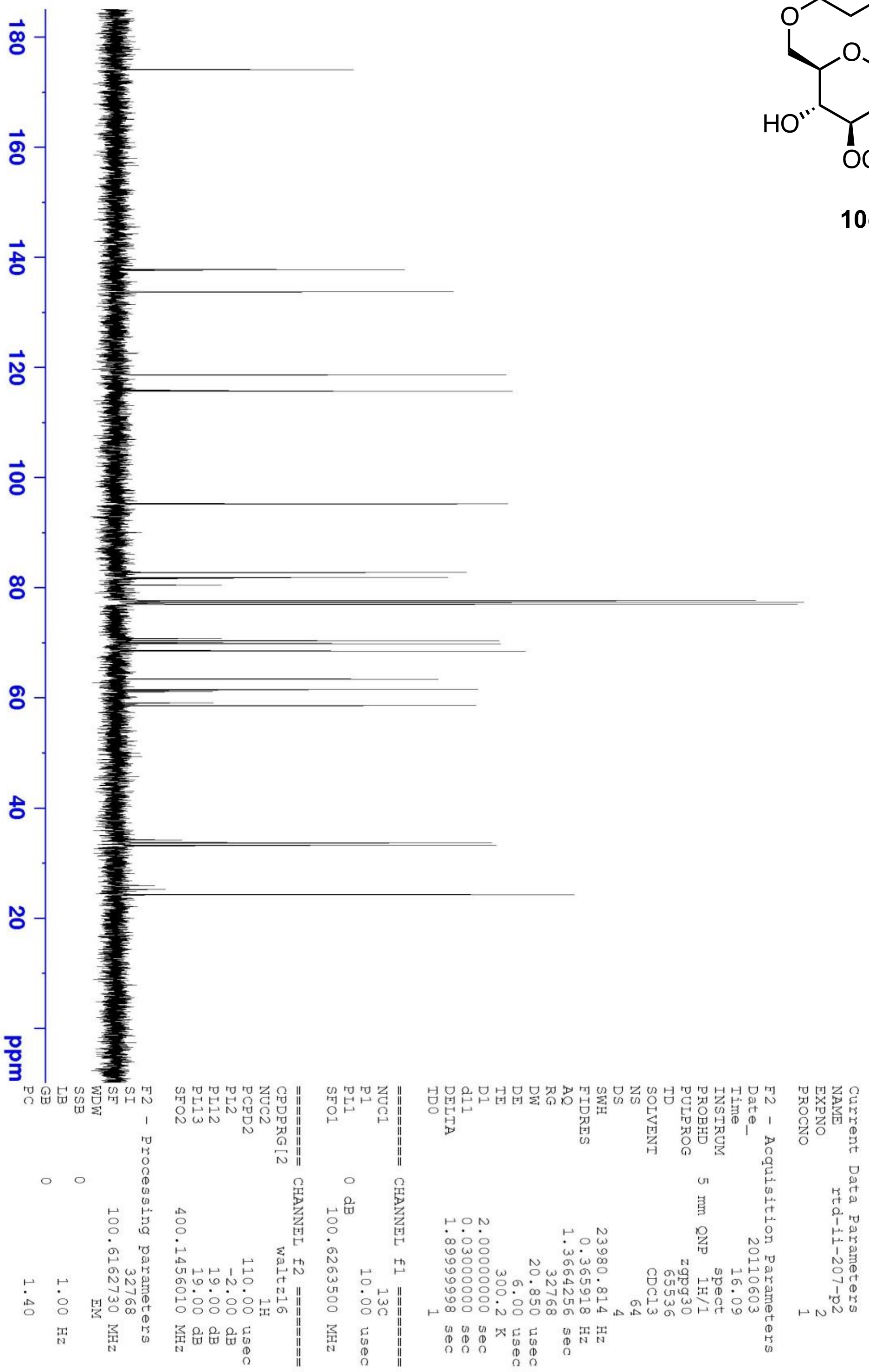
180
160
140
120
100
80
60
40
20
ppm
BRUKER
Current Data Parameters
NAME rtd-ii-207-p2
EXPNO 2
PROCNO 1
F2 - Acquisition Parameters
Date_ 20110603
Time 16.09
INSTRUM spect
PROBHD 5 mm QNP 1H/1
PULPROG zgpg30
TD 65536
SOLVENT CDCl3
NS 64
DS 4
SWH 23980.814 Hz
FIDRES 0.365918 Hz
AQ 1.3664256 sec
RG 32768
DW 20.850 usec
DE 6.00 usec
TE 300.2 K
D1 2.00000000 sec
d11 0.03000000 sec
DELTA 1.89999998 sec
TD0 1
======== CHANNEL f1 ========
NUC1 13C
P1 10.00 usec
PL1 0 dB
SFO1 100.6263500 MHz
======== CHANNEL f2 ========
CPDPRG[2 waltz16
NUC2 1H
PCPD2 110.00 usec
PL2 -2.00 dB
PL12 19.00 dB
PL13 19.00 dB
SFO2 400.1456010 MHz
F2 - Processing parameters
SI 32768
SF 100.6162730 MHz
WDW EM
SSB 0
LB 1.00 Hz
GB 0
PC 1.40

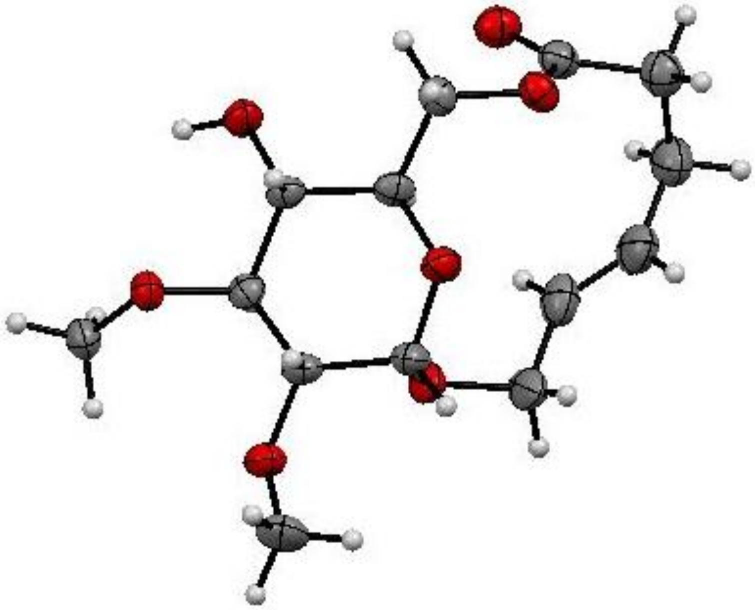

Figure S1. ORTEP of compound **5**.

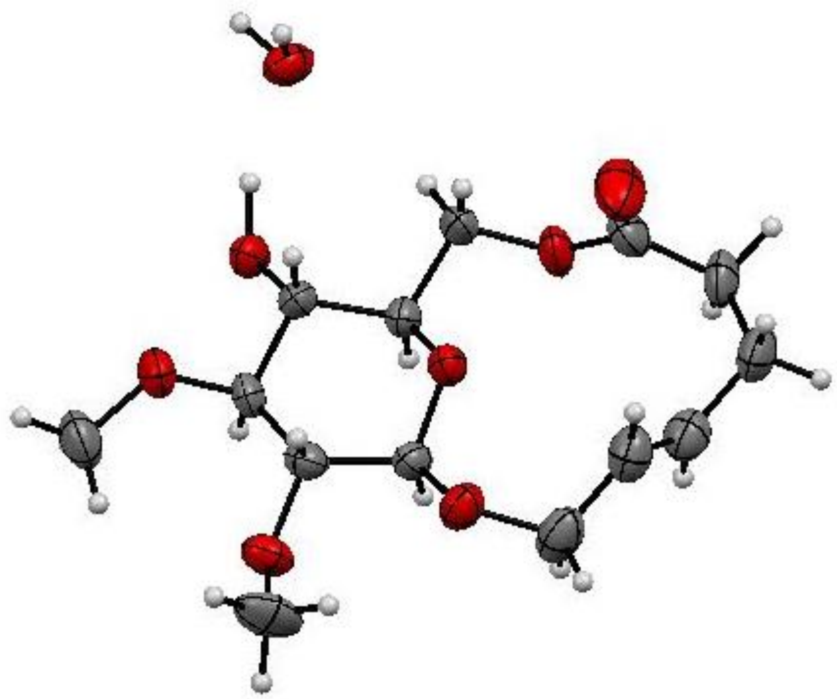

Figure S2. ORTEP of compound **6**.
