# Supplementary material for: De novo macrolide–glycolipid macrolactone hybrids: Synthesis, structure and antibiotic activity of carbohydrate-fused macrocycles
Source: Beilstein J Org Chem. 2014 Sep 17;10:2215–21. doi: 10.3762/bjoc.10.229 (PMC4168945; doi:10.3762/bjoc.10.229)
Supplement: File 2 — Experimental procedures and characterization of all new compounds. [file Beilstein_J_Org_Chem-10-2215-s002.pdf]

# Supporting Information

**for**

# De novo macrolide – glycolipid macrolactone hybrids: Synthesis, structure and antibiotic activity of carbohydrate-fused macrocycles

Richard T. Desmond[1], Anniefer N. Magpusao[1], Chris Lorenc[1], Jeremy B. Alverson[2], Nigel Priestley[2], and Mark W. Peczuh[1]*

Address: [1]Department of Chemistry, University of Connecticut, 55 N. Eagleville Road, U3060, Storrs, CT 06269 USA, +1-860-486-1605 FAX: +1-860-486-2981 and [2]Department of Chemistry and Biochemistry, University of Montana, Missoula, MT 59812, USA

Email: Mark W. Peczuh - mark.peczuh@uconn.edu

Experimental procedures and characterization of all new compounds.

**Experimental**

**Allyl 4,6-*O*-benzylidene-2,3-di-*O*-methyl-α-D-glucopyranoside (8a) and allyl 4,6-*O*-benzylidene-2,3-di-*O*-methyl-β-D-glucopyranoside (8b).** To an ice-cold solution of allyl 4,6-*O*-benzylidene-D-glucopyranoside **7** (2.00 g, 6.48 mmol), as a mixture of α/β–anomers, in dry 5 mL DMF under $N_2$ was added sodium hydride (0.623 g 60% dispersion in oil, 15.5 mmol). Tetrabutyl ammonium iodide (0.718 g, 1.90 mmol) was added and then iodomethane (1.00 mL, 15.5 mmol) was introduced dropwise via a syringe. The ice bath was removed and the mixture was allowed to warm to room temperature. Upon disappearance of starting material via TLC, the reaction was quenched with water (0.5 mL) and concentrated under reduced pressure. The mixture was then redissolved in DCM (50 mL) and washed with brine (2 x 50 mL) and dried over sodium sulfate. The solution was filtered and the solvent was removed under reduced pressure. Column chromatography (4:1 Hex:EtOAc) yielded **8a** and **8b** in a 3:1 (α:β) ratio (66% combined yield) as white solids. The first fraction was identified as the β-anomer (0.359 g, 17%) and the second as the α-anomer (1.07 g, 49%)

**Allyl 4,6-O-benzylidene-2,3-di-O-methyl-α-D-glucopyranoside (8a).** Isolated as a white solid (1.07 g, 49%) m.p. 98.4-99.7 °C; $R_f$ 0.32 (9:1, Hex:EtOAc); $[\alpha]_D$ +58.5° (*c* 2.7, $CHCl_3$); $^1H$ NMR ($CDCl_3$) 400MHz δ 7.48-7.30 (m, 5H), 5.92 (dddd, 1H, *J* = 16.9, 10.6, 5.8, 5.8 Hz), 5.51 (s, 1H), 5.31 (dd, 1H, *J* = 17.2, 1.0 Hz), 5.20 (d, 1H, *J* = 10.6 Hz), 4.98 (d, 1H, *J* = 3.7 Hz), 4.25-4.16 (m, 2H), 4.04 (dd, 1H, *J* = 12.8, 6.7 Hz), 3.84 (ddd, 1H, *J* = 14.2, 10.0, 4.8 Hz), 3.68 (ddd, 2H, *J* = 14.2, 9.2, 3.9 Hz), 3.61 (s, 3H), 3.51 (d, 1H, *J* = 9.6 Hz), 3.49 (s, 3H), 3.27 (dd, 1H, *J* = 9.2, 3.7 Hz); $^{13}C$ NMR ($CDCl_3$) 100 MHz δ 137.5, 133.7, 129.0, 128.3, 126.2, 118.5, 101.4, 96.1, 82.4, 81.4, 79.8, 69.1, 68.5, 62.6, 61.1, 59.1; TOF HRMS (DART) *m/z* calc'd for $C_{18}H_{25}O_6$ $[M+H]^+$ 337.1651, obs. 337.1646.

**Allyl 4,6-O-benzylidene-2,3-di-O-methyl-β-D-glucopyranoside (8b).** Isolated as a white solid (0.359 g, 17%). m.p. 108.7-110.6 °C; $R_f$ 0.47 (9:1, Hex:EtOAc); $[\alpha]_D$ -30.2° (*c* 2.8, $CHCl_3$); $^1H$ NMR ($CDCl_3$) 400MHz δ 7.47 (m, 2H), 7.37-7.32 (m, 3H), 5.93 (dddd, 1H, *J* = 16.3, 10.7, 5.4, 5.4 Hz), 5.52 (s, 1H), 5.33 (dd, 1H, *J* = 17.2, 1.5 Hz), 5.20 (dd, 1H, *J* = 10.4, 1.2 Hz), 4.43 (d, 1H, J = 7.6 Hz), 4.36 (dd, 1H, *J* = 13.0, 5.2 Hz), 4.31 (dd, 1H, *J* = 10.5, 5.0 Hz), 3.74 (m, 2H), 3.62 (s, 3H), 3.60 (s, 3H), 3.56 (dd 1H, *J* = 9.3, 9.3 Hz), 3.39-3.32 (m, 2H), 3.10 (dd, 1H, *J* = 7.9, 7.9 Hz); $^{13}C$ NMR ($CDCl_3$) 100 MHz δ 137.5, 133.9, 129.1, 128.4, 126.2, 117.6, 103.2, 101.4, 84.0, 82.9, 81.5, 70.6, 68.9, 66.1, 61.2, 61.1; TOF HRMS (DART) *m/z* calc'd for $C_{18}H_{25}O_6$ $[M+H]^+$ 337.1651, obs. 337.1648.

***Benzylidene Deprotection.*** To an 0.08 M solution of substrate (i.e., **8a** or **8b**) in 3:1 DCM:MeOH, 0.5 equivalents of *p*-TsOH were added with stirring at rt. The mixture was then brought to reflux on an oil bath until complete conversion as observed via TLC (1-3 hrs). The reaction was subsequently allowed to cool to room temperature and quenched with trimethylamine (0.5 eq.). The mixture was concentrated under reduced pressure and purified by column chromatography (DCM:MeOH, 9:1).

**Allyl 2,3-di-*O*-methyl-α-D-glucopyranoside (9a).** Isolated as a clear colorless oil (0.861 g, 3.47 mmol, quant.). $R_f$ 0.19 (9:1 DCM:MeOH); $[\alpha]_D$ +112.5° (*c* 4.8, $CHCl_3$); $^1H$ NMR ($CDCl_3$) 400MHz δ 5.98 (dddd,

1H, *J* = 17.0, 10.5, 6.4, 5.5 Hz) 5.27 (dd, 1H, *J* = 17.2, 1.2 Hz), 5.17 (d, 1H, *J* = 10.4 Hz), 4.94 (d, 1H, *J* = 3.5 Hz), 4.15 (dd, 1H, *J* = 12.9, 5.2 Hz), 4.00 (dd, 1H, *J* = 12.9, 6.6 Hz), 3.75 (br s, 2H), 3.61-3.56 (m, 2H), 3.59 (s, 3H), 3.47 (m, 2H), 3.41, (s, 3H), 3.16 (dd, 1H, *J* = 9.0, 3.5 Hz), 2.76 (br s, 1H); $^{13}$C NMR ($CDCl_3$) 100 MHz δ 133.8, 118.4, 95.1, 83.0, 81.8, 71.3, 70.1, 68.3, 62.0, 61.3, 58.4; TOF HRMS (DART) *m/z* calc'd for $C_{11}H_{21}O_6$ $[M+H]^+$ 249.1338, obs. 249.1314; for $C_{11}H_{24}NO_6$ $[M+NH_4]^+$ 266.1604, obs. 266.1556.

**Allyl 2,3-di-*O*-methyl-β-D-glucopyranoside (9b).** Isolated as a clear colorless oil (0.267 g, 1.08 mmol, quant.). $R_f$ 0.38 (9:1, DCM:MeOH); $[\alpha]_D$ -24.6° (*c* 1.0, $CHCl_3$); $^1$H NMR ($CDCl_3$) 400MHz δ 5.89 (dddd, 1H, *J* = 16.1, 10.7, 6.7, 6.7 Hz), 5.28 (ddd, 1H, *J* = 17.2, 3.4, 1.6 Hz), 5.16 (ddd, 1H, *J* = 10.4, 2.8, 1.4, Hz), 4.35-4.29 (m, 2H), 3.85-3.80 (m, 2H), 3.59 (s, 3H), 3.54 (s, 3H), 3.50 (ddd, 1H, *J* = 9.4, 9.4, 3.2, Hz), 3.35 (d, 1H, *J* = 3.8 Hz), 3.25 (ddd, 1H, *J* = 9.6, 4.5, 3.5 Hz), 3.08 (dd, 1H, *J* = 9.0, 9.0 Hz), 3.00 (dd, 1H, *J* = 9.0, 7.6 Hz), 2.60 (dd, 1H, *J* = 6.4, 6.4 Hz); $^{13}$C NMR ($CDCl_3$) 100 MHz δ 134.1, 117.4, 102.9, 86.0, 83.3, 75.2, 70.6, 70.1, 62.5, 61.1, 60.5; TOF HRMS (DART) *m/z* calc'd for $C_{11}H_{21}O_6$ $[M+H]^+$ 249.1338, obs. 249.1320.

**C6-*O*-Acylation.** To solution of the diol dissolved in dry DCM (10 mM) at 0 °C under $N_2$ was added 1.2 equiv. of either 4-pentenoic or 5-hexenoic acid (via syringe), 1.4 equiv. DCC and 0.5 equiv. DMAP. The mixture was allowed to come to room temperature while being monitored via TLC over the course of 1-3 h. When starting material was no longer visible, the reaction was diluted with hexanes (50 mL) and filtered through a pad of celite. The celite was the washed with EtOAc (50 mL). The combined filtrates were collected and concentrated under reduced pressure and the mixture was purified by column chromatography eluting with Hex:EtOAc mixtures corresponding to TLC conditions to deliver the corresponding diene.

**Allyl 2,3-di-*O*-methyl-6-*O*-(4-pentenoyl)-α-D-glucopyranoside (10a).** The diene was isolated as a clear, colorless oil (0.356 g, 1.08 mmol, 58%). $R_f$ 0.54 (1:1 Hex:EtOAc); $[\alpha]_D$ +5.6° (*c* 2.7, $CHCl_3$); $^1$H NMR ($CDCl_3$) 400MHz δ 5.87 (dddd, 1H, *J* = 16.7, 10.6, 5.6, 5.6 Hz), 5.75 (dddd, 1H, *J* = 16.6, 10.5, 6.2, 6.2 Hz), 5.26 (d, 1H, *J* = 17.1 Hz), 5.17 (d, 1H, *J* = 10.0 Hz), 5.03-4.93 (m, 3H), 4.34 (dd, 1H, *J* = 12.0, 5.0 Hz), 4.21 (d, 1H, *J* = 4.2), 4.13 (dd, 1H, *J* = 12.6, 5.2 Hz), 4.00 (dd, 1H, *J* = 12.7, 6.6 Hz), 3.73 (m, 1H), 3.57 (s, 3H), 3.46-3.40 (m, 5H), 3.15 (dd, 1H, *J* = 9.4, 3.5 Hz), 2.36 (m, 4H); $^{13}$C NMR ($CDCl_3$) 100 MHz δ 173.4, 136.6, 133.6, 118.5, 115.7, 95.0, 82.6, 81.7, 70.1, 69.7, 68.3, 63.4, 61.3, 58.4, 33.5, 28.9; TOF HRMS (DART) *m/z* calc'd for $C_{16}H_{27}O_7$ $[M+H]^+$ 331.1757, obs. 331.1771; for $C_{16}H_{30}NO_7$ $[M+NH_4]^+$ 348.2022, obs. 348.2066.

**Allyl 2,3-di-*O*-methyl-6-*O*-(4-pentenoyl)-β-D-glucopyranoside (10b)** The diene was isolated as a clear, colorless oil (141 mg, 0.427 mmol, 56%). $R_f$ 0.62 (1:1 Hex:EtOAc); $[\alpha]_D$ -4.3° (*c* 1.6, $CHCl_3$); $^1$H NMR ($CDCl_3$) 400MHz δ 5.86 (dddd, 1H, *J* = 17.2, 10.6, 5.9, 5.2 Hz), 5.75 (dddd, 1H, *J* = 16.7, 10.2, 6.4, 6.4 Hz), 5.24 (ddd, 1H, *J* = 17.1, 3.4, 1.6 Hz), 5.13 (ddd, 1H, *J* = 10.4, 4.8, 1.3 Hz), 4.99 (ddd, 1H, *J* = 17.2, 3.2, 1.6 Hz), 4.93 (ddd, 1H, *J* = 10.2, 2.7, 1.2 Hz), 4.32- 4.25 (m, 3H), 4.06, (m 2H), 3.56 (s,

3H), 3.52 (s, 3H), 3.36- 3.28 (m, 2H), 3.06 (dd, 1H, *J* = 4.4, 4.4 Hz), 2.98 (dd, 1H, *J* = 9.0, 7.5 Hz), 2.93 (br s, 1H), 2.39- 2.37 (m, 2H), 2.34- 2.28 (m, 2H); $^{13}$C NMR ($CDCl_3$) 100 MHz δ 173.6, 136.7, 134.0, 117.5, 115.8, 102.7, 85.7, 83.7, 73.6, 70.4, 69.9, 63.5, 61.1, 60.5, 33.6, 28.9; TOF HRMS (DART) *m/z* calc'd for $C_{16}H_{27}O_7$ $[M+H]^+$ 331.1757, obs. 331.1727.

**Allyl 2,3-di-*O*-methyl-6-*O*-(5-hexenoyl)-α-D-glucopyranoside (10c).** The diene was isolated as a clear, colorless oil (0.186 g, 0.542 mmol, 54%). $R_f$ 0.42 (1:1, Hex:EtOAc); $[\alpha]_D$ +34.5° (*c* 2.5, $CHCl_3$); $^1$H NMR ($CDCl_3$) 400MHz δ 5.90 (dddd, 1H, *J* = 17.0, 10.4, 6.6, 5.4 Hz), 5.72 (dddd, 1H, *J* = 17.0, 10.2, 6.7, 6.7 Hz), 5.31-5.18 (m, 2H), 5.00- 4.92 (m, 3H), 4.37 (dd, 1H, *J* = 12.1, 4.9 Hz) 4.22 (dd, 1H, *J* = 12.1, 2.2 Hz) 4.15 (dd, 1H, 12.8, 5.2 Hz), 4.04- 4.00 (m, 1H), 3.75 (ddd, 1H, *J* = 9.9, 4.8, 2.2 Hz), 3.59 (s, 3H), 3.47 (d, 1H, *J* = 12.4 Hz), 3.42 (s, 3H), 3.34- 3.23 (m, 1H), 3.17 (dd, 1H, *J* = 9.4, 3.6 Hz), 2.89 (d, 1H, *J* = 3.2 Hz), 2.35- 2.30 (m, 2H), 2.09- 2.02 (m, 2H), 1.70 (m, 2H); $^{13}$C NMR ($CDCl_3$) 100 MHz δ 174.0, 137.7, 133.6, 118.6, 115.7, 95.2, 82.7, 81.8,70.2, 69.7, 68.4, 63.3, 61.4, 58.4, 33.5, 33.1, 24.1; TOF HRMS (DART) *m/z* calc'd for $C_{17}H_{29}O_7$ $[M+H]^+$ 345.1913, obs. 345.1902; for $C_{17}H_{32}NO_7$ $[M+NH_4]^+$ 362.2179, obs. 362.2184.

**Ring closing metathesis.** To a solution of diene dissolved in dry toluene (5-10 mM) under $N_2$ was added Grubbs II catalyst or Hoyveda-Grubbs catalyst (5 mol%). The reaction was then heated to reflux for 12-16 hours or until starting material was consumed as observed through TLC. The reaction was allowed to cool to room temperature and then the solvent was removed under reduced pressure. The mixture was then purified via column chromatography (7:3 Hex:EtOAc)

**α-[12]-Macrolactone (5).** Light tan crystalline solid (0.084 g, 0.253 mmol, 55%). m.p. 115.7-117.6 °C; $R_f$ 0.26 (1:1 Hex:EtOAc); $[\alpha]_D$ +99.4° (c 0.83, $CHCl_3$); $^1$H NMR ($CDCl_3$) 400MHz δ 5.76- 5.70 (m, 1H), 5.66 – 5.59 (m, 1H), 5.03 (d, 1H, *J* = 3.6 Hz), 4.84- 4.76 (m, 1H), 4.53 (dd, 1H, *J* = 12.6, 3.9 Hz), 3.96- 3.88 (m, 2H), 3.83 (dd, 1H, *J* = 12.5, 9.4 Hz), 3.60 (s, 3H), 3.45 (s, 3H), 3.38 (dd, 1H, *J* = 9.2, 9.2 Hz), 3.21- 3.14 (m, 2H), 2.47- 2.37 (m, 5H); $^{13}$C NMR ($CDCl_3$) 100 MHz δ 173.1, 131.1, 130.3, 99.6, 83.1, 82.2, 73.4, 71.5, 69.5, 64.4, 61.5, 58.5, 33.8, 29.5; TOF HRMS (DART) *m/z* calc'd for $C_{14}H_{23}O_7$, $[M+H]^+$ 303.1444, obs. 303.1434; for $C_{14}H_{26}NO_7$ $[M+NH_4]^+$ 320.1709, obs. 320.1694.

**β-[12]-Macrolactone (6).** Pale brown solid (66%). m.p. 120.9-123.1 °C; $R_f$ 0.35 (1:1 Hex:EtOAc); $[\alpha]_D$ -68.9° (*c* 0.60, $CHCl_3$); $^1$H NMR ($CDCl_3$) 400MHz δ 5.68 (ddd, 1H, *J* = 14.2, 10.4, 2.7 Hz), 5.48- 5.41 (m, 1H), 4.80 (dd, 1H, *J* = 11.3, 10.0 Hz), 4.47 (ddd, 1H, *J* = 12. 9, 3.4, 1.7 Hz), 4.16 (d, 1H, *J* = 7.4 Hz), 3.95 (dd, 1H, *J* = 11.2, 2.0 Hz), 3.79 (dd, 1H, *J* = 12.9, 10.3 Hz), 3.59 (s, 3H), 3.52 (s, 3H), 3.34- 3.31 (m, 2H), 3.10- 3.04 (m, 2H), 2.50- 2.26 (m, 5H); $^{13}$C NMR ($CDCl_3$) 100 MHz δ 173.0, 132.8, 129.0, 106.1, 86.6, 83.7, 74.3, 73.2, 71.1, 62.8, 61.0, 60.3, 34.7, 29.2; TOF HRMS (DART) *m/z* calc'd for $C_{14}H_{23}O_7$ $[M+H]^+$ 303.1444, obs. 303.1436; for $C_{14}H_{26}NO_7$ $[M+NH_4]^+$ 320.1709, obs. 320.1695.

**α-[13]-Macrolactone (19).** Brown amorphous solid (45%) m.p. 71.2-74.1 °C; $R_f$ 0.21 (1:1, Hex:EtOAc); $^1$H NMR ($CDCl_3$) 400MHz δ 5.70-5.60 (m, 3H), 5.54-5.48 (m, 1H), 4.99 (d, 1H, *J* = 3.4 Hz), 4.85 (ddd, 2H, *J* = 11.7, 10.1, 4.1 Hz), 4.17-3.94 (m, 4H), 3.89-3.81 (m, 4H), 3.61 (s, 3H), 3.60 (s, 3H), 3.65-3.60

(m, 2H), 3.49-3.44 (m, 1H), 3.46 (s, 3H), 3.45 (s, 3H), 3.42-3.33 (m, 2H), 3.20-3.14 (m, 4H), 2.60-2.52 (m, 2H), 2.44-2.30 (m, 5H), 2.20-2.11 (m, 3H), 1.89-1.84 (m, 2H); 400MHz δ $^{13}$C NMR ($CDCl_3$) 100 MHz δ 173.8, 173.6, 133.6, 130.5, 130.5, 128.4, 99.5, 97. 2, 83.5, 83.1, 82.1, 81.5, 73.8, 72.9, 71.8, 71.5, 71.2, 70.1, 64.6, 64.6, 61.5, 58.7, 58.5, 35.3, 34.0, 32.5, 32.0, 31.8, 29.1, 22.8; HRMS [M+H]+ *m/z* for $C_{15}H_{25}O_7$, calc'd 317.1600, obs. 317.1577, [M+NH4]+ $C_{15}H_{28}NO_7$ calc'd 334.186, obs. 334.1847.

***tert*-Butyldimethylsilyl-α-[12]-macrolactone (11).** To **5** (0.025 g, 0.08 mmol) dissolved in dry DMF (3 mL) was added *tert*-butyldimethylsilyl chloride (0.011 g, 0.11 mmol) and imidazole (0.01 g, 0.08 mmol). The reaction was allowed to stir overnight (12 h) and then the reaction was then diluted with ether (50 mL) and washed with brine (2 x 25 mL). The ether layer was collected, dried, and concentrated under reduced pressure. Column chromatography with Hex:EtOAc (1:1) yielded a clear colorless oil **11** (0.027 g, 75%). $[\alpha]_D$ +185.2° (*c* 0.11, $CHCl_3$); $^1$H NMR ($CDCl_3$) 400MHz δ 5.79 (ddd, 1H, J = 15.1, 9.6, 4.4 Hz), 5.67-5.60 (m, 1H), 5.02 (d, 1H, *J* = 3.8 Hz), 4.75 (d, 1H, *J* = 9.2 Hz), 4.66 (dd, 1H, J = 12.6, 4.3 Hz), 3.88-3.81 (m, 3H), 3.53 (s, 3H), 3.48 (s, 3H), 3.30 (dd, 1H, J = 8.7, 8.7 Hz), 3.21 (dd, 1H, J = 8.9, 8.9), 3.15 (dd, 1H J = 9.5, 3.8 Hz), 2.48 – 2.36 (m, 3H), 1.56 (s, 1H), 1.25 (s, 1H), 0.93 (s, 9H), 0.11 (s, 3H), 0.06 (s, 3H); $^{13}$C NMR ($CDCl_3$) 100 MHz δ 173.0, 131.0, 130.5, 99.5, 83.4, 82.9, 73.6, 72.6, 70.3, 64.7, 61.6, 58.8, 33.8, 29.3, 26.1, 18.3.

**α-[12]-Macrolactone (12).** To an ice cold solution of **5** (0.049 g, 0.16 mmol) in dry DMF (5 mL) was added a 60% dispersion of NaH in oil (0.0080 g, 0.19 mmol). Tetrabutyl ammonium iodide (0.017 g, 0.047 mmol) was added to the mixture followed by benzyl bromide (0.022 mL, 0.19 mmol). The reaction was then allowed to warm to rt with monitoring by TLC. When starting material was no longer visible by TLC, the reaction was quenched by addition of water (1 mL). After, the solvent was removed under reduced pressure and the mixture was redissolved in DCM (5 mL) and washed with brine (2 x 5 mL) and dried over sodium sulfate. The solution was filtered and the solvent removed under reduced pressure. Column chromatography on the residue (4:1 Hex:EtOAc) yielded **12** (0.044 g, 70%) as a white solid. m.p. 123.5-125.7 °C; $R_f$ 0.33 (7:3, Hex:EtOAc); $[\alpha]_D$ -105.8° (*c* 0.38, $CHCl_3$); $^1$H NMR ($CDCl_3$) 400MHz δ 7.37-7.26 (m, 5H), 5.77-5.67 (m, 1H), 5.64-5.67 (m, 1H), 5.00 (d, 1H, *J* =5.1 Hz), 4.86 (d, 1H, *J* = 15.0 Hz), 4.62 (dd, 1H, *J* = 14.6, 2.1), 4.57 (d, 1H, *J* = 15.0 Hz), 4.53 (dd, 1H, *J* = 13.1, 5.6 Hz), 3.94 (ddd, 1H, *J* = 15.4, 13.5, 2.1 Hz), 3.86-3.77 (m, 2H), 3.61 (s, 3H), 3.55 (d, 1H, *J* = 12.0 Hz), 3.49 (s, 3H), 3.15 (dd, 1H, *J* = 12.9, 5.1 Hz), 3.08 (dd, 1H, *J* = 13.3, 11.7 Hz), 2.44-2.35 (m, 4H); $^{13}$C NMR ($CDCl_3$) 100 MHz δ 172.8, 138.2, 131.0, 130.4, 128.7, 128.3, 128.1, 99.6, 83.8, 82.3, 78.9, 74.8, 73.5, 69.2, 64.1, 61.3, 59.1, 33.7, 29.4.

**(α-Tetrahydropyranyl)-α-[12]-macrolactone (13) and (β-tetrahydropyranyl)-α-[12]-macrolactone (14).** To a solution of **5** (0.021 g, 0.069 mmol) in dry DCM (5 mL) was added dihydropyran (0.01 mL, 0.010 mmol) and pyridinium *p*-toluenesulfonate (0.005 g, 0.002 mmol). The reaction was allowed to stir for 4 h at rt. The solution was diluted with ether (20 mL) and washed with brine (2 x 20 mL). The

combined organic layers were collected, dried with $Na_2SO_4$, and concentrated under educed pressure. The residue was purified via column chromatography (7:3, Hex:EtOAc) to yield 5d and 5e as clear, colorless oil/glasses. The stereogenic centers on the THP moiety of **13** and **14** were assigned based on analogy to $^{13}$C chemical shifts reported for a THP-protected estradiol [1].

**(α-Tetrahydropyranyl)-α-[12]-macrolactone (13).** The first fraction was **13** (0.011 g, 41%). $R_f$ 0.27 (7:3, Hex:EtOAc); $[\alpha]_D$ +20.7° (*c* 0.27, $CHCl_3$) $^1$H NMR ($CDCl_3$) 400MHz δ 5.75 (ddd, 1H, J = 15.1, 9.5, 4.2 Hz), 5.66-5.59 (m, 1H), 5.01 (d, 1H, J = 3.8 Hz), 4.90 (d, 1H, *J* = 10.2 Hz), 4.78-4.74 (m, 1H), 4.54 (dd, 1H, *J* = 12.6, 4.2 Hz), 3.98-3.88 (m, 3H), 3.83 (dd, 1H, *J* = 12.6, 9.5 Hz), 3.56 (s, 3H) 3.51- 3.42 (m, 5H), 3.33 (dd, 1H, *J* = 9.5, 9.5 Hz), 3.13 (dd, 1H, *J* = 9.5, 3.8 Hz), 2.48- 2.35 (m, 4H), 1.82-1.73 (m, 2H), 1.55-1.47 (m, 4H); $^{13}$C NMR ($CDCl_3$) 100 MHz δ 173.1, 131.0, 130.4, 101.7, 99.7, 83.7, 82.3, 77.4, 76.5, 73.5, 68.9, 64.6, 61.4, 59.1, 33.8, 31.6, 29.4, 25.5, 21.1; TOF HRMS (DART) *m/z* calc'd for $C_{19}H_{31}O_8$ $[M+H]^+$ 387.2019, obs. 387.2023; for $C_{19}H_{34}NO_8$ $[M+NH_4]^+$ 404.2284, obs. 404.2246.

**(β-Tetrahydropyranyl)-α-[12]-macrolactone (14).** The second fraction was **14** (0.007 g, 27%). $R_f$ 0.16 (7:3, Hex:EtOAc); $^1$H NMR ($CDCl_3$) 400MHz δ 5.76 (ddd, 1H, *J* = 15.2, 9.4, 4.4 Hz), 5.67 – 5.56 (m, 1H), 5.00 (d, 1H, *J* = 3.8 Hz), 4.69 -4.65 (m, 1H), 4.60 (dd, 1H, *J* = 3.8, 3.8 Hz), 4.55 (dd, 1H, *J* = 12.6, 4.5 Hz), 4.06 (ddd, 1H, *J* = 11.9, 8.2, 3.6 Hz), 3.96 – 3.91 (m, 2H), 3.84 (dd, 1H, *J* = 12.6, 9.4 Hz), 3.61 (s, 3H), 3.51 (s, 3H), 3.53 – 3.45 (m, 2H), 3.33 – 3.27 (m, 1H), 3.18 (dd, 1H, *J* = 9.6, 3.9 Hz), 2.49-2.35 (m, 4H), 1.89 – 1.62 (m, 3H), 1.59-1.49 (m, 3H); $^{13}$C NMR ($CDCl_3$) 100 MHz δ 173.0, 131.1, 130.4, 100.0, 99.7, 82.4, 82.0, 77.1, 73.5, 69.9, 64.0, 63.0, 61.4, 59.4, 33.8, 31.1, 29.4, 25.6, 19.8; TOF HRMS (DART) *m/z* calc'd for $C_{19}H_{31}O_8$ $[M+H]^+$ 387.2019, obs. 387.2071; for $C_{19}H_{34}NO_8$ $[M+NH_4]^+$ 404.2284, obs. 404.2297.

**β-Alanyl-α-[12]-macrolactone (15).** To a solution of **5** (0.030 g, 0.11 mmol) in dry DCM (10 mL) at 0 °C was sequentially added 1.2 eq. of Boc-β-alanine (0.024 g, 0.13 mmol), 1.2 eq. N,N'-dicyclohexylcarboimide (0.032 g , 0.13 mmol) and 0.5 eq. of dimethylaminopyridine (0.006 g, 0.05 mmol).The mixture was allowed to come to room temperature over 2h while being monitored by TLC. When starting material had been consumed, the reaction was diluted with hexanes (10 mL) and filtered through a pad of celite. The combined filtrates were concentrated under reduced pressure. The residue was purified by column chromatography eluting with 4:1 Hex:EtOAc to yield the Boc-protected analog of "**Boc-15**" (0.028 g, 54%). $R_f$ 0.48 (1:1, Hex:EtOAc) $^1$H NMR ($CDCl_3$) 400MHz δ 5.76-5.61 (m, 2H), 5.20 (b, 1H), 5.03 (d, 1H, *J* = 3.2), 4.65 (dd, 1H, *J* = 10.5 Hz), 4.53 (dd, 1H, *J* = 12.5, 4.0 Hz), 4.40 (d, 1H, *J* = 11.5 Hz), 3.96 (t, 1H, *J* = 9.4 Hz), 3.84 (dd, 1H, J = 12.4, 8.9 Hz), 3.77 (t, 1H, *J* = 11.0 Hz), 3.55-3.40 (m, 9H), 3.21 (dd, 1H, *J* = 9.7, 3.8 Hz), 2.61 (ddd, 2H, *J* = 11.6, 5.8, 5.8 Hz), 2.39 (4H) 1.40 (s, 9H); $^{13}$C NMR ($CDCl_3$) 100 MHz δ 173.3, 172.0, 156.1, 131.5, 130.0, 99.7, 81.9, 80.6, 79.6, 73.4, 71.9, 68.0, 64.0, 61.2, 59.4, 36.5, 35.1, 34.2, 33.7, 29.7, 28.6, 25.9. The Boc group was removed using the following procedure. **Boc-15** (0.028 g, 0.06 mmol) was dissolved into 1:1 solution of DCM (5 mL) and trifluoroacetic acid (5 mL) and allowed to stir under $N_2$ at room temperature. Upon disappearance

of starting material on TLC, the solvent was removed under reduced pressure to yield **15** as a yellow oil (0.026 g, 92%). $R_f$ 0.21 (1:1 Hex:EtOAc); $[\alpha]_D$ +52.8° (*c* 0.33, $CHCl_3$); $^1H$ NMR ($CD_3OD$) 400MHz δ 5.67-5.56 (m, 2H), 5.03 (d, 1H, *J* = 3.44 Hz), 4.56 (t, 1H, *J* = 9.6), 4.33 (d, 2H, *J* = 11.6 Hz), 3.84 (m, 2H), 3.69 (t, 1H, *J* = 10.7 Hz), 3.44-3.35 (m, 7H), 3.21 (br s, 2H), 3.18 (m, 3H), 2.75 (ddd, 2H, *J* = 17.7, 6.4, 6.4 Hz), 2.31 (m, 4H). $^{13}C$ NMR ($CD_3OD$) 100 MHz δ 173.7, 171.2, 131.6, 129.9, 99.4, 81.7, 80.5, 73.3, 72.3, 67.7, 61.1, 59.2, 33.5, 33.3, 29.5, 25.4; TOF HRMS (DART) *m/z* calc'd for $C_{17}H_{28}NO_8$ $[M+H]^+$ 374.1815, obs. 374.1828.

**(β-D-Desosaminyl)-α-[12]-macrolactone (16).** *Glycosylation:* AgOTf (0.134 g, 0.521 mmol, 4.0 eq.) was added to a suspension of 4Å molecular sieves (0.400 g) in dry DCM (3 mL) at 0 °C in darkness (wrapping with aluminum foil). *S*-pyrimidinyl 2-*O*-methoxycarbonyl desoamine [2] (0.064 g, 0.196 mmol, 1.5 eq.) in dry DCM (1.5 mL) and macrocycle **5** (0.039 g, 0.130 mmol, 1.0 eq.) in dry DCM (1.5 mL) were added to the solution of AgOTf at the same time via syringe. The mixture was stirred for 2 h at 0 °C and then quenched with sat. $NaHCO_3$ (0.5 mL). The reaction mixture was then diluted with EtOAc (30 mL) and filtered through a pad of celite. Aqueous layer was extracted with additional (2 x 10 mL) EtOAc. The organic layers were combined, washed with brine (1 x 20 mL), dried over $Na_2SO_4$, and concentrated in-vacuo. The residue was purified by flash-chromatography to give the product as colorless oil/film 0.064 g (95%). *Deprotection:* The protected, glycosylated macrocycle (0.064 g, 0.124 mmol) was taken up in a mixture of MeOH (25 mL) and $H_2O$ (2.75 mL) and refluxed for 8 h. The mixture was allowed to cool to rt and diluted with diethyl ether (60 mL). The ether was washed with sat. NaCl (60 mL) and the aqueous layer was extracted with additional diethyl ether (2 x 40 mL). The combined organic layers were washed with sat. NaCl (60 mL), dried over $Na_2SO_4$, and concentrated in-vacuo. This residue was purified by flash chromatography yield to provide 0.042 g (73%) **16** as a clear colorless oil/film. $[\alpha]_D$ +50.1° (*c* 0.70, $CHCl_3$); $^1H$ NMR (400MHz, $CDCl_3$) δ 5.81 – 5.73 (m, 1H), 5.69 – 5.61 (m, 1H), 5.07 – 4.99 (m, 2H), 4.56 (dd, 1H, *J* = 4.0, 12.4 Hz), 4.37 (d, 1H, *J* = 7.3 Hz), 4.08 – 3.99 (m, 2H), 3.86 (dd,1H, *J* = 9.5, 12.6 Hz), 3.66 – 3.61 (m, 4H), 3.56 – 3.50 (m, 4H), 3.41 – 3.34 (m, 2H), 3.28 – 3.14 (m, 2H), 2.75 – 2.66 (m, 1H), 2.49 – 2.36 (m, 1H), 1.84 (d, 1H *J* = 11.7 Hz), 1.34 – 1.27 (m, 4H); $^{13}C$ NMR (100 MHz, $CDCl_3$) δ 173.2, 131.0, 130.4, 105.5, 99.4, 82.4, 82.0, 79.4, 73.3, 70.6, 69.9, 69.6, 65.4, 64.5, 61.4, 59.2, 40.8, 33.7, 29.8, 29.3, 21.5; TOF HRMS (DART) m/z calcd for $C_{22}H_{38}NO_9$ $[M+H]^+$ 460.2547, found 460.2540.

***tert*-Butyldimethylsilyl-β-[12]-macrolactone (18).** This material was isolated as a clear, colorless oil (0.017 g, 83%). $R_f$ 0.38 (1:1, Hex:EtOAc); $[\alpha]_D$ -114.2° (*c* 0.86, $CHCl_3$); $^1H$ NMR ($CDCl_3$) 400MHz δ 5.67 (ddd, 1H, *J* = 14.7, 10.1, 3.2 Hz) 5.47-5.40 (m, 1H) 4.72 (dd, 1H, *J* = 10.0, 10.0 Hz) 4.53-4.49 (m, 1H) 4.13 (d, 1H, *J* = 7.6 Hz) 3.97-3.76 (m, 2H) 3.52 (s, 3H) 3.51 (s, 3H) 3.34-3.23 (m, 2H) 3.02 (m, 2H) 2.50-2.28 (m, 4H) 0.86 (s, 9H), 0.08 (s, 3H), 0.02 (s, 3H). $^{13}C$ NMR ($CDCl_3$) 100 MHz δ 173.0, 132.8, 129.0, 110.2, 106.0, 87.1, 84.7, 74.4, 74.3, 72.2, 63.1, 61.3, 60.4, 56.2, 34.7, 25.2, 26.1; TOF HRMS

(DART) *m/z* calc'd for $C_{20}H_{37}O_7Si$ $[M+H]^+$ 417.2309, obs. 417.2349; for $C_{20}H_{40}NO_7Si$ $[M+NH_4]^+$ 434.2574, obs. 434.2550.

**α-[12]-Macrolactone (17).** Macrocycle **5** (0.027 g, 0.090 mmol was dissolved in absolute methanol (10 mL) and to this solution was added 10% Pd/C (0.003 g). The vessel was purged with $N_2$ and then an atmosphere of $H_2$ was introduced. The reaction was allowed to stir overnight (12 h). The $H_2$ atmosphere was removed and the solution was then passed through a bed of celite which was then washed with hexanes (25 mL). The filtrate was collected and concentrated under reduced pressure to yield a clear colorless oil/film (0.020 g, 72%). $R_f$ 0.32 (7:3, Hex:EtOAc); $[\alpha]_D$ +46.3° (*c* 0.44, $CHCl_3$); $^1H$ NMR ($CDCl_3$) 400MHz δ 5.08 (dd, 1H, *J* = 11.3, 1.5) 4.91 (d, 1H, *J* = 4.0 Hz) 3.94-3.89 (m, 2H) 3.78-3.71 (m, 2H) 3.60 (s, 3H) 3.46 (s, 3H) 3.33-3.21 (m, 3H) 2.52 (ddd, 1H, *J* = 4.4, 7.0, 13.2 Hz) 2.35 (br s, 1H) 2.25 (ddd, 1H, *J* = 13.9, 9.6, 4.6) 1.86-1.76 (m, 1H) 1.73-1.63 (m, 3H) 1.51-1.43 (m, 2H). $^{13}C$ NMR ($CDCl_3$) 100 MHz δ 173.8, 98.5, 84.0, 82.1, 71.1, 70.9, 63.6, 61.6, 58.6, 34.2, 28.7, 24.0, 23.6; TOF HRMS (DART) *m/z* calc'd for $C_{14}H_{25}O_7$ $[M+H]^+$ 305.1600, obs. 305.1572; for $C_{14}H_{28}NO_7$ $[M+NH_4]^+$ 322.1866, obs. 322.1839.

**Determination of minimum inhibitory concentration (MIC)**

Antibacterial and antifungal potency was measured in 96-well plate-based microbroth dilution assays as previously described in the literature [3]. Test compounds were prepared as stock solutions in DMSO (50 mM) and stored at -20 °C until used. Each compound was diluted in a 2-fold dilution series, and a small sample (1 μL) of each was added to wells in a test plate so that each column contained the dilution series for one compound. An inoculum (~$1 \times 10^5$ cfu) of a test organism in culture media (100 μL) was added to each well resulting in a dilution series running from 500 to 2 μM. Where necessary the measurements were repeated at lower concentrations of the test compound. After an incubation period determined from the strain-specific doubling time, Alamar blue (10 μL) was added and allowed to incubate; each well was scored for dye reduction [4]. The MIC value was taken as the lowest concentration of test compound that inhibits growth such that less than 1% reduction of the blue resazurin ($\lambda_{max}$ 570 nm) component of the Alamar blue to the pink resorufin ($\lambda_{max}$ 600 nm) was observed.

**References**

1. Bocheau, V.; Renaud, M.; Rolland de Ravel, M.; Mappus, E.; Cuilleron, C. Y. *Steroids* **1990**, *55*, 209-221.
2. Breton, P; Hergenrother, P.J.; Hida, T.; Hodgson, A.; Judd, A. S.; Kraynack, E.; Kym, P. R.; Lee, W.-C.; Loft, M. S.; Yamashita, M.; Martin, S. F. *Tetrahedron* **2007**, *63*, 5709-5729.
3. Kusche, B.R.; Smith, A. E.; McGuirl, M. A.; Priestley, N. D. *J. Am. Chem. Soc.* **2009**, *131*, 17155-17165.
4. Davey, K. G.; Szekely, A.; Johnson, E. M.; Warnock, D. W. *J. Antibiot. Chemother.* **1998**, *42*, 439–444.
